# Identification of a Pyroptosis-Related Gene Signature for Prediction of Overall Survival in Lung Adenocarcinoma

**DOI:** 10.1155/2021/6365459

**Published:** 2021-09-30

**Authors:** Zheng Dong, Lv Bian, Minglang Wang, Luoqing Wang, Yilian Wang

**Affiliations:** ^1^Department of Cardiology, The Second People's Hospital of Lianyungang, Lianyungang, Jiangsu 222023, China; ^2^Hospice Care Center, The Second People's Hospital of Lianyungang, Lianyungang, Jiangsu 222023, China

## Abstract

Pyroptosis is a kind of programmed cell death that is characterized by inflammation. However, the expression of pyroptosis-related genes and their connection with prognosis in lung adenocarcinoma (LUAD) remain unknown. The aim of this study is to create and validate a LUAD prediction signature based on genes associated with pyroptosis. The TCGA and GEO were used to collect gene sequencing data and clinical information for LUAD samples. To identify patients with LUAD from the TCGA cohort, consensus clustering by pyroptosis-related genes was employed. Our prognostic model was constructed using LASSO-Cox analysis after Cox regression using differentially expressed genes. To predict patient survival, we created a seven-mRNA signature. Additionally, reliability and validity were established in the GEO cohort. To assess its diagnostic and prognostic usefulness, an integrated bioinformatics method was used. Using a risk score with varying overall survival (OS) in two cohorts (all *p* < 0.001), a seven-gene signature was developed to categorize patients into two risk categories. The signature was shown to be an independent predictor of LUAD using multivariate regression analysis. The signature was linked to a variety of immune cell subtypes according to a study of immune cell infiltration. We constructed a signature consisting of seven genes as a robust biomarker with potential for clinical use in risk stratification and OS prediction in LUAD patients, as well as a potential indicator of immunotherapy in LUAD.

## 1. Introduction

Worldwide, lung cancer causes a significant percentage of cancer mortality and has a bad prognosis [1, 2]. Lung cancer accounted for more than a quarter (27%) of all cancer fatalities in 2015 [3, 4]. Clinically, LUAD is most often diagnosed in patients with nonsmall cell lung cancer (NSCLC), while the most prevalent histologic form of NSCLC accounts for 40% of all lung cancer cases [5]. Surgical excision, chemotherapy, and radiation are the primary clinical therapies for LUAD at the moment. Despite advances in medical therapies in recent years, only 15% of LUAD patients survive for more than 5 years [6, 7]. Numerous studies have shown that LUAD is a very diverse illness with unique genetic and transcriptome features among individual individuals, and predicting LUAD prognosis remains difficult. As a result, it is critical to discover novel prognostic gene signatures that may be utilized to generate prognostic predictions and act as new therapeutic targets for LUAD patients.

Pyroptosis is the cleavage of gasdermins through conventional and nonclassical mechanisms, which may cause cells to expand indefinitely until the cell membrane ruptures, allowing the contents of the cell to escape, resulting in a severe inflammatory reaction [8, 9]. Pyroptosis is critical for combating infection and endogenous danger signals. Pyroptosis, by releasing inflammatory chemicals, provides a tumor-suppressive environment; yet, it may also impair the body's immunological response to tumor cells and promote tumor development in certain malignancies [10, 11]. However, it is unknown that how pyroptosis affects the prognosis of LUAD.

The use of next-generation sequencing to classify LUAD patients is a new technique for rapidly identifying cancer features and determining the best therapy options. Although EGFR tyrosine kinase inhibitors (TKIs) and PD-L1/PD1 immune checkpoint targeting treatments have been shown effective, the underlying mechanisms that contribute to the lack of efficacy in patients with LUAD or even complete unresponsiveness to these therapies have still to be identified [12, 13]. According to the current understanding, molecular subtypes cannot be used as guides in clinical therapy due to the absence of subgroup classifications. As a result, the creation of a reliable gene signature to predict prognosis and guide therapeutic treatment, particularly in the areas of targeted therapy and immunotherapy, is critical.

The goal of this research was to develop a model for predicting prognosis and guiding therapeutic therapy by categorizing LUAD patients based on genes associated with pyroptosis. After grouping 414 people with LUAD based on genes associated with pyroptosis, we identified two subgroups that were connected to prognosis. The risk score may then be calculated by utilizing the LASSO-Cox technique to build a model associated with pyroptosis. This risk score may forecast prognosis and immunological infiltration. Our results suggest a possible link between pyroptosis, prognosis, and the immunological milieu of individuals with LUAD.

## 2. Materials and Methods

### 2.1. Data Collection

We conducted a comprehensive search of publicly accessible transcriptome cohorts for LUAD with matching clinical data. The TCGA database was used to obtain normalized gene expression data from RNA sequencing (FPKM), somatic mutation data, and clinical data from TCGA-LUAD cohort. Using the total number of mutations identified in the exome content, the tumor mutation burden (TMB) was then calculated per megabase for each sample. The GEO database was also used to gather clinical information and normalized gene expression data; the accession number is GSE13507. R was used to examine the data (version 4.0.2).

### 2.2. Identification of Pyroptosis-Related Genes with Variable Expression

From previous studies [14–17], we retrieved 52 pyroptosis-related genes, which are listed in Table S1. In order to discover the genes that were differentially expressed between normal and tumor tissues, we collected 19 normal lung tissues as well as 414 LUAD tissues. DEGs with a *p* value of 0.05 were identified using the “limma” package. A heatmap was then utilized to compare the gene expression associated with pyroptosis in LUAD versus normal lung tissues.

### 2.3. Consensus Clustering

Consensus clustering using *k*-means algorithms was used to discover unique pyroptosis-related patterns pertaining to gene expression [18]. The number and stability of clusters were established using the consensus clustering method, which was implemented in the “ConsensuClusterPlus” package. We repeated our categorization 1,000 times to ensure its stability [19].

### 2.4. The Development and Validation of a Predictive Pyroptosis-Related Pattern Gene Signature

With an adjusted *p* value of 0.05 and an absolute value of |log_2_FC| ≥ 1, in TCGA cohort, DEGs between pyroptosis-related patterns were identified. Using the univariate Cox method, the DEGs with prognostic significance were identified, and the *p* value was corrected using the Benjamini & Hochberg (BH) correction approach. A prognostic model was built using LASSO-Cox regression analysis to minimize the risk of overfitting [20, 21]. The LASSO method was used to select and minimize variables. A regression analysis was performed using the normalized expression matrix of potential prognostic DEGs as an independent variable, with the OS and status of patients in the TCGA cohort serving as the dependent variables. Patients' risk scores were computed using a method that included finding a patient's level of expression for each pattern gene associated with pyroptosis and the regression coefficient associated with that level. The formula was devised as follows:

(1) $$\begin{array}{c} \text{risk score}=\sum_{i=1}^{n}\exp\;i*\beta i. \end{array}$$

Patients were classified into high-and low-risk categories based on their median risk score. In order to investigate the distribution of various groups in terms of gene expression levels in the constructed model, PCA and t-SNE analyses were carried out using the “Rtsne” and “ggplot2” R packages, respectively [22]. Using the “survminer” R package, a survival analysis was performed to compare the OS of high-and low-risk groups. Survival and TimeROC packages were used to perform time-dependent ROC curve analysis, which was used to assess the prognostic signature's predictive value. The signature's prognostic significance was further investigated using univariate and multivariate Cox analysis.

### 2.5. Analysis of Functional Enrichment of DEGs between Patterns

Consensus clustering classified LUAD patients in the TCGA cohort into two categories. The software “clusterProfiler” was used to perform GO and KEGG analysis on the DEGs. The ssGSEA was performed using the “gsva” package to compute infiltrating immune cell scores and assess immune-related pathway activity [23, 24].

### 2.6. Predictive Nomogram Establishment

A nomogram was developed by taking into consideration the patient's risk score and other clinicopathological features and then using that information to provide a reliable clinical prediction tool for LUAD patients, especially with regard to their 1-, 3-, and 5-year survival. Following that we conducted calibration curve analyses to determine the suitability of our nomogram for clinical use.

### 2.7. Statistical Analysis

For statistical analyses, R software version 4.0.2 and various R packages were utilized, with a 2-tailed *p* value of 0.05 indicating statistical significance. The “survival” package was used to run univariate and multivariate Cox regression analysis. The “glmnet” package was used to perform the LASSO-Cox regression analysis, and ten times cross-validations were employed to find the optimum penalty parameter lambda. The “survival” package was used to create Kaplan–Meier analyses and survival curves. The nomogram and calibration curve were created using the “rms” package. The “timeROC” software was used to analyze time-dependent ROC curves. Based on FDR, the Benjamini–Hochberg technique was employed to identify differentially expressed genes. Using “GSVA,” the ssGSEA-normalized DEGs were compared with a genome.

## 3. Results

### 3.1. An Overview of Genetic Alterations and Expression Variations in Pyroptosis-Related Genes in LUAD

**Figure 1:**
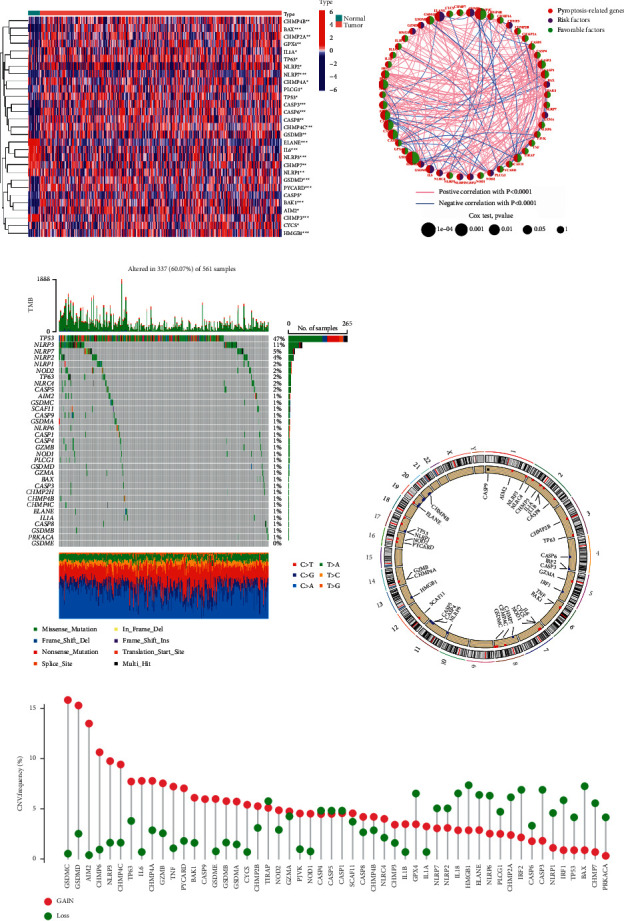
Genes associated with pyroptosis in LUAD have distinct characteristics and differences. (a) Heatmap depicting the variations in pyroptosis-related gene expression between the normal and tumor samples. (b) A network of correlations including pyroptosis-related genes in the TCGA cohort. (c) The mutation landscape in 561 LUAD patients from the TCGA-LUAD cohort. Each pyroptosis-related gene's mutation information was displayed by a waterfall plot. Annotations at the bottom of the corresponding hues denoted various mutation types. The barplot above depicted the mutation load. Individual mutation frequencies were indicated by the right values. (d) The TCGA cohort's location of CNV modification of genes associated with pyroptosis on chromosomes. (e) Pyroptosis-related gene CNV frequency in the TCGA cohort. The height of the columns revealed various proportions. Copy number variants, or CNVs, are a kind of copy number variation.

In order to compare the pyroptosis-related gene expression levels found in the TCGA data from 19 normal and 414 tumor tissues, we first calculated the 52 pyroptosis-related gene expression levels and then looked for the DEGs that corresponded to them. 6 genes were found to be downregulated, while 23 genes were found to be enriched in the tumor group.  Figure 1(a) shows heatmaps of these genes' RNA levels.  Figure 1(b) depicts the correlation network including all pyroptosis-related genes. At the genetic level, 337 of the 561 samples (approximately 60.07%) had mutations in pyroptosis-related genes (Figure 1(c)). We also discovered CNVs in pyroptosis-related genes, which were frequent alterations with a focus on copy number amplification (Figure 1(e)). We discovered changes in pyroptosis-related genes with CNVs on the chromosome (Figure 1(d)). We predicted that alterations in CNV might play a significant role in causing aberrant gene expression. Finding that pyroptosis-related genes had a relationship with the LUAD suggests that they may reflect unique features in patients in our research.

### 3.2. Tumor Classification Based on the DEGs

**Figure 2:**
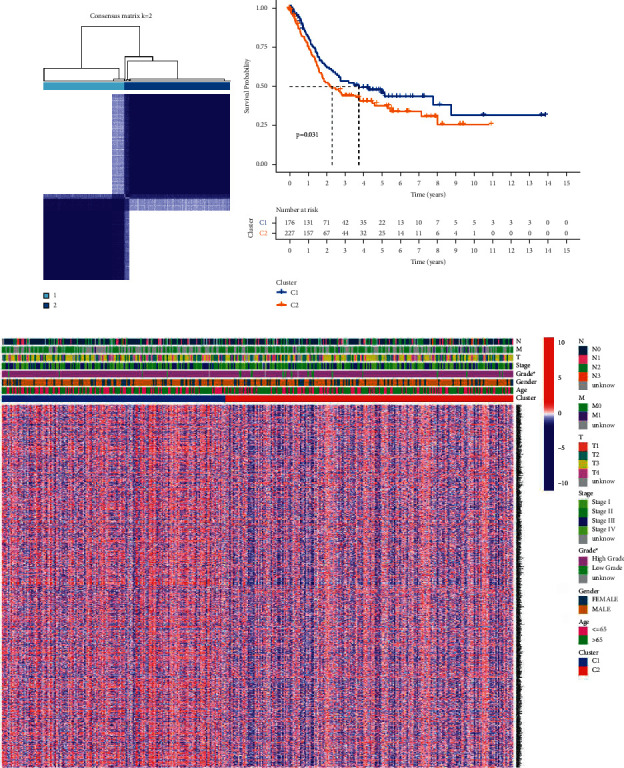
Subgroups of LUAD defined by genes involved in pyroptosis. (a) The TCGA cohort's consensus score matrix for all samples when *k* = 2. When two samples had a higher consensus score in distinct interactions, they were more likely to be clustered together. (b) OS curves based on LUAD patients from the TCGA cohort for the two pyroptosis-related clusters. (c) Consensus clustering of differentially expressed genes in the TCGA cohort's two pyroptosis-related clusters.

We did a consensus clustering analysis of all the LUAD cases in the TCGA cohort, with a focus on all the 29 pyroptosis-related DEGs, in order to investigate the links between those expression patterns and LUAD subtypes. In order to see whether two groups can be successfully distinguished using the DEGs, we increased the clustering variable (*k*) from 2 to 10. As we predicted, at *k* = 2, the intragroup correlations were low, suggesting that the LUAD patients could be well split into two clusters (Figure 2(a)). A heatmap depicts the gene expression profile as well as clinical characteristics such as stage, grade, age, and gender, with grade indicating a difference between the two groups (Figure 2(c)). The OS time of the two clusters was also examined, and a substantial difference was discovered (*p*=0.031; Figure 2(b)).

### 3.3. Gene Signature Constructed from Pyroptosis-Related Clustering

**Figure 3:**
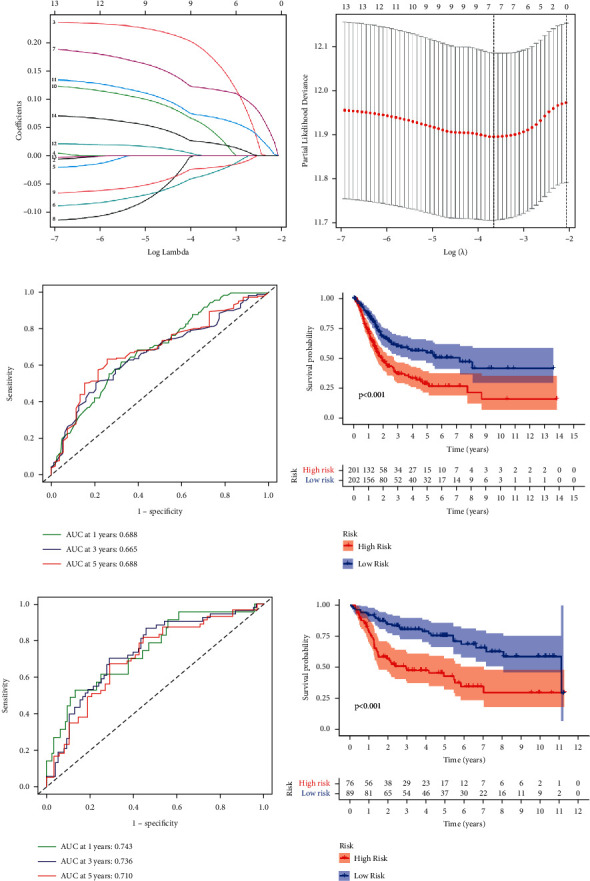
The development of a gene signature to predict patient OS based on clusters associated with pyroptosis. (a-b) Ten-fold cross-validation was used to determine the value of the super parameter in the TCGA LASSO-Cox model. (c) Time-dependent ROC analysis of the risk score in the TCGA cohort. (d) OS curves for the TCGA cohort's various risk score subgroups. (e) Time-dependent ROC analysis of the risk score in the GEO cohort. (f) OS curves for the GEO cohort's various risk score subgroups.

**Figure 4:**
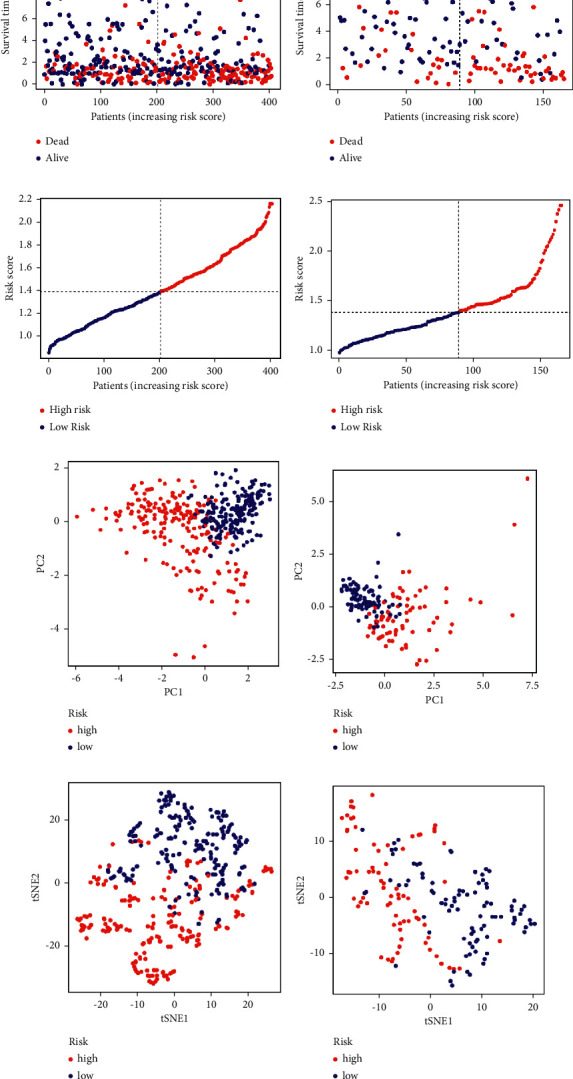
Prognostic study of the TCGA and GEO cohorts using the seven-gene signature model. Cohorts of the TCGA (a, c, e, and g), and the GEO cohort (b, d, f, and h). (a-b) The distribution of operating system status. (b-c) The risk scores' median value and dispersion. (f-e) Plot of principal component analysis. (g-h) Examination of the t-SNE coefficients.

On the basis of these findings, we created a gene signature that may be used in the treatment of LUAD and can be used to calculate an individual patient's LUAD score. The gene signature was also assessed in order to diagnose and treat each patient on an individual basis. First, 1458 DEGs with |log_2_FC| ≥ 1 and *p* < 0.05 were shown to be linked with the two clusters (Figure S1; Table S2). 13 genes were identified as independent prognostic signatures by univariate regression analysis (Table S3). Applying the LASSO-Cox regression model with a minimum of *λ*, 7 of the 13 DEGs were maintained in order to construct a model capable of quantifying each patient. We utilized these to create a signature that was associated with pyroptosis (Figures 3(a) and 3(b)). The next step was to try to evaluate the signature's worth by forecasting patient prognosis. In accordance with the median cut-off value, the patients were split into two groups (Figure 4(c)). Patients in various risk categories were dispersed in two directions according to PCA and t-SNE analyses (Figures 4(e) and 4(g)). Additionally, the scatter chart showed that individuals at high risk died sooner than those at low risk (Figure 4(a)). The Kaplan–Meier curve consistently indicated that high-risk patients had a substantially shorter OS than low-risk patients (Figure 3(d); *p* < 0.001). For examination of the prognostic model's survival prediction, time-dependent ROC curves were constructed, and the AUC reached 0.688 at one year, 0.665 at three years, and 0.688 at five years (Figure 3(c)).

### 3.4. Validation of the 7-Gene Signature in the GEO Cohort

Patients in the GEO cohort were also divided into high-risk and low-risk groups based on the median value from the TCGA cohort in order to assess the stability of the model built from the TCGA dataset. PCA and t-SNE analysis revealed a distinct distribution of patients in the two categories, similar to the TCGA cohort findings (Figures 4(f) and 4(h)). Additionally, individuals in the high-risk group died sooner (Figure 4(b)) and had a lower OS time when compared with those in the low-risk group (Figure 3(f)). Additionally, the AUC for the seven-gene signature was 0.743 at one year, 0.736 at three years, and 0.710 at five years (Figure 3(e)).

### 3.5. The 7-Gene Signature's Independent Prognostic Value

**Figure 5:**
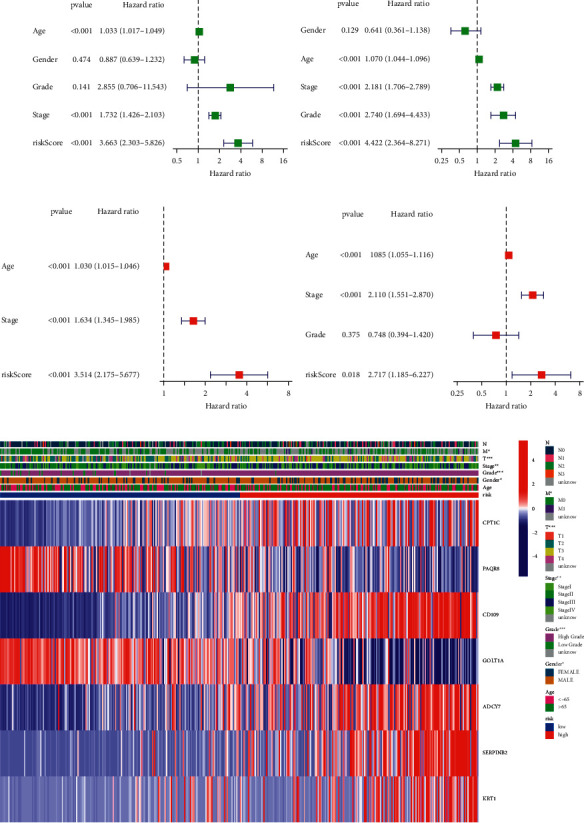
Prognostic accuracy of risk score and clinicopathological factors were compared. TCGA cohort (a, c). GEO cohort (b, d). (a, b) Univariate cox regression analysis was used to screen OS-related variables. (c, d) Multivariate cox regression analysis was used to screen OS-related variables. (e) Heatmap depicting the clinicopathological characteristics and gene expression variations between the high- and low-risk groups.

The Cox analyses of factors, both univariate and multivariate, were used to evaluate whether or not the risk score was an independent prognostic predictor for OS. Using univariate Cox analysis, it was discovered that risk ratings in both the TCGA and GEO cohorts were substantially associated with OS (TCGA cohort: HR = 3.663, 95%CI = 2.303 − 5.826, *p* < 0.001; GEO cohort: HR = 4.422, 95%CI = 2.364 − 8.271, *p* < 0.001) (Figures 5(a) and 5(b)). Multivariate Cox analysis showed that the risk score remained a significant predictor when additional confounding factors were taken into consideration. (TCGA cohort: HR = 2.717, 95%CI = 2.175 − 5.677, *p* < 0.001; GEO cohort: HR = 1.185 − 6.227, *p*=0.018) (Figures 5(c) and 5(d)). Furthermore, we created a heatmap of clinical characteristics for the TCGA cohort (Figure 5(e)) and discovered that gender, grade, stage, and so on were distributed differently across the low- and high-risk categories (*p* < 0.05).

### 3.6. Developing and Validating a Nomogram That Incorporates Clinical Features

**Figure 6:**
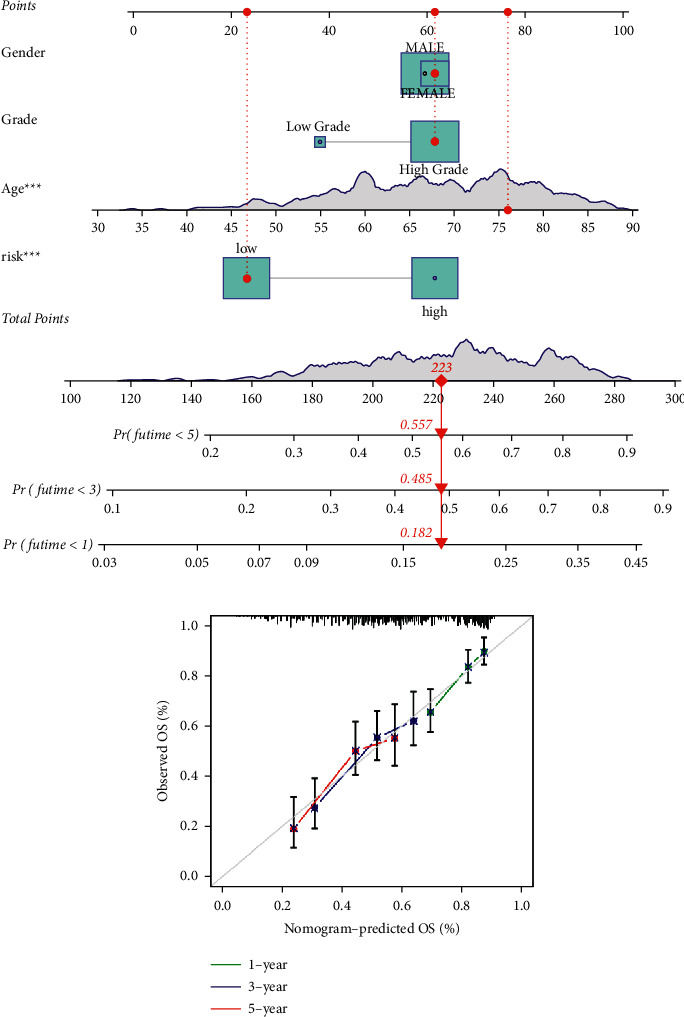
A prognostic signature-based nomogram for predicting 1-, 3-, and 5-year OS in LUAD patients. (a) A nomogram for predicting survival in the TCGA cohort. (b) Nomogram calibration plots for predicting OS at 1, 3, and 5 year in the TCGA cohort.

Because the risk score was strongly associated with high malignancy, we integrated clinical variables and created a nomogram in the TCGA cohort to expand the clinical applicability and use of the signature (Figure 6(a)). By combining the points for each prognostic criterion, each patient was given a total point value. Patients with a higher overall point had a poorer clinical outcome. Additionally, the calibration plot demonstrated that the nomogram operated in a manner consistent with an ideal model (Figure 6(b)).

### 3.7. Functional Analyses Based on the Risk Model

**Figure 7:**
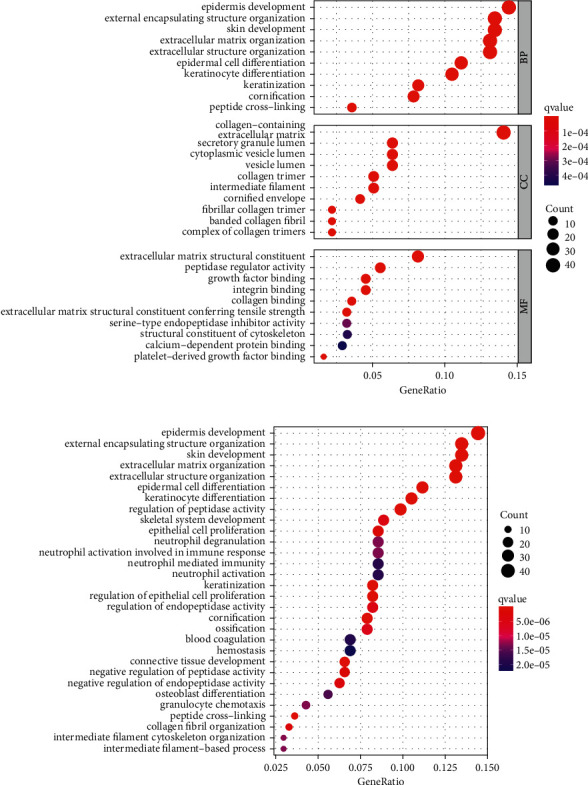
(a) GO and (b) KEGG analyses for differentially expressed genes among high and risk groups.

To investigate further the variations in gene functions and pathways across the risk model subgroups, we used the “limma” R package to extract DEGs using the FDR < 0.05 and |log_2_FC| ≥ 1 criterion. According to the TCGA cohort, a total of 317 DEGs were found between those who were at low and high risk of LUAD. As a result, 161 genes were upregulated, whereas 156 genes were downregulated in the high-risk group (Table S4). Based on these DEGs, GO and KEGG analyses were carried out. The DEGs were mostly associated with immunological and cell differentiation signaling pathways according to the findings (Figures 7(a) and 7(b)).

### 3.8. Comparative Analysis of Immunological Activation among Subgroups

**Figure 8:**
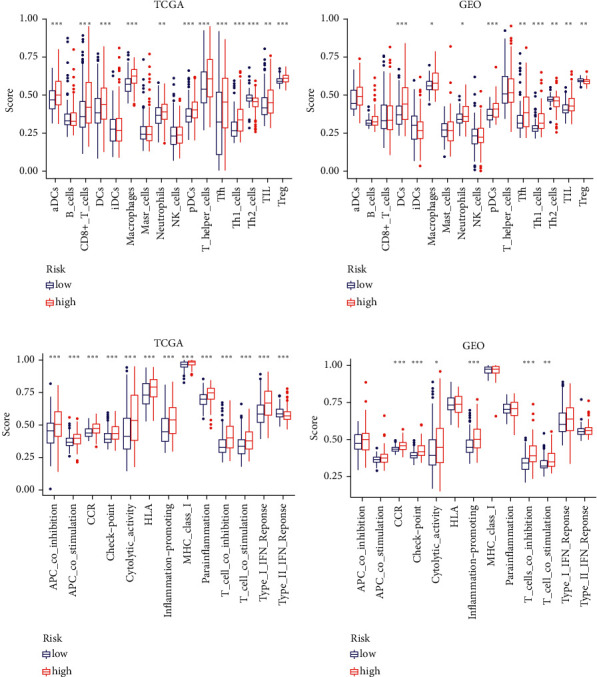
Immune status between two risk groups TCGA (a, c), GEO (b, d). (a-b) Boxplots were used to display the scores of 16 immune cells and (c-d) 13 immune-related functions.

On the basis of the functional analyses, we used the single-sample gene set enrichment analysis (ssGSEA) to compare the enrichment scores for 16 different types of immune cells, as well as the activity of 13 different immune-related pathways, between the low-and high-risk groups in both the TCGA and GEO cohorts. The high-risk subgroups in the TCGA cohort (Figure 8(a)) exhibited higher levels of immune cell infiltration, particularly CD8+ T cells, macrophages, and T helper cells (Tfh, Th1, and Th2 cells) than the low-risk category. Except for the Type II IFN response pathway, the other 12 immune pathways showed more activity in the high-risk group than in the low-risk group during immune function analysis (Figure 8(c)). Similar results were found when evaluating the immunological state of the GEO cohort (Figures 8(b) and 8(d)).

## 4. Discussion

Lung cancer is widely recognized to be the leading cause of mortality around the globe. Lung cancer patients with NSCLC, of whom roughly 50% have LUAD, make up almost 80 percent of all lung cancer patients [25]. Despite advances in treatment regimens, the survival rate for LUAD patients remains low. The high-level variability of LUAD, as well as the complicated etiologic variables, makes prognostic prediction difficult. As a result, the development of new prognostic models is critical.

Pyroptosis is a kind of programmed cell death that happens in cells attacked by pathogens, triggering the body's inflammatory response [15, 26]. Apoptosis may therefore be changed to pyroptosis in response to pathogen activation. Pyroptosis is involved in a variety of malignancies [27]. It inhibits tumor development in colorectal, liver, and skin cancers but has a bidirectional impact on breast cancer [28–31]. As a result, we cannot assess the predictive usefulness of LUAD only on the basis of the expression of various gasdermins. As a result, we investigated all pathways associated with pyroptosis and developed a predictive signature. Pyroptosis is now being explored for antitumor treatment, and our research suggests that pyroptosis in conjunction with immunotherapy may be a feasible therapeutic approach for patients with poor prognoses.

Classification of samples according to specified gene expression criteria is a well-established technique. On the basis of the expression of pyroptosis-related genes, we developed a subtyping approach for LUAD patients. We demonstrated a substantial association between the expression of these pyroptosis-related genes and various survival risks. Then, using the two clusters, we built a risk score model to quantify the prognostic risk. Our research established a solid case for LUAD clinical treatment. To begin, the risk score takes the patient's heterogeneity into consideration. Second, this score may be used to establish a connection between pyroptosis and prognosis. A high-risk score was associated with poor clinical characteristics and a shorter projected survival time. The results on TME cell infiltration showed that the risk score is critical for immunotherapy. Most active immune cell infiltration resulted in a superior response to immunotherapy in individuals with a high score. A predictive model for LUAD under hypoxia or modified m6A conditions was investigated as was the immunoscore. A major emphasis of our study has been on the factors that directly contribute to tumor cell death and change the tumor microenvironment. As a consequence, our method is more advantageous in terms of facilitating treatment.

Seven genes were included in our model (CPT1C, PAQR8, CD109, GOLT1A, ADCY7, SERPINB2, and KRT1). CPT1C is a member of the CPT1 family of enzymes that catalyzes the acylation of fatty acids and their entrance into the mitochondria for oxidation. CPT1C has a role in the regulation of energy balance, ceramide metabolism, and the hypothalamic control of food intake [32]. CPT1C levels were recently implicated in the poor prognosis and metastatic development of human malignancies, which are intimately linked to fatty acid absorption and metabolism. CD109 is a glycoprotein with a glycosylphosphatidylinositol (GPI) anchor that is overexpressed in squamous cell carcinomas of the lung, esophagus, uterus, and oral cavity. CD109 inhibits TGF-*β* signaling in keratinocytes via direct modulation of receptor activation [33]. According to Kazuhiro, miR-378a-3p regulates tamoxifen sensitivity in breast cancer MCF-7 cells via targeting GOLT1A [34]. According to Li et al., ADCY7 promotes the development of acute myeloid leukemia and that inhibiting ADCY7 may be a new approach for treating leukemia [35]. SERPINB2 is a stress protein because it is highly expressed in activated monocytes and macrophages, as well as differentiating keratinocytes [36]. According to Han et al., the transcript level of KRT1 may serve as a possible prognostic biomarker in patients with melanoma [37]. PAQR8, one of the sex steroid hormones, is supposed to be potential prognostic biomarker of endometrial cancer [38].

Our research sought to categorize patients with LUAD, discover DEGs, develop a predictive model, and establish a connection between pyroptosis and patient prognosis. There are limits to this study despite our attempts to verify it from many perspectives and using multiple databases. Further, in vitro and in vivo testing of our model is needed to get a deeper understanding of how the risk score and cell pyroptosis are related. These have not only exacerbated the difficulties but also provided us with optimism, which motivates us to continue digging.

## 5. Conclusion

In addition to providing some hints for future study on the mechanism of pyroptosis-related genes, our model may also offer additional resources for a better understanding of immune cell-specific genes implicated in cancer control.

## Data Availability

Publicly available datasets were used in this study. These data can be found in the gene expression omnibus (GEO) database and in the cancer genome atlas (TCGA) database.

## Abbreviations

DEGs: Differentially expressed genes
TCGA: The cancer genome atlas
GEO: Gene expression omnibus
GO: Gene ontology
BP: Biological processes
MF: Molecular function
CC: Cellular components
KEGG: Kyoto encyclopedia of genes and genomes
FDR: False discovery rate
ssGSEA: Single-sample gene set enrichment analysis (ssGSEA).

## Supplementary Materials

Supplementary Materials Supplementary File Table S1: 52 genes associated with pyroptosis from prior reviews. Supplementary File Table S2: 1458 DEGs associated with cluster 1 and cluster 2. Supplementary File Table S3: 13 genes identified by univariate regression. Supplementary File Table S4: 317 DEGs between low- and high-risk groups in TCGA cohort. Supplementary File Figure S1: An overview of the differential gene expression between the two pyroptosis-related clusters in TCGA cohort.
