# Supplementary material for: Identification of a Pyroptosis-Related Gene Signature for Prediction of Overall Survival in Lung Adenocarcinoma
Source: J Oncol. 2021 Sep 30;2021:6365459. doi: 10.1155/2021/6365459 (PMC8497135; doi:10.1155/2021/6365459)
Supplement: Supplementary Materials — Supplementary File Table S1: 52 genes associated with pyroptosis from prior reviews. Supplementary File Table S2: 1458 DEGs associated with cluster 1 and cluster 2. Supplementary File Table S3: 13 genes identified by univariate regression. Supplementary File Table S4: 317 DEGs between low- and high-risk groups in TCGA cohort. Supplementary File Figure S1: An overview of the differential gene expression between the two pyroptosis-related clusters in TCGA cohort. [file 6365459.f1.zip › 6365459.f1/TableS1.docx]

Table S1. 52 pyroptosis-related genes from prior reviews.

BAK1

BAX

CASP1

CASP3

CASP4

CASP5

CHMP2A

CHMP2B

CHMP3

CHMP4A

CHMP4B

CHMP4C

CHMP6

CHMP7

CYCS

ELANE

GSDMD

GSDME

GZMB

HMGB1

IL18

IL1A

IL1B

IRF1

IRF2

TP53

TP63

AIM2

CASP6

CASP8

CASP9

GPX4

GSDMA

GSDMB

GSDMC

IL6

NLRC4

NLRP1

NLRP2

NLRP3

NLRP6

NLRP7

NOD1

NOD2

PJVK

PLCG1

PRKACA

PYCARD

SCAF11

TIRAP

TNF

GZMA
