# Supplementary material for: Identification of a Pyroptosis-Related Gene Signature for Prediction of Overall Survival in Lung Adenocarcinoma
Source: J Oncol. 2021 Sep 30;2021:6365459. doi: 10.1155/2021/6365459 (PMC8497135; doi:10.1155/2021/6365459)
Supplement: Supplementary Materials — Supplementary File Table S1: 52 genes associated with pyroptosis from prior reviews. Supplementary File Table S2: 1458 DEGs associated with cluster 1 and cluster 2. Supplementary File Table S3: 13 genes identified by univariate regression. Supplementary File Table S4: 317 DEGs between low- and high-risk groups in TCGA cohort. Supplementary File Figure S1: An overview of the differential gene expression between the two pyroptosis-related clusters in TCGA cohort. [file 6365459.f1.zip › 6365459.f1/TableS2.docx]

Table S2. 1458 DEGs associated with cluster 1 and cluster 2.

| gene | cluster1 | cluster2 | logFC | pValue | fdr |
| --- | --- | --- | --- | --- | --- |
| IGKV1-17 | 26.85368 | 11.00694 | -1.28671 | 0.00037 | 0.000911 |
| ZNF823 | 3.178323 | 6.776397 | 1.092253 | 3.00E-21 | 2.03E-19 |
| CLDN3 | 8.162463 | 41.4937 | 2.345816 | 1.27E-11 | 1.26E-10 |
| TGFBI | 66.1313 | 27.64013 | -1.25857 | 1.78E-11 | 1.72E-10 |
| HLA-DQA1 | 23.7764 | 7.385484 | -1.68677 | 6.29E-18 | 2.21E-16 |
| IL15 | 1.041532 | 0.331695 | -1.65078 | 1.08E-36 | 8.81E-34 |
| CD48 | 5.072368 | 1.518227 | -1.74027 | 2.43E-13 | 3.32E-12 |
| QPRT | 5.353273 | 14.35792 | 1.423354 | 2.44E-15 | 4.77E-14 |
| DIRAS1 | 0.550976 | 1.210453 | 1.135485 | 0.001761 | 0.00369 |
| PSTPIP1 | 2.206153 | 0.868876 | -1.34431 | 2.76E-17 | 8.72E-16 |
| TXK | 0.413308 | 0.887861 | 1.103118 | 6.80E-10 | 4.99E-09 |
| GLIPR1 | 4.388141 | 2.078272 | -1.07823 | 3.23E-18 | 1.20E-16 |
| PARVG | 1.778907 | 0.805868 | -1.14238 | 3.07E-12 | 3.42E-11 |
| RHOD | 51.20027 | 24.81081 | -1.04518 | 9.73E-10 | 6.96E-09 |
| HLA-DOA | 10.7883 | 4.866354 | -1.14855 | 1.81E-12 | 2.09E-11 |
| FGFR4 | 0.745427 | 1.583495 | 1.086973 | 7.39E-05 | 0.000211 |
| CASP4 | 11.56432 | 5.69009 | -1.02316 | 9.62E-27 | 1.89E-24 |
| CAPN5 | 7.879773 | 17.91513 | 1.184953 | 7.59E-18 | 2.63E-16 |
| CD27 | 4.683225 | 1.555663 | -1.58997 | 3.27E-11 | 3.01E-10 |
| TGM1 | 10.14511 | 3.085348 | -1.71728 | 5.69E-09 | 3.49E-08 |
| IGLV2-14 | 90.74652 | 43.30261 | -1.06739 | 0.00064 | 0.001481 |
| LILRB3 | 0.768065 | 0.355268 | -1.11232 | 5.33E-13 | 6.80E-12 |
| TNFAIP8L2 | 4.564084 | 1.764647 | -1.37095 | 8.72E-17 | 2.43E-15 |
| CD209 | 3.575893 | 1.10946 | -1.68845 | 1.94E-15 | 3.92E-14 |
| HAPLN3 | 11.2236 | 3.879143 | -1.53273 | 5.13E-23 | 4.69E-21 |
| PLA2G2F | 8.112206 | 24.31752 | 1.58383 | 3.06E-12 | 3.41E-11 |
| PLAAT4 | 84.22361 | 24.74467 | -1.76711 | 3.16E-20 | 1.70E-18 |
| S100A7A | 4.45548 | 2.187095 | -1.02656 | 5.29E-08 | 2.76E-07 |
| GATA2 | 6.939423 | 18.25854 | 1.395684 | 4.05E-12 | 4.38E-11 |
| FGF12 | 0.448573 | 1.189322 | 1.406726 | 1.40E-06 | 5.63E-06 |
| NKG7 | 19.87465 | 3.424533 | -2.53695 | 3.25E-27 | 6.72E-25 |
| RHBG | 1.422504 | 6.23587 | 2.132158 | 4.64E-21 | 2.96E-19 |
| BEND5 | 1.636285 | 3.443614 | 1.073499 | 1.38E-16 | 3.62E-15 |
| MTUS2 | 0.336837 | 1.385784 | 2.04058 | 3.43E-11 | 3.13E-10 |
| IGHV1-46 | 21.99199 | 7.979183 | -1.46267 | 0.000101 | 0.000281 |
| ISG20 | 4.678171 | 1.403188 | -1.73724 | 1.68E-21 | 1.21E-19 |
| LINC01300 | 0.152435 | 0.783292 | 2.361356 | 2.02E-07 | 9.41E-07 |
| HMGCS2 | 27.09159 | 153.5769 | 2.503044 | 2.66E-17 | 8.44E-16 |
| LINC02195 | 2.605954 | 0.484591 | -2.42697 | 2.85E-24 | 3.44E-22 |
| THSD7A | 0.2618 | 0.708344 | 1.435984 | 6.36E-08 | 3.27E-07 |
| MMP13 | 30.13599 | 6.802177 | -2.14742 | 2.10E-07 | 9.77E-07 |
| LILRB1 | 1.564425 | 0.650637 | -1.26571 | 7.16E-17 | 2.02E-15 |
| MCOLN2 | 1.201068 | 0.459516 | -1.38613 | 1.39E-15 | 2.93E-14 |
| CD14 | 61.91404 | 22.36544 | -1.469 | 7.65E-18 | 2.65E-16 |
| IGHV4-28 | 5.864312 | 2.344353 | -1.32277 | 0.000636 | 0.001473 |
| VIP | 0.186808 | 0.774984 | 2.052608 | 0.002433 | 0.004923 |
| SERPINA1 | 27.56947 | 9.072127 | -1.60356 | 1.49E-15 | 3.12E-14 |
| GBP4 | 19.8994 | 5.490292 | -1.85777 | 9.99E-27 | 1.93E-24 |
| IGHV3-66 | 4.563318 | 1.887592 | -1.27354 | 0.006708 | 0.012196 |
| ACVR2B-AS1 | 0.588992 | 1.218702 | 1.049024 | 2.75E-18 | 1.05E-16 |
| MT-TV | 0.348233 | 1.063387 | 1.61054 | 6.90E-08 | 3.53E-07 |
| BANF1P2 | 0.388077 | 1.184357 | 1.609689 | 2.40E-06 | 9.25E-06 |
| CX3CL1 | 10.83033 | 4.89109 | -1.14685 | 0.009111 | 0.015998 |
| RAPSN | 0.220498 | 0.894883 | 2.020937 | 3.36E-10 | 2.60E-09 |
| CCL3 | 6.326782 | 1.729195 | -1.87137 | 4.38E-22 | 3.52E-20 |
| TRIM24 | 5.543035 | 12.36735 | 1.157789 | 4.57E-21 | 2.93E-19 |
| SLC4A4 | 0.519369 | 1.105589 | 1.089984 | 1.68E-05 | 5.44E-05 |
| CRTAC1 | 9.146145 | 22.05422 | 1.269819 | 1.25E-06 | 5.08E-06 |
| DBH-AS1 | 0.926484 | 0.376296 | -1.2999 | 1.50E-10 | 1.23E-09 |
| NCF1 | 1.518403 | 0.36442 | -2.05888 | 2.39E-17 | 7.62E-16 |
| BTNL9 | 0.712809 | 1.54564 | 1.116616 | 2.54E-12 | 2.87E-11 |
| CXCR2P1 | 1.312867 | 0.15922 | -3.04363 | 5.36E-18 | 1.91E-16 |
| DPYD | 6.463055 | 1.553552 | -2.05665 | 5.53E-38 | 5.50E-35 |
| SCUBE2 | 5.10166 | 16.0467 | 1.653238 | 4.05E-20 | 2.13E-18 |
| CARD16 | 6.056586 | 2.261952 | -1.42094 | 3.61E-31 | 1.52E-28 |
| EXOC3L4 | 1.655341 | 0.508323 | -1.70331 | 1.99E-06 | 7.74E-06 |
| TMEM97 | 23.6332 | 82.27199 | 1.799586 | 1.63E-26 | 3.03E-24 |
| SFN | 915.3506 | 404.6597 | -1.17762 | 1.84E-13 | 2.60E-12 |
| CELSR1 | 7.430805 | 3.53984 | -1.06983 | 2.86E-15 | 5.53E-14 |
| IGLV2-18 | 8.645388 | 2.046049 | -2.07909 | 0.010321 | 0.017853 |
| ADAM19 | 7.673366 | 3.673368 | -1.06276 | 7.38E-12 | 7.69E-11 |
| SPIRE2 | 2.601268 | 5.8869 | 1.178293 | 4.63E-15 | 8.59E-14 |
| LAMA3 | 18.36689 | 3.343406 | -2.45772 | 1.89E-18 | 7.51E-17 |
| SELPLG | 11.04038 | 4.117983 | -1.42278 | 4.89E-16 | 1.14E-14 |
| PPP1R3C | 5.6312 | 16.64516 | 1.563589 | 3.73E-18 | 1.36E-16 |
| LINC02672 | 1.178975 | 5.76832 | 2.290619 | 2.35E-10 | 1.86E-09 |
| PI3 | 740.1805 | 264.0042 | -1.48732 | 8.56E-14 | 1.28E-12 |
| THEMIS2 | 6.947801 | 3.214039 | -1.11217 | 9.60E-16 | 2.10E-14 |
| ATP6V0CP2 | 0.4743 | 1.625877 | 1.777345 | 4.59E-12 | 4.94E-11 |
| FOXO6 | 1.937316 | 6.216671 | 1.682083 | 2.22E-26 | 4.06E-24 |
| MIR7152 | 0.529669 | 2.047498 | 1.9507 | 3.67E-12 | 4.02E-11 |
| SLC13A3 | 0.603283 | 1.472069 | 1.286939 | 3.39E-12 | 3.75E-11 |
| CD38 | 2.096915 | 0.378342 | -2.47051 | 4.99E-23 | 4.60E-21 |
| CCER2 | 0.314713 | 8.970576 | 4.83309 | 1.81E-05 | 5.79E-05 |
| SFTPD | 2.825799 | 0.488145 | -2.53328 | 0.001558 | 0.003304 |
| SBK1 | 2.534885 | 7.711136 | 1.605023 | 6.68E-16 | 1.51E-14 |
| TRH | 0.012094 | 2.0268 | 7.388735 | 0.004208 | 0.008022 |
| CXCR6 | 2.135008 | 0.660375 | -1.69288 | 2.53E-18 | 9.68E-17 |
| PRKCQ | 1.668752 | 0.722148 | -1.2084 | 3.03E-19 | 1.38E-17 |
| PTPRZ1 | 2.010647 | 0.755618 | -1.41193 | 6.30E-09 | 3.82E-08 |
| LINC01711 | 0.899463 | 0.395742 | -1.18451 | 0.008299 | 0.014717 |
| LINC00967 | 0.557799 | 5.173535 | 3.213334 | 2.28E-18 | 8.84E-17 |
| SPRR2D | 29.29021 | 7.307487 | -2.00297 | 1.34E-05 | 4.40E-05 |
| H4C5 | 1.586597 | 4.243007 | 1.419151 | 0.001033 | 0.002286 |
| ADH6 | 0.293771 | 1.290303 | 2.134944 | 1.35E-12 | 1.60E-11 |
| LINC01977 | 0.266203 | 0.706309 | 1.407774 | 2.17E-15 | 4.32E-14 |
| SFTPA2 | 0.740227 | 1.818146 | 1.296428 | 2.85E-06 | 1.08E-05 |
| PPARG | 19.83458 | 44.7282 | 1.173167 | 1.85E-17 | 6.02E-16 |
| TNC | 28.21157 | 9.302567 | -1.60059 | 1.10E-11 | 1.11E-10 |
| BARX2 | 6.266254 | 2.578219 | -1.28123 | 3.71E-10 | 2.84E-09 |
| IGLV3-19 | 115.4426 | 32.50494 | -1.82844 | 1.62E-05 | 5.26E-05 |
| LAMP3 | 9.370875 | 3.940888 | -1.24966 | 5.10E-16 | 1.19E-14 |
| CTTNBP2 | 0.355381 | 1.291142 | 1.86121 | 6.95E-13 | 8.69E-12 |
| IGHGP | 46.62138 | 21.39365 | -1.12381 | 7.25E-05 | 0.000207 |
| ADAMDEC1 | 4.390312 | 1.108057 | -1.98629 | 2.23E-10 | 1.77E-09 |
| BNIPL | 3.868059 | 9.100902 | 1.2344 | 8.88E-10 | 6.41E-09 |
| CYP2C9 | 0.113658 | 1.17065 | 3.364543 | 1.93E-05 | 6.16E-05 |
| KRT16P6 | 10.43652 | 4.470411 | -1.22316 | 5.63E-10 | 4.17E-09 |
| NCMAP | 0.346893 | 0.891768 | 1.362179 | 1.05E-09 | 7.44E-09 |
| SGK2 | 0.672062 | 4.334398 | 2.689166 | 1.83E-21 | 1.28E-19 |
| RNF186 | 0.179721 | 1.816165 | 3.337066 | 7.17E-22 | 5.64E-20 |
| IGHG2 | 365.5306 | 174.08 | -1.07024 | 0.0002 | 0.000522 |
| FGFBP2 | 1.451137 | 0.225371 | -2.68681 | 0.000234 | 0.000602 |
| LINC01671 | 1.076881 | 4.080265 | 1.921803 | 7.79E-15 | 1.40E-13 |
| MIR4728 | 0.738158 | 2.790866 | 1.91871 | 4.85E-11 | 4.32E-10 |
| MMP10 | 19.62692 | 8.513414 | -1.20502 | 2.56E-05 | 8.00E-05 |
| PILRA | 3.674187 | 1.706637 | -1.10627 | 1.81E-09 | 1.24E-08 |
| 7SK | 3.575939 | 99.89823 | 4.804065 | 1.14E-07 | 5.58E-07 |
| IGLV1-40 | 114.4348 | 38.12775 | -1.58561 | 4.14E-06 | 1.53E-05 |
| FOXJ1 | 4.395064 | 10.44281 | 1.248554 | 8.78E-10 | 6.34E-09 |
| ANXA9 | 11.67317 | 25.33661 | 1.118028 | 1.62E-12 | 1.89E-11 |
| SPTSSB | 12.71142 | 33.12529 | 1.381808 | 3.72E-12 | 4.06E-11 |
| HES2 | 10.6641 | 3.510765 | -1.60291 | 3.93E-13 | 5.11E-12 |
| IFI27 | 124.9789 | 57.7619 | -1.11349 | 8.24E-18 | 2.84E-16 |
| CCSER1 | 0.428853 | 1.01154 | 1.237997 | 7.35E-15 | 1.32E-13 |
| KRT17 | 1519.041 | 574.6739 | -1.40235 | 1.38E-13 | 1.98E-12 |
| CD44 | 67.25065 | 23.69197 | -1.50515 | 8.68E-29 | 2.42E-26 |
| KLK12 | 1.084383 | 0.242171 | -2.16277 | 0.007863 | 0.014019 |
| IGKV3-20 | 288.7719 | 88.88367 | -1.69994 | 0.000201 | 0.000525 |
| IGLV1-36 | 11.37112 | 5.366176 | -1.08341 | 0.002538 | 0.005115 |
| TAF5LP1 | 1.162109 | 0.325193 | -1.83738 | 8.40E-17 | 2.34E-15 |
| FABP3 | 5.836166 | 51.96869 | 3.15455 | 7.15E-06 | 2.50E-05 |
| EVI2B | 6.308166 | 2.118711 | -1.57403 | 1.28E-16 | 3.41E-15 |
| SLAMF7 | 5.306632 | 1.855143 | -1.51627 | 1.94E-13 | 2.73E-12 |
| ACSM3 | 0.565161 | 1.803958 | 1.674431 | 9.02E-18 | 3.07E-16 |
| WASH6P | 5.579745 | 14.38072 | 1.365864 | 1.56E-07 | 7.41E-07 |
| LILRB5 | 1.01701 | 0.396618 | -1.35851 | 5.69E-09 | 3.49E-08 |
| S100A7 | 413.4008 | 181.5562 | -1.18713 | 1.91E-08 | 1.07E-07 |
| ERVH48-1 | 1.59907 | 6.176012 | 1.949442 | 9.31E-14 | 1.38E-12 |
| CYP4Z2P | 0.672008 | 1.382773 | 1.041013 | 2.15E-13 | 2.99E-12 |
| TRBV29-1 | 1.186243 | 0.374072 | -1.66501 | 1.51E-10 | 1.24E-09 |
| KCNF1 | 0.533217 | 2.10753 | 1.982758 | 2.15E-07 | 9.96E-07 |
| PSLNR | 0.673861 | 1.537299 | 1.189874 | 0.000564 | 0.001324 |
| CXCL1 | 44.58418 | 13.7092 | -1.70139 | 3.79E-16 | 9.05E-15 |
| IGKV5-2 | 5.134798 | 0.845809 | -2.6019 | 0.000511 | 0.001213 |
| PNCK | 3.3052 | 7.786455 | 1.236229 | 8.25E-06 | 2.85E-05 |
| REEP1 | 0.399762 | 0.946558 | 1.24355 | 0.00748 | 0.013403 |
| TMEM125 | 3.815764 | 8.925621 | 1.225981 | 6.73E-18 | 2.36E-16 |
| IGKV1-39 | 5.025916 | 0.549673 | -3.19274 | 0.008402 | 0.014882 |
| SASH3 | 7.989924 | 2.303854 | -1.79413 | 8.32E-19 | 3.48E-17 |
| XAF1 | 3.050618 | 1.509227 | -1.01529 | 5.83E-17 | 1.70E-15 |
| LINC02803 | 0.376932 | 0.791902 | 1.071019 | 4.40E-10 | 3.31E-09 |
| SENCR | 0.78362 | 0.361128 | -1.11765 | 1.61E-06 | 6.41E-06 |
| IGLV3-1 | 48.29627 | 16.40477 | -1.5578 | 2.88E-05 | 8.89E-05 |
| EBI3 | 3.172731 | 1.100645 | -1.52738 | 4.50E-09 | 2.82E-08 |
| SNX20 | 1.531665 | 0.535128 | -1.51715 | 3.35E-19 | 1.51E-17 |
| PARP9 | 13.43294 | 6.574497 | -1.03082 | 1.83E-29 | 5.53E-27 |
| GZMK | 2.871359 | 0.687036 | -2.06328 | 2.99E-11 | 2.76E-10 |
| SAMD11 | 0.801499 | 2.123797 | 1.405874 | 3.63E-12 | 3.98E-11 |
| IPO5P1 | 2.519756 | 5.879994 | 1.22253 | 6.71E-21 | 4.10E-19 |
| KRT14 | 844.492 | 139.4164 | -2.59868 | 9.08E-14 | 1.35E-12 |
| RAB11FIP4 | 2.730294 | 5.57396 | 1.029646 | 3.31E-17 | 1.03E-15 |
| NCF1C | 2.340979 | 0.571916 | -2.03324 | 2.58E-15 | 5.02E-14 |
| TLE6 | 0.863774 | 2.724864 | 1.657459 | 3.53E-16 | 8.50E-15 |
| BCAR3-AS1 | 0.336325 | 0.726737 | 1.11158 | 7.40E-13 | 9.21E-12 |
| PRF1 | 8.596998 | 2.510481 | -1.77587 | 1.99E-27 | 4.33E-25 |
| STMN2 | 0.210394 | 1.899474 | 3.174436 | 0.000863 | 0.001947 |
| IL7R | 5.92109 | 1.727119 | -1.7775 | 1.29E-18 | 5.27E-17 |
| LINC02889 | 2.901604 | 8.111258 | 1.483075 | 3.67E-06 | 1.37E-05 |
| IL2RB | 4.958589 | 1.719003 | -1.52836 | 2.79E-26 | 4.92E-24 |
| COL7A1 | 33.79092 | 15.26347 | -1.14655 | 1.31E-16 | 3.46E-15 |
| CXCL13 | 38.38556 | 6.245295 | -2.61972 | 1.51E-17 | 4.99E-16 |
| HSPA7 | 4.730786 | 2.002445 | -1.24032 | 2.91E-12 | 3.25E-11 |
| ATP6V1B1 | 1.117657 | 2.777542 | 1.313331 | 0.021519 | 0.034333 |
| AXL | 14.19607 | 6.638367 | -1.09659 | 1.56E-08 | 8.93E-08 |
| LPXN | 9.440864 | 4.258968 | -1.14842 | 6.25E-14 | 9.50E-13 |
| BIN2 | 2.400168 | 0.941929 | -1.34945 | 2.79E-16 | 6.87E-15 |
| KCTD14 | 1.870231 | 0.915627 | -1.03038 | 1.09E-06 | 4.48E-06 |
| ITGAL | 3.778276 | 1.122003 | -1.75165 | 1.01E-16 | 2.76E-15 |
| LINC02100 | 0.856914 | 0.22774 | -1.91176 | 9.41E-29 | 2.57E-26 |
| CDH23 | 0.564222 | 1.807813 | 1.67991 | 1.48E-19 | 7.12E-18 |
| GBP1 | 39.67198 | 8.707707 | -2.18776 | 2.21E-32 | 1.06E-29 |
| BSG-AS1 | 1.423027 | 3.283971 | 1.206478 | 6.86E-12 | 7.20E-11 |
| APOA1 | 0.383227 | 1.376944 | 1.845199 | 0.000318 | 0.000796 |
| PGM5-AS1 | 1.010696 | 2.863845 | 1.502604 | 0.008762 | 0.015446 |
| IFI30 | 0.806479 | 0.332475 | -1.27839 | 1.05E-20 | 6.20E-19 |
| FCER1G | 48.20694 | 16.08078 | -1.5839 | 3.17E-19 | 1.44E-17 |
| IGKV3D-11 | 2.254437 | 1.078121 | -1.06425 | 0.001666 | 0.00351 |
| HCP5 | 24.15868 | 6.596639 | -1.87274 | 1.19E-36 | 9.19E-34 |
| AMACR | 1.259538 | 3.365675 | 1.418002 | 5.53E-06 | 1.98E-05 |
| GNB4 | 4.557173 | 2.091434 | -1.12365 | 1.51E-18 | 6.05E-17 |
| SYT12 | 1.623494 | 0.464248 | -1.80613 | 3.89E-09 | 2.47E-08 |
| MT-TL1 | 2.423329 | 8.981641 | 1.889989 | 4.56E-14 | 7.09E-13 |
| CD69 | 4.056719 | 1.533151 | -1.40381 | 3.02E-11 | 2.78E-10 |
| SNORD69 | 1.577394 | 3.400677 | 1.108279 | 6.04E-14 | 9.21E-13 |
| STEAP1B | 1.429131 | 0.326356 | -2.13062 | 1.97E-11 | 1.89E-10 |
| PTAFR | 6.886321 | 2.84428 | -1.27567 | 3.92E-23 | 3.66E-21 |
| OSCAR | 3.378914 | 1.429616 | -1.24093 | 5.68E-13 | 7.21E-12 |
| VWA5B1 | 0.175679 | 0.917033 | 2.38403 | 1.51E-08 | 8.66E-08 |
| PRKAR1B-AS1 | 1.865512 | 0.688283 | -1.4385 | 4.21E-15 | 7.92E-14 |
| BHMT | 5.311964 | 24.12757 | 2.183366 | 6.56E-16 | 1.49E-14 |
| ECRG4 | 0.491363 | 1.046586 | 1.090829 | 0.010092 | 0.017495 |
| HLA-S | 1.710605 | 0.825311 | -1.0515 | 2.84E-10 | 2.21E-09 |
| STAT4 | 1.240766 | 0.380526 | -1.70516 | 8.74E-33 | 4.68E-30 |
| FGFBP1 | 58.51091 | 21.24313 | -1.46171 | 1.81E-11 | 1.75E-10 |
| MSMB | 12.82982 | 25.71718 | 1.003231 | 3.76E-06 | 1.40E-05 |
| SEMA4G | 0.893468 | 1.98712 | 1.153191 | 1.88E-12 | 2.17E-11 |
| MT-ND1 | 2701.391 | 5771.6 | 1.095269 | 1.90E-15 | 3.85E-14 |
| CCL4L2 | 4.547051 | 1.31037 | -1.79496 | 3.33E-18 | 1.23E-16 |
| MNX1-AS2 | 0.488753 | 1.0283 | 1.073084 | 2.53E-07 | 1.16E-06 |
| LINC01357 | 0.975674 | 0.479564 | -1.02467 | 2.50E-08 | 1.38E-07 |
| NOTUM | 0.36832 | 20.71493 | 5.813569 | 0.000595 | 0.001387 |
| SAA2 | 7.030636 | 0.93886 | -2.90467 | 1.71E-13 | 2.44E-12 |
| CAB39L | 6.754324 | 13.71212 | 1.021568 | 1.31E-16 | 3.46E-15 |
| SLC9A2 | 1.067337 | 4.694453 | 2.136941 | 1.42E-29 | 4.48E-27 |
| RAB38 | 22.37982 | 10.80306 | -1.05076 | 1.36E-06 | 5.49E-06 |
| GSTM1 | 30.80085 | 87.91305 | 1.513107 | 0.000227 | 0.000585 |
| CDH3 | 54.40454 | 14.6639 | -1.89146 | 5.12E-21 | 3.25E-19 |
| CASC15 | 0.367922 | 0.753027 | 1.033301 | 0.0008 | 0.001817 |
| KRT15 | 60.23186 | 16.37648 | -1.8789 | 2.55E-07 | 1.17E-06 |
| BNC1 | 5.234884 | 0.812042 | -2.68853 | 4.35E-16 | 1.02E-14 |
| IGHV1-2 | 33.87655 | 12.31453 | -1.45993 | 0.000415 | 0.001011 |
| PDIA2 | 0.271989 | 0.702799 | 1.369564 | 1.94E-07 | 9.10E-07 |
| IGKV3-11 | 141.4146 | 38.47801 | -1.87782 | 8.40E-06 | 2.90E-05 |
| RNF223 | 3.137804 | 7.812061 | 1.315948 | 4.21E-16 | 9.92E-15 |
| FER1L4 | 16.47364 | 39.35138 | 1.256255 | 2.15E-13 | 2.99E-12 |
| VASH2 | 0.412294 | 1.102567 | 1.41912 | 1.36E-11 | 1.35E-10 |
| CD53 | 22.77012 | 6.947982 | -1.71248 | 1.99E-17 | 6.42E-16 |
| FOXH1 | 0.291348 | 0.709483 | 1.284021 | 1.70E-13 | 2.42E-12 |
| HSD11B2 | 2.55273 | 7.277913 | 1.511484 | 1.32E-12 | 1.57E-11 |
| PSMB8-AS1 | 8.164297 | 3.301485 | -1.30621 | 7.11E-31 | 2.60E-28 |
| IGHV3-71 | 0.793021 | 0.353881 | -1.16409 | 0.0003 | 0.000753 |
| PCSK9 | 2.440435 | 1.079517 | -1.17675 | 1.36E-05 | 4.47E-05 |
| CHST15 | 5.342665 | 2.495233 | -1.09839 | 2.64E-13 | 3.57E-12 |
| HSPA6 | 14.30446 | 5.68005 | -1.33249 | 8.48E-05 | 0.000238 |
| AMOT | 0.950773 | 3.302577 | 1.796419 | 1.77E-15 | 3.63E-14 |
| C2 | 10.07729 | 3.857775 | -1.38527 | 4.93E-15 | 9.07E-14 |
| IGHV3-20 | 2.8551 | 1.189317 | -1.26341 | 0.009413 | 0.016457 |
| GPR183 | 7.776771 | 3.485605 | -1.15776 | 3.02E-13 | 4.05E-12 |
| CYP4F8 | 5.193885 | 14.50472 | 1.481636 | 2.61E-13 | 3.53E-12 |
| IL36G | 4.997008 | 0.719395 | -2.79621 | 9.88E-06 | 3.35E-05 |
| SIGLEC1 | 3.218949 | 1.026723 | -1.64854 | 2.18E-15 | 4.33E-14 |
| SCHLAP1 | 0.567285 | 1.134806 | 1.0003 | 4.03E-05 | 0.000121 |
| FMNL1 | 5.756323 | 2.583443 | -1.15585 | 3.53E-13 | 4.64E-12 |
| FUT9 | 0.287236 | 0.776331 | 1.434434 | 1.12E-13 | 1.64E-12 |
| DSC2 | 10.44604 | 5.048446 | -1.04905 | 1.12E-05 | 3.75E-05 |
| OVGP1 | 0.964697 | 3.805066 | 1.979773 | 2.36E-20 | 1.29E-18 |
| IGHG1 | 636.8654 | 255.4948 | -1.31769 | 5.33E-06 | 1.92E-05 |
| SPINK1 | 137.8034 | 509.4869 | 1.886433 | 9.45E-21 | 5.62E-19 |
| HLA-DPB1 | 102.5909 | 39.11924 | -1.39095 | 3.29E-17 | 1.03E-15 |
| PROM1 | 0.616553 | 2.115695 | 1.778834 | 2.27E-06 | 8.76E-06 |
| PCED1B-AS1 | 2.8728 | 0.919478 | -1.64357 | 4.66E-15 | 8.63E-14 |
| LINC01764 | 0.576359 | 2.535567 | 2.137268 | 2.27E-07 | 1.05E-06 |
| CD52 | 30.72139 | 8.834299 | -1.79806 | 8.67E-14 | 1.29E-12 |
| TNFSF10 | 81.70303 | 26.42781 | -1.62833 | 2.62E-30 | 9.11E-28 |
| CAPN9 | 0.683143 | 2.237886 | 1.711876 | 7.11E-14 | 1.07E-12 |
| FCMR | 4.492168 | 2.130344 | -1.07633 | 0.016626 | 0.027328 |
| ATP1A2 | 0.316597 | 1.354903 | 2.097471 | 0.000612 | 0.001425 |
| GATA3 | 51.54543 | 114.223 | 1.147937 | 2.37E-15 | 4.68E-14 |
| FAM171A2 | 1.206693 | 2.43724 | 1.01419 | 2.57E-07 | 1.18E-06 |
| SLA2 | 1.754058 | 0.425153 | -2.04464 | 2.35E-25 | 3.34E-23 |
| UPK3A | 18.11644 | 125.2861 | 2.789855 | 1.03E-26 | 1.96E-24 |
| WASIR2 | 0.386196 | 1.059916 | 1.456545 | 2.18E-17 | 6.99E-16 |
| C2orf72 | 0.279505 | 1.605911 | 2.522446 | 4.19E-06 | 1.54E-05 |
| XCR1 | 0.327483 | 0.741743 | 1.179501 | 0.006529 | 0.011905 |
| MAMDC2 | 1.669026 | 0.822702 | -1.02056 | 6.59E-06 | 2.32E-05 |
| LINC02894 | 0.990685 | 2.176347 | 1.135411 | 2.28E-21 | 1.57E-19 |
| RPS6KA6 | 0.622769 | 1.276592 | 1.035529 | 2.15E-11 | 2.04E-10 |
| CYP1A2 | 0.401402 | 11.17033 | 4.798481 | 2.03E-06 | 7.92E-06 |
| VIM-AS1 | 0.805335 | 0.3658 | -1.13853 | 4.37E-15 | 8.18E-14 |
| LINC02595 | 0.881765 | 0.377324 | -1.22459 | 2.50E-12 | 2.83E-11 |
| ODF3B | 12.00546 | 5.579242 | -1.10555 | 2.83E-24 | 3.44E-22 |
| LINC00941 | 1.662596 | 0.460258 | -1.85292 | 1.55E-21 | 1.13E-19 |
| MTRNR2L12 | 1.241605 | 2.629988 | 1.082849 | 6.54E-06 | 2.31E-05 |
| GPR171 | 1.300247 | 0.381354 | -1.76958 | 5.03E-18 | 1.81E-16 |
| MAL | 12.39296 | 52.34967 | 2.07866 | 8.60E-11 | 7.39E-10 |
| ST3GAL5-AS1 | 0.269467 | 0.722684 | 1.423255 | 6.38E-09 | 3.87E-08 |
| PM20D1 | 5.831023 | 48.20738 | 3.047433 | 2.16E-13 | 3.00E-12 |
| PIFO | 0.832803 | 2.217406 | 1.412827 | 3.27E-13 | 4.33E-12 |
| RPRM | 0.740917 | 2.156291 | 1.541168 | 7.03E-07 | 2.98E-06 |
| MAGI1 | 1.260762 | 2.979971 | 1.241002 | 3.28E-27 | 6.72E-25 |
| SPATA17 | 0.276738 | 0.74894 | 1.436327 | 4.93E-17 | 1.46E-15 |
| ARSJ | 1.555454 | 0.612171 | -1.34533 | 1.77E-11 | 1.71E-10 |
| CFAP251 | 1.097645 | 0.473322 | -1.21352 | 4.95E-08 | 2.59E-07 |
| SEMA3B-AS1 | 0.638622 | 1.374212 | 1.105569 | 6.44E-10 | 4.73E-09 |
| MIR559 | 0.341479 | 0.729802 | 1.09571 | 2.34E-06 | 9.02E-06 |
| FPR1 | 3.974695 | 1.675346 | -1.24639 | 5.10E-12 | 5.45E-11 |
| FGF8 | 0.042856 | 1.390652 | 5.02012 | 0.003674 | 0.007122 |
| MSI1 | 1.059538 | 2.581237 | 1.284627 | 3.80E-09 | 2.42E-08 |
| KLK3 | 0.206814 | 1.782259 | 3.107303 | 9.91E-06 | 3.36E-05 |
| B3GAT1-DT | 0.195076 | 1.232504 | 2.659486 | 3.46E-24 | 4.05E-22 |
| CD180 | 1.063049 | 0.391801 | -1.44001 | 4.97E-09 | 3.09E-08 |
| IGDCC3 | 0.144199 | 0.986352 | 2.774047 | 2.16E-20 | 1.20E-18 |
| EXT1 | 13.46857 | 6.656691 | -1.01672 | 4.14E-21 | 2.69E-19 |
| LINC02814 | 0.298599 | 1.221928 | 2.03288 | 2.59E-22 | 2.15E-20 |
| FMO8P | 0.143568 | 1.715195 | 3.578562 | 2.65E-12 | 2.99E-11 |
| MIR155HG | 1.372635 | 0.40675 | -1.75474 | 6.93E-18 | 2.42E-16 |
| S100A8 | 1024.05 | 303.6619 | -1.75375 | 4.72E-17 | 1.41E-15 |
| CST2 | 0.98595 | 2.663247 | 1.433599 | 0.005795 | 0.010704 |
| IL21R | 1.271611 | 0.346917 | -1.87399 | 1.24E-19 | 6.07E-18 |
| B2M | 1067.952 | 405.5875 | -1.39676 | 4.57E-38 | 4.90E-35 |
| ENTPD8 | 0.245676 | 1.92569 | 2.970549 | 0.006802 | 0.012351 |
| SECTM1 | 18.00142 | 7.265693 | -1.30894 | 2.58E-17 | 8.19E-16 |
| B4GALT6 | 0.782956 | 1.967279 | 1.329197 | 0.000105 | 0.000291 |
| KLRB1 | 1.819321 | 0.839814 | -1.11526 | 6.27E-11 | 5.51E-10 |
| ZBP1 | 1.603759 | 0.356557 | -2.16925 | 4.79E-19 | 2.08E-17 |
| TMCC3 | 3.420361 | 1.687451 | -1.0193 | 3.57E-13 | 4.69E-12 |
| IL16 | 1.222836 | 0.554531 | -1.14089 | 5.69E-09 | 3.49E-08 |
| IFIT2 | 9.260539 | 3.68608 | -1.32901 | 2.09E-15 | 4.19E-14 |
| LCK | 4.871518 | 1.643415 | -1.56767 | 9.58E-17 | 2.66E-15 |
| B3GALT5-AS1 | 0.339672 | 0.732442 | 1.108574 | 1.54E-09 | 1.06E-08 |
| PRR36 | 1.292463 | 5.43552 | 2.072295 | 6.98E-30 | 2.31E-27 |
| KRTAP5-9 | 1.266519 | 2.713684 | 1.099384 | 4.80E-08 | 2.52E-07 |
| UCA1 | 35.396 | 76.5329 | 1.112494 | 2.79E-05 | 8.64E-05 |
| GFI1 | 1.517495 | 0.6451 | -1.2341 | 5.67E-26 | 9.28E-24 |
| TOX3 | 2.106749 | 8.46017 | 2.005668 | 7.50E-24 | 8.22E-22 |
| NDNF | 0.317553 | 0.742853 | 1.22608 | 3.81E-05 | 0.000115 |
| POF1B | 6.301939 | 13.6759 | 1.117769 | 5.26E-14 | 8.09E-13 |
| GJB1 | 0.095111 | 1.870134 | 4.297384 | 1.52E-14 | 2.59E-13 |
| RNF157-AS1 | 0.360724 | 0.78904 | 1.129202 | 1.85E-14 | 3.09E-13 |
| CD3G | 1.260674 | 0.348362 | -1.85554 | 1.35E-20 | 7.87E-19 |
| NR1H4 | 0.334504 | 1.196532 | 1.838766 | 1.96E-09 | 1.32E-08 |
| RNASE2 | 1.69962 | 0.770054 | -1.14218 | 3.02E-13 | 4.05E-12 |
| HLA-DQA2 | 21.82787 | 8.791972 | -1.31191 | 2.39E-09 | 1.58E-08 |
| CYP2W1 | 1.288972 | 3.184234 | 1.304725 | 0.012968 | 0.021844 |
| FGFBP3 | 0.688334 | 1.417337 | 1.042002 | 1.97E-19 | 9.30E-18 |
| TSPEAR-AS1 | 0.243168 | 0.812781 | 1.740913 | 6.76E-05 | 0.000194 |
| CYP2B6 | 0.076816 | 0.990229 | 3.688281 | 0.011989 | 0.020386 |
| IGHV2-70 | 23.55727 | 4.226609 | -2.4786 | 9.85E-05 | 0.000274 |
| KRT78 | 1.226061 | 0.416815 | -1.55655 | 3.17E-07 | 1.43E-06 |
| CPT1C | 0.796282 | 1.663184 | 1.062595 | 2.27E-06 | 8.76E-06 |
| KRT17P3 | 1.938908 | 0.584342 | -1.73036 | 2.19E-09 | 1.47E-08 |
| TNNI3 | 0.533981 | 1.435884 | 1.42708 | 0.009717 | 0.016938 |
| NPTXR | 3.671892 | 8.638513 | 1.234259 | 7.04E-11 | 6.14E-10 |
| OSM | 2.544247 | 1.209311 | -1.07305 | 7.64E-13 | 9.48E-12 |
| CYP1A1 | 19.0315 | 81.33564 | 2.095498 | 3.33E-08 | 1.80E-07 |
| CD86 | 4.891311 | 1.635515 | -1.58048 | 4.99E-23 | 4.60E-21 |
| GBP5 | 11.29857 | 0.876227 | -3.68869 | 3.97E-30 | 1.35E-27 |
| HCST | 10.93108 | 3.915773 | -1.48107 | 6.54E-16 | 1.49E-14 |
| SOD2 | 25.64722 | 11.94079 | -1.1029 | 3.26E-16 | 7.90E-15 |
| CD4 | 13.12456 | 5.475361 | -1.26124 | 3.87E-15 | 7.34E-14 |
| IL33 | 9.978746 | 3.354836 | -1.57262 | 0.000107 | 0.000295 |
| MMP9 | 91.34596 | 16.07015 | -2.50696 | 7.28E-11 | 6.33E-10 |
| PDCD1 | 2.366227 | 0.46239 | -2.35541 | 4.32E-24 | 4.93E-22 |
| FAM3D | 5.363482 | 15.42995 | 1.524491 | 4.36E-11 | 3.91E-10 |
| ACOXL | 1.022259 | 2.818988 | 1.463417 | 1.51E-17 | 4.99E-16 |
| IL20RB | 18.63318 | 3.142225 | -2.56802 | 1.25E-16 | 3.35E-15 |
| SIGLEC12 | 1.114211 | 0.33847 | -1.71892 | 7.33E-13 | 9.13E-12 |
| DAPK1 | 7.020467 | 15.32525 | 1.126272 | 1.38E-16 | 3.62E-15 |
| CRYM | 0.664182 | 1.411485 | 1.087563 | 1.68E-07 | 7.96E-07 |
| MT-CYB | 3742.458 | 7490.501 | 1.001076 | 2.02E-16 | 5.13E-15 |
| ERP27 | 7.896384 | 21.56451 | 1.449395 | 7.70E-12 | 7.97E-11 |
| SAMD9 | 13.15424 | 3.236332 | -2.0231 | 1.40E-33 | 8.45E-31 |
| ZNF66 | 0.68832 | 1.882378 | 1.451406 | 1.42E-17 | 4.75E-16 |
| LINC02038 | 0.185609 | 0.937578 | 2.336668 | 6.65E-17 | 1.89E-15 |
| LAIR1 | 3.426114 | 1.312096 | -1.3847 | 3.04E-15 | 5.84E-14 |
| HS6ST3 | 0.615191 | 1.259743 | 1.034024 | 1.39E-12 | 1.64E-11 |
| PELATON | 1.798129 | 0.896373 | -1.00432 | 4.27E-13 | 5.52E-12 |
| CDA | 18.94401 | 5.463399 | -1.79387 | 4.50E-07 | 1.97E-06 |
| IGHV2-5 | 8.299905 | 2.429456 | -1.77246 | 0.000814 | 0.001847 |
| PACRG | 0.414219 | 0.957464 | 1.208822 | 4.45E-10 | 3.35E-09 |
| MMP7 | 45.28569 | 19.67882 | -1.20241 | 1.91E-05 | 6.12E-05 |
| AMTN | 3.536431 | 0.402436 | -3.13546 | 5.29E-06 | 1.90E-05 |
| CAPN6 | 0.421443 | 1.675362 | 1.991064 | 0.004568 | 0.008636 |
| IGHV4-31 | 17.72885 | 8.033495 | -1.142 | 0.000111 | 0.000306 |
| ZNF91 | 1.609015 | 3.252321 | 1.015292 | 2.63E-15 | 5.11E-14 |
| UCN2 | 3.452959 | 1.704653 | -1.01836 | 3.42E-14 | 5.43E-13 |
| TESC | 6.169852 | 21.06973 | 1.771864 | 3.16E-14 | 5.05E-13 |
| MTATP8P2 | 2.859967 | 23.0438 | 3.010308 | 0.000618 | 0.001436 |
| MT1L | 10.30393 | 4.360234 | -1.24072 | 1.34E-08 | 7.76E-08 |
| LIPC | 0.582907 | 1.516938 | 1.379824 | 3.01E-05 | 9.24E-05 |
| ZNF737 | 2.508958 | 7.229349 | 1.526777 | 2.80E-14 | 4.51E-13 |
| RNF217 | 2.101522 | 1.025802 | -1.03468 | 4.41E-18 | 1.59E-16 |
| SYN2 | 0.257998 | 1.117757 | 2.115176 | 2.98E-07 | 1.35E-06 |
| PLA2G2D | 2.496879 | 0.710108 | -1.81402 | 0.000431 | 0.001046 |
| CCR5 | 2.80157 | 0.745981 | -1.90902 | 3.18E-20 | 1.70E-18 |
| LINP1 | 2.49231 | 1.215354 | -1.03611 | 1.79E-06 | 7.06E-06 |
| IGHV3-21 | 41.29473 | 13.86243 | -1.57478 | 1.16E-06 | 4.74E-06 |
| ACAP1 | 2.679786 | 1.053527 | -1.34689 | 1.32E-13 | 1.91E-12 |
| CRISP3 | 1.222719 | 8.765527 | 2.841748 | 1.35E-08 | 7.82E-08 |
| TRBV28 | 7.740887 | 2.182455 | -1.82655 | 2.39E-17 | 7.62E-16 |
| GABRD | 0.702275 | 1.532121 | 1.125421 | 5.84E-10 | 4.31E-09 |
| RGS1 | 17.931 | 7.75449 | -1.20935 | 3.68E-14 | 5.81E-13 |
| RASAL3 | 3.330994 | 1.290627 | -1.36788 | 3.16E-14 | 5.05E-13 |
| ICA1 | 5.722681 | 11.59067 | 1.018201 | 4.38E-21 | 2.82E-19 |
| TASL | 0.970632 | 0.375359 | -1.37065 | 2.14E-14 | 3.52E-13 |
| HSD3B1 | 0.839202 | 2.441447 | 1.540647 | 1.44E-06 | 5.81E-06 |
| KLK10 | 11.51712 | 3.166443 | -1.86285 | 1.04E-12 | 1.26E-11 |
| KRT77 | 1.018375 | 0.372974 | -1.44912 | 0.00043 | 0.001044 |
| TMPRSS11A | 1.438298 | 0.279792 | -2.36194 | 5.21E-05 | 0.000153 |
| SOX7 | 4.89265 | 1.860866 | -1.39464 | 4.79E-13 | 6.15E-12 |
| PDCD1LG2 | 3.556548 | 0.670208 | -2.4078 | 8.42E-22 | 6.55E-20 |
| TRNP1 | 9.239232 | 20.4285 | 1.144739 | 1.01E-10 | 8.56E-10 |
| LRTM1 | 0.094807 | 0.882247 | 3.218122 | 3.01E-11 | 2.77E-10 |
| TRPM2-AS | 1.350538 | 2.735877 | 1.018469 | 9.10E-07 | 3.78E-06 |
| IGLV5-45 | 17.56283 | 3.176318 | -2.4671 | 3.99E-05 | 0.000119 |
| VNN2 | 1.306928 | 0.450039 | -1.53806 | 8.73E-10 | 6.32E-09 |
| CCL20 | 19.80664 | 5.745303 | -1.78553 | 1.74E-11 | 1.69E-10 |
| IGKV1D-43 | 0.993004 | 0.333691 | -1.57329 | 0.00713 | 0.012864 |
| ZNF888 | 2.342672 | 4.922867 | 1.071344 | 2.42E-13 | 3.30E-12 |
| SEMA5A | 4.677663 | 12.3486 | 1.400488 | 3.01E-16 | 7.39E-15 |
| CD3D | 11.74089 | 3.740537 | -1.65022 | 4.65E-19 | 2.03E-17 |
| ARHGAP25 | 2.804077 | 1.35125 | -1.05323 | 3.65E-12 | 4.00E-11 |
| DLL1 | 5.464809 | 2.667873 | -1.03448 | 0.000338 | 0.000842 |
| DDAH1 | 4.742485 | 10.45588 | 1.1406 | 1.07E-17 | 3.62E-16 |
| STAT1 | 86.64402 | 26.99003 | -1.68267 | 2.73E-36 | 2.00E-33 |
| S100A2 | 862.1438 | 266.9958 | -1.69111 | 7.25E-20 | 3.68E-18 |
| HIKESHIP1 | 0.317279 | 1.191313 | 1.908729 | 4.58E-15 | 8.50E-14 |
| IGKV2D-29 | 15.20448 | 3.527845 | -2.10764 | 0.000593 | 0.001383 |
| TMEM178A | 0.570894 | 1.426418 | 1.321101 | 1.90E-11 | 1.82E-10 |
| DSC3 | 35.57731 | 12.70918 | -1.48509 | 8.27E-15 | 1.48E-13 |
| CALHM6 | 12.69239 | 3.623566 | -1.80848 | 3.54E-21 | 2.34E-19 |
| CSF2 | 2.556496 | 0.841647 | -1.60288 | 7.56E-12 | 7.84E-11 |
| RASSF10 | 3.590091 | 1.794267 | -1.00063 | 1.20E-07 | 5.86E-07 |
| PRSS2 | 8.581082 | 53.36991 | 2.636795 | 0.004148 | 0.007924 |
| LAMB3 | 119.5618 | 44.60576 | -1.42245 | 2.16E-19 | 1.01E-17 |
| HCK | 7.546793 | 3.39742 | -1.15142 | 6.83E-18 | 2.39E-16 |
| FAM78A | 1.800107 | 0.880924 | -1.03099 | 4.00E-12 | 4.34E-11 |
| GPR65 | 0.841399 | 0.304435 | -1.46666 | 8.56E-18 | 2.93E-16 |
| HOXD11 | 2.028777 | 0.888408 | -1.19132 | 1.46E-12 | 1.72E-11 |
| MMP12 | 32.72237 | 14.41551 | -1.18266 | 1.55E-15 | 3.22E-14 |
| CYP3A5 | 2.495029 | 5.575786 | 1.160119 | 1.38E-07 | 6.65E-07 |
| SPRR2F | 4.187241 | 0.738723 | -2.5029 | 8.65E-05 | 0.000243 |
| DAB1 | 0.291588 | 1.684501 | 2.53032 | 3.82E-21 | 2.51E-19 |
| ACOX1 | 13.20992 | 29.71863 | 1.169746 | 1.29E-21 | 9.59E-20 |
| TRAF3IP3 | 1.135189 | 0.441671 | -1.36189 | 2.77E-18 | 1.05E-16 |
| TUBA5P | 2.075458 | 5.59372 | 1.430379 | 4.14E-24 | 4.76E-22 |
| LINC02866 | 0.531386 | 1.139434 | 1.100486 | 8.02E-05 | 0.000227 |
| CILP2 | 1.556086 | 3.160769 | 1.022354 | 4.29E-10 | 3.24E-09 |
| GBP6 | 5.89015 | 1.879639 | -1.64785 | 1.58E-12 | 1.85E-11 |
| SERPINB7 | 4.119646 | 1.040361 | -1.98544 | 2.13E-13 | 2.97E-12 |
| TRAC | 16.47444 | 4.799769 | -1.77919 | 2.37E-19 | 1.10E-17 |
| GDPD3 | 9.706457 | 30.24503 | 1.639681 | 1.99E-22 | 1.69E-20 |
| CASC22 | 0.110081 | 1.790222 | 4.023504 | 7.34E-14 | 1.10E-12 |
| MIR8071-1 | 6.255445 | 0.90183 | -2.79419 | 0.025459 | 0.039896 |
| MTCO1P40 | 8.721885 | 18.69563 | 1.099989 | 3.10E-07 | 1.40E-06 |
| IGKV2-28 | 1.429686 | 0.388332 | -1.88034 | 0.008494 | 0.015017 |
| RASL10A | 0.456555 | 1.506068 | 1.721926 | 1.55E-17 | 5.09E-16 |
| HLA-L | 2.404854 | 1.090027 | -1.14159 | 7.20E-12 | 7.52E-11 |
| MISP3 | 2.606744 | 5.719222 | 1.13357 | 5.88E-17 | 1.70E-15 |
| IL32 | 31.24687 | 11.23766 | -1.47537 | 1.62E-17 | 5.30E-16 |
| SMIM6 | 0.815837 | 2.11978 | 1.377561 | 4.87E-15 | 8.98E-14 |
| CTLA4 | 2.55568 | 0.726912 | -1.81386 | 2.78E-23 | 2.76E-21 |
| RALBP1 | 30.53043 | 64.13456 | 1.070854 | 1.11E-14 | 1.92E-13 |
| TRBV7-9 | 1.967937 | 0.477064 | -2.04443 | 6.76E-14 | 1.02E-12 |
| TGM2 | 62.77237 | 30.47245 | -1.04262 | 7.67E-11 | 6.65E-10 |
| INPP5J | 0.916717 | 1.882336 | 1.037975 | 4.93E-06 | 1.79E-05 |
| SLC22A3 | 2.156692 | 0.812679 | -1.40806 | 2.49E-09 | 1.65E-08 |
| PSMB8 | 89.62036 | 35.90977 | -1.31945 | 9.70E-39 | 1.12E-35 |
| SPNS2 | 4.249045 | 10.63815 | 1.324036 | 3.75E-09 | 2.39E-08 |
| GGTA1 | 1.336982 | 0.667792 | -1.00151 | 3.48E-07 | 1.56E-06 |
| IFI35 | 40.20841 | 18.21099 | -1.14269 | 1.76E-25 | 2.57E-23 |
| SLC29A3 | 7.190792 | 15.03342 | 1.06395 | 1.55E-19 | 7.42E-18 |
| EVX1 | 1.367584 | 3.660599 | 1.420451 | 6.11E-15 | 1.11E-13 |
| GPLD1 | 0.292528 | 0.91574 | 1.646365 | 6.22E-16 | 1.42E-14 |
| XDH | 4.354195 | 1.460884 | -1.57556 | 1.44E-16 | 3.76E-15 |
| KL | 0.401901 | 1.082344 | 1.429247 | 3.00E-05 | 9.22E-05 |
| AATBC | 3.446967 | 7.029896 | 1.028176 | 5.44E-07 | 2.35E-06 |
| C12orf54 | 1.067848 | 0.415402 | -1.36213 | 4.08E-09 | 2.57E-08 |
| ADORA2B | 6.208623 | 2.949061 | -1.07402 | 9.79E-17 | 2.70E-15 |
| MAGIX | 1.350852 | 3.388926 | 1.326959 | 3.15E-16 | 7.66E-15 |
| VCX3A | 0.471288 | 1.170661 | 1.312643 | 0.001016 | 0.002252 |
| ACSF2 | 9.167291 | 19.95427 | 1.12213 | 1.80E-20 | 1.01E-18 |
| IGLC6 | 1.277347 | 0.565822 | -1.17473 | 0.001375 | 0.00295 |
| IRF8 | 3.68324 | 1.529489 | -1.26793 | 7.88E-10 | 5.74E-09 |
| TREM1 | 2.632714 | 1.032091 | -1.35098 | 9.61E-15 | 1.69E-13 |
| IL10RA | 4.154954 | 1.464048 | -1.50487 | 1.39E-14 | 2.38E-13 |
| LY6E | 227.3581 | 107.9426 | -1.0747 | 4.15E-18 | 1.50E-16 |
| ASRGL1 | 0.74746 | 1.889209 | 1.337713 | 7.91E-07 | 3.32E-06 |
| KRTAP5-10 | 0.367324 | 0.788765 | 1.102541 | 9.74E-11 | 8.28E-10 |
| RNASE6 | 12.82716 | 5.182723 | -1.30742 | 3.98E-13 | 5.17E-12 |
| HLA-DQB1 | 44.14161 | 12.09882 | -1.86727 | 2.69E-20 | 1.46E-18 |
| IL12RB1 | 2.047702 | 0.455859 | -2.16735 | 5.92E-27 | 1.18E-24 |
| MEX3A | 4.581475 | 10.5217 | 1.199484 | 1.24E-11 | 1.24E-10 |
| RASL11B | 1.757903 | 4.747593 | 1.43334 | 2.78E-13 | 3.74E-12 |
| ZNF423 | 0.429953 | 0.973661 | 1.179239 | 1.19E-10 | 9.95E-10 |
| IGHV3-41 | 0.950266 | 0.257661 | -1.88286 | 0.015363 | 0.025493 |
| CCL8 | 5.673046 | 1.160808 | -2.28899 | 5.27E-11 | 4.68E-10 |
| SEMA6A | 2.770562 | 5.620972 | 1.020641 | 6.02E-11 | 5.31E-10 |
| PI4KAP1 | 1.540686 | 3.762014 | 1.287932 | 1.50E-10 | 1.23E-09 |
| DNALI1 | 0.868294 | 2.110232 | 1.281146 | 1.47E-07 | 7.02E-07 |
| OGDHL | 0.399354 | 0.90275 | 1.176657 | 0.00214 | 0.004395 |
| NCF2 | 7.84427 | 2.8344 | -1.4686 | 9.55E-22 | 7.30E-20 |
| SNORD93 | 0.862644 | 0.368433 | -1.22736 | 6.30E-09 | 3.82E-08 |
| LINC01116 | 3.263809 | 1.447168 | -1.17332 | 1.22E-13 | 1.78E-12 |
| LINC00709 | 0.170239 | 0.892662 | 2.39055 | 1.79E-10 | 1.44E-09 |
| ADRA2C | 1.194097 | 3.266231 | 1.451707 | 0.000153 | 0.000408 |
| VGLL1 | 26.61623 | 63.37006 | 1.251495 | 1.71E-15 | 3.53E-14 |
| ONECUT2 | 0.261809 | 0.920642 | 1.814125 | 4.86E-07 | 2.12E-06 |
| WFDC21P | 12.99511 | 54.35068 | 2.064329 | 3.70E-05 | 0.000111 |
| TM4SF19 | 1.557093 | 0.484013 | -1.68574 | 4.24E-10 | 3.21E-09 |
| HERC6 | 7.776522 | 3.235756 | -1.26502 | 1.45E-23 | 1.54E-21 |
| DNASE1L3 | 1.634247 | 0.483073 | -1.75831 | 0.000244 | 0.000626 |
| SNORD15B | 0.884896 | 1.870524 | 1.079863 | 0.00842 | 0.014912 |
| KIF5C | 0.85967 | 2.362566 | 1.4585 | 2.15E-15 | 4.28E-14 |
| SLC7A11 | 5.784766 | 1.418263 | -2.02813 | 1.35E-08 | 7.83E-08 |
| PLAT | 74.54265 | 35.43313 | -1.07297 | 9.36E-06 | 3.19E-05 |
| TM7SF2 | 7.715677 | 19.34643 | 1.326202 | 2.42E-23 | 2.44E-21 |
| MYCL | 13.43221 | 41.27471 | 1.619561 | 3.91E-21 | 2.55E-19 |
| IGHM | 177.6087 | 32.98786 | -2.4287 | 9.36E-06 | 3.19E-05 |
| LINC01983 | 0.408962 | 1.152154 | 1.494296 | 1.07E-10 | 9.05E-10 |
| CD74 | 877.582 | 286.9364 | -1.6128 | 3.47E-24 | 4.05E-22 |
| ABI3 | 5.896033 | 2.580436 | -1.19213 | 1.06E-14 | 1.84E-13 |
| SLC30A2 | 1.883365 | 8.284792 | 2.137153 | 7.36E-28 | 1.77E-25 |
| FCGR2C | 0.830237 | 0.406474 | -1.03036 | 2.12E-11 | 2.01E-10 |
| ARHGAP22 | 1.14257 | 0.556249 | -1.03848 | 4.24E-10 | 3.21E-09 |
| EVX1-AS | 0.421024 | 1.758854 | 2.062661 | 2.05E-12 | 2.35E-11 |
| CXCL10 | 115.2007 | 11.4351 | -3.33261 | 2.32E-27 | 4.93E-25 |
| FAHD2CP | 1.807525 | 4.463796 | 1.304255 | 5.51E-14 | 8.45E-13 |
| LY86 | 4.758133 | 2.193872 | -1.11692 | 2.15E-10 | 1.71E-09 |
| IGSF6 | 3.193315 | 1.35965 | -1.23182 | 4.01E-16 | 9.52E-15 |
| DEFB126 | 0.407555 | 1.070491 | 1.393207 | 6.54E-08 | 3.36E-07 |
| P2RY10 | 0.928065 | 0.261542 | -1.82718 | 1.92E-15 | 3.89E-14 |
| FABP5P7 | 5.323952 | 2.587688 | -1.04083 | 0.026079 | 0.040708 |
| PDZK1IP1 | 44.10974 | 17.50281 | -1.33351 | 8.90E-14 | 1.32E-12 |
| FARP1-AS1 | 0.472693 | 0.968474 | 1.03481 | 8.72E-10 | 6.31E-09 |
| CYBB | 12.55775 | 3.770447 | -1.73577 | 5.57E-18 | 1.97E-16 |
| PWWP3B | 0.506846 | 1.361687 | 1.425776 | 3.83E-06 | 1.42E-05 |
| IGHV3-11 | 43.43907 | 11.17956 | -1.95813 | 0.00011 | 0.000301 |
| ITGA6 | 48.09979 | 22.48027 | -1.09737 | 1.53E-11 | 1.50E-10 |
| LINC00482 | 0.604114 | 2.044276 | 1.758699 | 1.25E-08 | 7.29E-08 |
| DHRS2 | 77.3571 | 191.1428 | 1.305045 | 2.57E-14 | 4.16E-13 |
| TPSP2 | 1.728725 | 5.897104 | 1.770298 | 2.52E-07 | 1.15E-06 |
| IGHV3-23 | 138.0479 | 39.64293 | -1.80003 | 3.49E-05 | 0.000106 |
| TMEM145 | 0.318773 | 0.86637 | 1.442455 | 3.52E-08 | 1.89E-07 |
| KLK8 | 4.834049 | 1.486252 | -1.70155 | 5.07E-06 | 1.83E-05 |
| ABCD3 | 7.894287 | 16.23237 | 1.039993 | 1.83E-22 | 1.57E-20 |
| THBS4 | 0.708047 | 1.881327 | 1.409835 | 0.000173 | 0.000457 |
| APOL3 | 9.923745 | 3.19535 | -1.63491 | 4.89E-33 | 2.72E-30 |
| CEBPA-DT | 1.654448 | 3.566562 | 1.108184 | 3.11E-18 | 1.16E-16 |
| AGT | 1.671202 | 3.901199 | 1.223031 | 2.07E-06 | 8.06E-06 |
| DLX6 | 0.57658 | 1.503837 | 1.383056 | 4.75E-06 | 1.72E-05 |
| FCGR1A | 2.111212 | 0.725318 | -1.54139 | 1.75E-15 | 3.59E-14 |
| PAQR8 | 2.656089 | 6.085323 | 1.19603 | 4.12E-12 | 4.46E-11 |
| S100A9 | 3539.715 | 1327.769 | -1.41463 | 1.86E-14 | 3.10E-13 |
| HSPD1P6 | 0.217247 | 1.489454 | 2.777374 | 6.93E-08 | 3.55E-07 |
| CSF1R | 12.71896 | 4.709048 | -1.43347 | 2.48E-16 | 6.19E-15 |
| BMP4 | 2.689824 | 7.775249 | 1.531377 | 0.001075 | 0.002371 |
| IGKV3D-15 | 4.930623 | 1.240208 | -1.99119 | 0.00216 | 0.00443 |
| LAPTM5 | 76.83053 | 28.35385 | -1.43814 | 3.40E-21 | 2.27E-19 |
| RAC2 | 32.37371 | 12.44088 | -1.37973 | 7.26E-26 | 1.17E-23 |
| IGLVI-70 | 1.435094 | 0.225247 | -2.67157 | 0.001708 | 0.003587 |
| SMIM24 | 0.461407 | 1.519257 | 1.719255 | 5.66E-08 | 2.94E-07 |
| INA | 8.02284 | 19.31686 | 1.267676 | 2.02E-10 | 1.62E-09 |
| SULF2 | 29.20047 | 10.322 | -1.50027 | 1.00E-12 | 1.22E-11 |
| IFI44L | 7.363209 | 2.762019 | -1.41461 | 2.93E-23 | 2.85E-21 |
| SERPINB9 | 9.533049 | 4.664666 | -1.03116 | 5.84E-16 | 1.34E-14 |
| ITGB2 | 15.27215 | 5.017247 | -1.60593 | 1.54E-18 | 6.18E-17 |
| FAS | 5.899249 | 2.737405 | -1.10772 | 5.57E-26 | 9.22E-24 |
| TFPI | 4.29244 | 2.092278 | -1.03672 | 3.24E-09 | 2.09E-08 |
| ALB | 0.920902 | 4.690794 | 2.348713 | 0.00053 | 0.001253 |
| NT5E | 12.89134 | 4.917239 | -1.39048 | 2.53E-11 | 2.35E-10 |
| TDH | 0.442858 | 1.097581 | 1.30941 | 2.00E-12 | 2.29E-11 |
| SCARNA7 | 0.352713 | 3.692459 | 3.388015 | 2.08E-07 | 9.69E-07 |
| GAB3 | 0.767206 | 0.356427 | -1.10601 | 1.86E-14 | 3.10E-13 |
| CD109 | 7.633865 | 2.46217 | -1.63248 | 1.90E-15 | 3.85E-14 |
| IL12RB2 | 1.125719 | 0.212548 | -2.40499 | 3.92E-20 | 2.08E-18 |
| TRIM16L | 4.802401 | 2.279555 | -1.075 | 2.18E-06 | 8.45E-06 |
| TAC1 | 0.344757 | 0.884582 | 1.359417 | 7.86E-05 | 0.000223 |
| IRF1-AS1 | 0.909458 | 0.444371 | -1.03324 | 1.19E-19 | 5.81E-18 |
| SEPTIN3 | 0.958385 | 2.494139 | 1.379865 | 4.87E-09 | 3.03E-08 |
| BAMBI | 10.78938 | 51.21488 | 2.246951 | 2.45E-26 | 4.44E-24 |
| PDZD3 | 0.096765 | 0.953888 | 3.301255 | 5.45E-23 | 4.93E-21 |
| STX11 | 3.102352 | 1.21264 | -1.35521 | 2.17E-21 | 1.50E-19 |
| MT1X | 49.00954 | 18.38165 | -1.4148 | 1.30E-15 | 2.77E-14 |
| FBP2 | 0.225266 | 0.771329 | 1.77572 | 5.05E-10 | 3.77E-09 |
| EEF1A2 | 18.36426 | 44.01708 | 1.261162 | 2.68E-10 | 2.09E-09 |
| DCLK2 | 0.708215 | 1.542015 | 1.122557 | 1.44E-11 | 1.42E-10 |
| IGLV8-61 | 14.05357 | 5.410857 | -1.37701 | 0.001787 | 0.003739 |
| DHRS9 | 5.627805 | 1.47746 | -1.92945 | 3.22E-14 | 5.14E-13 |
| ERVV-1 | 0.315561 | 0.83173 | 1.398195 | 9.71E-06 | 3.30E-05 |
| DDX60 | 10.30254 | 3.647155 | -1.49816 | 7.33E-26 | 1.17E-23 |
| PTCHD1 | 0.26419 | 0.928109 | 1.812717 | 3.60E-13 | 4.72E-12 |
| HLA-DMB | 11.36392 | 4.006011 | -1.50422 | 2.44E-18 | 9.37E-17 |
| MSX2 | 8.905975 | 21.25868 | 1.255207 | 2.97E-17 | 9.36E-16 |
| AREG | 42.15295 | 14.45804 | -1.54376 | 4.11E-08 | 2.19E-07 |
| MT2P1 | 4.971776 | 1.349927 | -1.88088 | 8.51E-12 | 8.76E-11 |
| PRAP1 | 2.209587 | 6.359125 | 1.525052 | 2.28E-18 | 8.84E-17 |
| SYP | 0.289361 | 1.004883 | 1.796087 | 6.12E-08 | 3.16E-07 |
| IGLV3-12 | 0.841077 | 0.322057 | -1.38492 | 0.013917 | 0.023324 |
| SERPINB1 | 65.96613 | 22.70334 | -1.53882 | 1.37E-26 | 2.58E-24 |
| CHI3L2 | 4.338149 | 1.314349 | -1.72273 | 1.29E-05 | 4.27E-05 |
| RND2 | 0.469378 | 0.951718 | 1.019784 | 8.36E-06 | 2.89E-05 |
| SLC25A35 | 1.793159 | 3.620476 | 1.013676 | 1.16E-11 | 1.17E-10 |
| TBX1 | 4.963382 | 11.84863 | 1.255324 | 8.56E-08 | 4.30E-07 |
| CCL13 | 10.01439 | 3.58689 | -1.48127 | 1.79E-09 | 1.22E-08 |
| SLAMF1 | 1.10334 | 0.294492 | -1.90558 | 2.05E-17 | 6.60E-16 |
| ENHO | 0.705617 | 1.6437 | 1.21999 | 0.001171 | 0.002558 |
| FBXL16 | 0.501248 | 1.1769 | 1.231395 | 0.000202 | 0.000526 |
| IGFL1 | 31.37438 | 114.1476 | 1.863242 | 1.34E-05 | 4.40E-05 |
| OSMR | 13.08107 | 5.110636 | -1.35591 | 2.30E-21 | 1.58E-19 |
| ZBED2 | 5.097262 | 1.443266 | -1.82039 | 7.17E-16 | 1.61E-14 |
| LCP1 | 43.22484 | 21.31787 | -1.0198 | 6.18E-15 | 1.12E-13 |
| BCAS1 | 6.612709 | 13.57333 | 1.037461 | 2.95E-12 | 3.29E-11 |
| ZNF44 | 2.058746 | 4.731305 | 1.200472 | 1.44E-13 | 2.07E-12 |
| TNFRSF17 | 1.364009 | 0.471574 | -1.5323 | 0.000752 | 0.001715 |
| IGLV1-47 | 60.89576 | 18.68233 | -1.70467 | 8.24E-06 | 2.85E-05 |
| CORO1A | 18.66194 | 6.847089 | -1.44654 | 1.24E-13 | 1.81E-12 |
| SAA1 | 89.75886 | 10.78668 | -3.0568 | 1.54E-14 | 2.61E-13 |
| CCDC198 | 0.293839 | 1.356503 | 2.206793 | 2.11E-11 | 2.01E-10 |
| ETV7 | 11.38697 | 4.513234 | -1.33515 | 1.43E-24 | 1.84E-22 |
| PIK3AP1 | 3.399054 | 1.184068 | -1.52138 | 2.28E-15 | 4.50E-14 |
| MUC15 | 1.67501 | 3.380867 | 1.013223 | 5.19E-06 | 1.87E-05 |
| CD82 | 37.67531 | 17.70791 | -1.08923 | 3.65E-24 | 4.24E-22 |
| EMX2 | 1.062016 | 4.381671 | 2.044676 | 7.89E-19 | 3.33E-17 |
| KRT16 | 344.4462 | 102.791 | -1.74456 | 2.01E-11 | 1.92E-10 |
| CD6 | 2.109797 | 0.769908 | -1.45435 | 9.37E-16 | 2.05E-14 |
| IGLC2 | 365.1201 | 145.0978 | -1.33135 | 5.61E-05 | 0.000164 |
| SERPING1 | 97.52156 | 45.0839 | -1.11311 | 4.29E-17 | 1.30E-15 |
| HNF1B | 1.149759 | 4.408167 | 1.938848 | 6.64E-20 | 3.40E-18 |
| DSE | 3.515575 | 1.527472 | -1.20262 | 2.39E-14 | 3.89E-13 |
| VSNL1 | 4.740642 | 1.279624 | -1.88936 | 4.30E-13 | 5.55E-12 |
| CD24 | 149.982 | 349.3596 | 1.219924 | 7.32E-17 | 2.05E-15 |
| SLCO2B1 | 4.544176 | 1.898527 | -1.25914 | 1.75E-10 | 1.42E-09 |
| C1QB | 159.1679 | 43.54558 | -1.86995 | 3.71E-19 | 1.64E-17 |
| TMEM254-AS1 | 0.797184 | 1.67186 | 1.068469 | 8.36E-10 | 6.06E-09 |
| PRNP | 103.3709 | 44.06615 | -1.23009 | 1.94E-22 | 1.66E-20 |
| FPR3 | 8.81765 | 3.149733 | -1.48516 | 2.10E-14 | 3.48E-13 |
| CD1C | 2.341774 | 0.766504 | -1.61124 | 0.000124 | 0.000338 |
| RHCG | 38.28933 | 9.454698 | -2.01784 | 3.14E-07 | 1.42E-06 |
| BMP5 | 0.348894 | 0.842772 | 1.272352 | 2.05E-07 | 9.54E-07 |
| C3AR1 | 7.101048 | 2.470502 | -1.52323 | 5.06E-15 | 9.30E-14 |
| IGKV6D-21 | 2.161408 | 0.975817 | -1.14729 | 0.008355 | 0.01481 |
| KRT33A | 0.334147 | 4.058817 | 3.602507 | 1.15E-10 | 9.61E-10 |
| SNHG18 | 10.4145 | 27.19531 | 1.384764 | 9.34E-19 | 3.89E-17 |
| CD37 | 7.100229 | 2.410914 | -1.55829 | 5.93E-09 | 3.62E-08 |
| LMO3 | 0.409218 | 0.99868 | 1.287151 | 3.08E-05 | 9.44E-05 |
| TAP1 | 84.62007 | 20.56515 | -2.0408 | 1.36E-39 | 1.89E-36 |
| ADRB2 | 1.93095 | 0.946004 | -1.02939 | 4.04E-10 | 3.08E-09 |
| VEGFC | 11.20553 | 4.042742 | -1.4708 | 7.70E-09 | 4.61E-08 |
| MYCL-AS1 | 0.261939 | 1.145782 | 2.129031 | 2.77E-18 | 1.05E-16 |
| SNTB1 | 1.258942 | 2.575158 | 1.032449 | 0.012447 | 0.021076 |
| ELF5 | 1.96281 | 4.869957 | 1.310989 | 6.08E-14 | 9.26E-13 |
| FGD2 | 1.211154 | 0.534117 | -1.18115 | 1.58E-15 | 3.28E-14 |
| BCL2A1 | 6.917111 | 2.191799 | -1.65805 | 4.31E-21 | 2.79E-19 |
| SH2D1A | 1.350321 | 0.367406 | -1.87785 | 1.14E-15 | 2.46E-14 |
| HKDC1 | 1.140698 | 0.482859 | -1.24024 | 4.64E-05 | 0.000138 |
| SLC10A1 | 0.059228 | 1.441343 | 4.605001 | 2.98E-09 | 1.95E-08 |
| SLC38A4 | 1.171074 | 7.016452 | 2.582909 | 0.007567 | 0.01354 |
| PSORS1C3 | 2.450685 | 5.527953 | 1.17356 | 3.46E-07 | 1.55E-06 |
| CNGA1 | 1.877954 | 6.616006 | 1.816799 | 8.06E-19 | 3.38E-17 |
| FOXP3 | 2.620951 | 1.203671 | -1.12265 | 3.31E-17 | 1.03E-15 |
| HLA-H | 63.9163 | 25.49651 | -1.32588 | 2.79E-25 | 3.88E-23 |
| TOX2 | 3.705009 | 1.349105 | -1.45747 | 3.36E-10 | 2.60E-09 |
| CD79A | 16.16343 | 3.587926 | -2.17151 | 0.001002 | 0.002223 |
| RNY3P8 | 1.225199 | 2.674518 | 1.126263 | 9.11E-06 | 3.11E-05 |
| MT-TM | 0.989201 | 3.791519 | 1.93844 | 1.28E-10 | 1.06E-09 |
| AK4 | 4.970867 | 2.34345 | -1.08486 | 5.67E-17 | 1.66E-15 |
| MTATP8P1 | 0.861561 | 1.834558 | 1.090407 | 3.08E-09 | 2.00E-08 |
| SH3PXD2A-AS1 | 2.954566 | 0.973443 | -1.60178 | 5.48E-16 | 1.27E-14 |
| TMEM238L | 2.289959 | 6.43793 | 1.491275 | 1.36E-17 | 4.54E-16 |
| GABBR2 | 1.663618 | 5.826204 | 1.808232 | 5.79E-16 | 1.33E-14 |
| MIR3609 | 0.202838 | 2.20274 | 3.4409 | 0.000717 | 0.001644 |
| CYP4F2 | 0.118498 | 1.260899 | 3.411522 | 8.74E-09 | 5.19E-08 |
| ACP3 | 1.037648 | 3.623294 | 1.803985 | 0.021106 | 0.033771 |
| NKILA | 1.773312 | 0.869716 | -1.02783 | 1.77E-10 | 1.43E-09 |
| CST7 | 10.37298 | 3.541672 | -1.55033 | 5.88E-17 | 1.70E-15 |
| PPP1R35-AS1 | 0.511921 | 1.027417 | 1.005029 | 3.73E-14 | 5.87E-13 |
| PHGR1 | 1.159029 | 7.670426 | 2.72639 | 1.06E-10 | 8.95E-10 |
| TSPEAR-AS2 | 0.272732 | 1.024604 | 1.909511 | 1.64E-06 | 6.53E-06 |
| IRF1 | 21.34484 | 6.792167 | -1.65194 | 7.00E-45 | 3.25E-41 |
| IGLV3-25 | 80.48341 | 37.51958 | -1.10105 | 7.52E-05 | 0.000214 |
| IFNG | 1.252403 | 0.122622 | -3.35241 | 6.99E-29 | 1.98E-26 |
| TNFSF9 | 2.982496 | 1.430901 | -1.0596 | 2.55E-07 | 1.17E-06 |
| ST3GAL4 | 13.98159 | 29.55913 | 1.080075 | 2.15E-12 | 2.45E-11 |
| AIF1 | 24.47711 | 9.019301 | -1.44035 | 1.90E-17 | 6.16E-16 |
| CD1A | 2.020119 | 0.54968 | -1.87778 | 1.18E-11 | 1.18E-10 |
| IGHV1-69D | 38.35246 | 12.52354 | -1.61468 | 0.000287 | 0.000723 |
| PRRT2 | 0.397972 | 0.855447 | 1.104011 | 4.89E-05 | 0.000144 |
| PTN | 20.33153 | 64.12775 | 1.65723 | 7.27E-08 | 3.71E-07 |
| LRRC25 | 3.29951 | 1.301124 | -1.34249 | 3.28E-15 | 6.25E-14 |
| CD72 | 2.174826 | 0.908788 | -1.25888 | 2.63E-07 | 1.20E-06 |
| SERPINB5 | 55.46112 | 19.3519 | -1.519 | 7.85E-18 | 2.71E-16 |
| IGLV3-10 | 33.7104 | 14.39887 | -1.22724 | 0.001597 | 0.00338 |
| IGHV3OR16-9 | 0.888849 | 0.336901 | -1.39961 | 0.000129 | 0.00035 |
| NKD2 | 2.373696 | 6.989528 | 1.55806 | 7.58E-09 | 4.55E-08 |
| RPL39P40 | 0.727515 | 1.894433 | 1.380717 | 4.84E-06 | 1.76E-05 |
| CD44-AS1 | 1.302095 | 0.639287 | -1.0263 | 3.47E-14 | 5.50E-13 |
| C1orf162 | 4.950896 | 2.411645 | -1.03767 | 6.09E-15 | 1.11E-13 |
| REG4 | 0.17847 | 1.247762 | 2.80559 | 1.06E-06 | 4.37E-06 |
| BTN3A2 | 10.09178 | 4.825042 | -1.06457 | 2.02E-23 | 2.08E-21 |
| CTSC | 35.15821 | 14.17996 | -1.31001 | 6.44E-21 | 3.95E-19 |
| HLA-DQB2 | 10.86306 | 3.834441 | -1.50234 | 1.42E-15 | 2.99E-14 |
| SPHK1 | 12.46864 | 5.622087 | -1.14913 | 8.83E-13 | 1.08E-11 |
| IGKV3-15 | 49.84274 | 23.65521 | -1.07523 | 8.14E-05 | 0.00023 |
| NPM2 | 0.607656 | 1.604772 | 1.401042 | 1.45E-07 | 6.94E-07 |
| KCNC3 | 0.982498 | 2.167888 | 1.141764 | 8.67E-14 | 1.29E-12 |
| SLCO4C1 | 0.307108 | 1.070678 | 1.801708 | 0.000409 | 0.000998 |
| KRT6B | 149.2254 | 22.39288 | -2.73638 | 3.45E-18 | 1.26E-16 |
| EFNA2 | 0.255233 | 0.960237 | 1.911574 | 5.91E-12 | 6.27E-11 |
| PSCA | 475.3607 | 1030.933 | 1.116856 | 1.04E-11 | 1.05E-10 |
| IFIH1 | 10.59408 | 3.8052 | -1.47721 | 5.13E-28 | 1.30E-25 |
| RNF157 | 0.821811 | 1.662097 | 1.016127 | 6.62E-07 | 2.82E-06 |
| NRG1 | 1.295743 | 0.356253 | -1.86281 | 2.13E-09 | 1.43E-08 |
| TTR | 0.129813 | 12.36952 | 6.574208 | 3.69E-21 | 2.43E-19 |
| GCNT1 | 2.338807 | 1.121893 | -1.05984 | 7.18E-15 | 1.29E-13 |
| CCR4 | 0.76866 | 0.365282 | -1.07334 | 1.54E-07 | 7.32E-07 |
| CYP4F22 | 6.561353 | 19.61362 | 1.57979 | 1.29E-12 | 1.54E-11 |
| LINC01094 | 0.861484 | 0.369593 | -1.22089 | 3.32E-13 | 4.39E-12 |
| EREG | 8.524363 | 1.771637 | -2.26651 | 1.27E-07 | 6.20E-07 |
| SP140 | 1.589937 | 0.35924 | -2.14595 | 1.62E-24 | 2.06E-22 |
| TSPAN8 | 1.273613 | 5.131429 | 2.010434 | 7.75E-10 | 5.65E-09 |
| SELE | 1.662658 | 3.867629 | 1.217958 | 0.012784 | 0.021569 |
| WIPF1 | 5.994412 | 2.9781 | -1.00923 | 7.41E-13 | 9.21E-12 |
| NCF1B | 1.003431 | 0.221139 | -2.18192 | 1.50E-15 | 3.12E-14 |
| RCSD1 | 2.941541 | 1.23138 | -1.2563 | 5.74E-09 | 3.53E-08 |
| FOSL1 | 32.70241 | 14.15335 | -1.20825 | 9.86E-17 | 2.71E-15 |
| IGLV1-44 | 65.26766 | 26.58902 | -1.29554 | 5.75E-06 | 2.05E-05 |
| LINC02875 | 0.791147 | 1.918069 | 1.277637 | 3.24E-16 | 7.86E-15 |
| IGLC3 | 253.5877 | 104.2254 | -1.28278 | 1.31E-06 | 5.29E-06 |
| CSF3 | 4.724054 | 2.227305 | -1.08473 | 1.01E-05 | 3.42E-05 |
| SDR16C5 | 3.198177 | 1.417044 | -1.17437 | 1.94E-09 | 1.31E-08 |
| SCNN1G | 6.032109 | 23.22766 | 1.945109 | 6.44E-19 | 2.76E-17 |
| NLRP3 | 0.911359 | 0.357581 | -1.34975 | 4.01E-16 | 9.52E-15 |
| DOK2 | 6.336375 | 2.072427 | -1.61234 | 1.51E-20 | 8.64E-19 |
| CXCL9 | 47.73846 | 6.334019 | -2.91396 | 3.38E-24 | 4.01E-22 |
| MS4A4A | 6.508698 | 2.599028 | -1.3244 | 3.51E-11 | 3.20E-10 |
| HLA-F | 44.34441 | 9.905554 | -2.16244 | 1.08E-39 | 1.67E-36 |
| OSGIN1 | 5.305336 | 2.539171 | -1.06309 | 6.51E-05 | 0.000187 |
| SUSD3 | 4.760782 | 1.944078 | -1.29211 | 1.57E-15 | 3.26E-14 |
| CD300LF | 1.712402 | 0.628162 | -1.44681 | 4.58E-17 | 1.38E-15 |
| IGKJ5 | 3.715726 | 1.339234 | -1.47224 | 0.000214 | 0.000556 |
| USP30-AS1 | 3.28964 | 0.620631 | -2.40612 | 1.30E-37 | 1.21E-34 |
| FNDC5 | 0.517661 | 1.110224 | 1.10077 | 5.51E-07 | 2.38E-06 |
| C1QA | 195.0129 | 56.83169 | -1.7788 | 2.35E-18 | 9.07E-17 |
| GNA14 | 1.230676 | 2.71554 | 1.141788 | 5.22E-08 | 2.73E-07 |
| TAC3 | 13.85829 | 61.34684 | 2.14624 | 1.32E-21 | 9.80E-20 |
| ALDH1A2 | 0.818863 | 3.65115 | 2.156657 | 1.02E-08 | 6.00E-08 |
| RHOH | 1.294237 | 0.341424 | -1.92247 | 1.89E-17 | 6.13E-16 |
| FBLL1 | 0.409917 | 1.333958 | 1.702308 | 8.45E-12 | 8.71E-11 |
| KRT6C | 30.42089 | 7.348064 | -2.04963 | 8.43E-18 | 2.89E-16 |
| HMGA2 | 2.458895 | 0.464271 | -2.40497 | 1.70E-11 | 1.66E-10 |
| ADAM11 | 0.282846 | 0.708772 | 1.325304 | 2.67E-14 | 4.32E-13 |
| MIR4635 | 0.673672 | 1.500403 | 1.155231 | 1.47E-06 | 5.91E-06 |
| RAP1GAP | 3.349835 | 8.484481 | 1.340736 | 1.14E-18 | 4.71E-17 |
| SAMHD1 | 26.88963 | 12.92546 | -1.05683 | 5.75E-20 | 2.97E-18 |
| IRF4 | 0.992025 | 0.344974 | -1.52389 | 9.00E-11 | 7.69E-10 |
| CD163L1 | 1.530653 | 0.654683 | -1.22528 | 3.85E-09 | 2.45E-08 |
| FOXL1 | 2.755622 | 1.25203 | -1.13811 | 4.16E-05 | 0.000124 |
| IL37 | 0.176654 | 1.058751 | 2.583369 | 3.71E-05 | 0.000112 |
| DCBLD2 | 7.946478 | 3.39655 | -1.22625 | 9.06E-06 | 3.10E-05 |
| CT69 | 1.30487 | 0.1251 | -3.38275 | 5.65E-09 | 3.48E-08 |
| ARSI | 3.845104 | 1.767641 | -1.1212 | 2.33E-09 | 1.54E-08 |
| NDRG2 | 11.32368 | 24.60225 | 1.119447 | 2.60E-15 | 5.05E-14 |
| RIMKLA | 0.222345 | 0.732285 | 1.719607 | 3.70E-17 | 1.14E-15 |
| ZNF90 | 0.960113 | 2.26689 | 1.239439 | 3.27E-13 | 4.33E-12 |
| LINC01705 | 0.960709 | 0.399572 | -1.26564 | 3.19E-06 | 1.20E-05 |
| RGL3 | 3.207588 | 9.385776 | 1.548987 | 1.45E-20 | 8.33E-19 |
| HAVCR1 | 0.166736 | 3.911366 | 4.552034 | 5.95E-09 | 3.64E-08 |
| CD101 | 0.883902 | 0.423546 | -1.06137 | 3.65E-20 | 1.94E-18 |
| AIFM3 | 1.237601 | 3.625462 | 1.550618 | 7.94E-11 | 6.86E-10 |
| SALL2 | 1.146486 | 2.308034 | 1.009446 | 2.50E-13 | 3.39E-12 |
| UGT2B7 | 0.938479 | 4.517837 | 2.267235 | 1.29E-11 | 1.29E-10 |
| PRR15L | 5.716714 | 16.32552 | 1.513871 | 3.35E-12 | 3.71E-11 |
| DSG3 | 37.8671 | 8.710525 | -2.12011 | 1.09E-15 | 2.35E-14 |
| IGHV3-73 | 14.97162 | 5.612111 | -1.41561 | 0.000529 | 0.001251 |
| FRAS1 | 0.611571 | 1.406642 | 1.201664 | 1.03E-05 | 3.47E-05 |
| AOC2 | 1.017273 | 2.33665 | 1.199735 | 1.19E-09 | 8.36E-09 |
| LINC01857 | 0.975641 | 0.429283 | -1.18442 | 0.006175 | 0.011319 |
| FAM3B | 9.128798 | 21.59854 | 1.242437 | 7.00E-17 | 1.98E-15 |
| ICOS | 1.209811 | 0.31177 | -1.95623 | 1.41E-24 | 1.84E-22 |
| SRGN | 100.1611 | 37.29892 | -1.42512 | 4.68E-17 | 1.41E-15 |
| COL9A2 | 4.776896 | 12.45376 | 1.382435 | 0.000256 | 0.000651 |
| SPINK6 | 4.145427 | 0.25653 | -4.01432 | 0.00199 | 0.004122 |
| SPP1 | 267.8445 | 54.85014 | -2.28783 | 4.73E-08 | 2.49E-07 |
| SCIN | 0.870315 | 2.676867 | 1.620935 | 1.73E-08 | 9.82E-08 |
| FGL2 | 6.678454 | 2.8488 | -1.22916 | 3.37E-13 | 4.45E-12 |
| ZNF350-AS1 | 4.383995 | 22.96028 | 2.388823 | 2.40E-12 | 2.72E-11 |
| LINC01833 | 0.812571 | 2.043483 | 1.330464 | 1.11E-09 | 7.80E-09 |
| MMP3 | 18.76996 | 5.203496 | -1.85087 | 5.88E-05 | 0.000171 |
| KIFC2 | 7.692247 | 15.96524 | 1.053457 | 3.06E-13 | 4.10E-12 |
| SCNN1B | 11.79608 | 38.02087 | 1.688484 | 5.46E-16 | 1.27E-14 |
| GOLT1A | 7.795073 | 19.15999 | 1.297462 | 5.75E-17 | 1.68E-15 |
| TFF2 | 3.950426 | 8.88429 | 1.169248 | 3.63E-05 | 0.00011 |
| POU2F2 | 1.455604 | 0.678903 | -1.10034 | 0.000496 | 0.00118 |
| LINC02577 | 1.196451 | 0.189949 | -2.65508 | 2.23E-33 | 1.29E-30 |
| LINC01341 | 0.819125 | 2.043846 | 1.31913 | 3.39E-14 | 5.39E-13 |
| NLRC5 | 6.171886 | 1.879656 | -1.71524 | 2.41E-39 | 3.04E-36 |
| FBLN1 | 59.4049 | 162.7212 | 1.453748 | 2.08E-16 | 5.24E-15 |
| CPA4 | 5.972512 | 1.302693 | -2.19684 | 1.18E-09 | 8.27E-09 |
| ZNF761 | 3.597627 | 8.034284 | 1.159124 | 8.22E-15 | 1.47E-13 |
| IL15RA | 9.558472 | 2.419323 | -1.98218 | 4.30E-45 | 2.99E-41 |
| CARD17 | 2.111761 | 0.346263 | -2.6085 | 1.13E-28 | 3.03E-26 |
| ZNF682 | 1.173082 | 2.34677 | 1.000373 | 3.04E-16 | 7.42E-15 |
| OR2I1P | 14.51946 | 2.003123 | -2.85766 | 7.54E-19 | 3.20E-17 |
| TMSB15A | 2.341868 | 6.450889 | 1.461838 | 0.001533 | 0.003257 |
| PLIN5 | 1.309832 | 3.506824 | 1.420783 | 3.04E-16 | 7.42E-15 |
| UNC5CL | 0.547549 | 1.211967 | 1.146292 | 4.25E-06 | 1.56E-05 |
| IGLV3-21 | 135.0341 | 51.65729 | -1.38628 | 3.88E-05 | 0.000116 |
| IGHV3-48 | 22.05755 | 4.018256 | -2.45663 | 0.000167 | 0.000443 |
| THBD | 39.85794 | 19.18028 | -1.05524 | 1.51E-08 | 8.67E-08 |
| OASL | 11.59463 | 4.696991 | -1.30365 | 6.75E-20 | 3.44E-18 |
| SH3GL2 | 0.682399 | 2.399743 | 1.814193 | 3.35E-07 | 1.51E-06 |
| UPK1B | 86.16749 | 196.866 | 1.191998 | 9.59E-08 | 4.78E-07 |
| LINC00973 | 1.125071 | 0.055602 | -4.33873 | 8.90E-12 | 9.12E-11 |
| LINC00460 | 1.611447 | 0.399995 | -2.0103 | 1.43E-19 | 6.93E-18 |
| BST2 | 281.167 | 126.5567 | -1.15164 | 6.13E-22 | 4.84E-20 |
| DCDC2 | 0.471267 | 0.959513 | 1.025759 | 2.47E-14 | 4.01E-13 |
| EFHD1 | 1.983312 | 4.544064 | 1.196071 | 4.34E-07 | 1.91E-06 |
| IFI44 | 39.14975 | 15.29908 | -1.35556 | 4.63E-26 | 7.96E-24 |
| FADS3 | 6.685465 | 3.31939 | -1.01011 | 0.001612 | 0.003407 |
| MYLPF | 0.429081 | 1.689088 | 1.976923 | 4.83E-13 | 6.20E-12 |
| TMEM45B | 9.482758 | 20.36326 | 1.10259 | 1.32E-13 | 1.90E-12 |
| MESP1 | 1.237255 | 3.567258 | 1.527673 | 1.24E-05 | 4.09E-05 |
| IGHV5-51 | 90.58135 | 33.37628 | -1.44039 | 0.000815 | 0.001848 |
| CECR2 | 0.393816 | 1.112545 | 1.49827 | 7.91E-14 | 1.19E-12 |
| ATP7B | 0.744447 | 1.875145 | 1.332761 | 7.97E-08 | 4.03E-07 |
| RRAD | 29.43326 | 5.329963 | -2.46525 | 1.25E-06 | 5.06E-06 |
| BAALC | 3.315284 | 1.128036 | -1.55532 | 0.000456 | 0.001097 |
| LINC01871 | 6.232978 | 1.10179 | -2.50007 | 3.91E-29 | 1.16E-26 |
| MYCN | 1.388377 | 5.786462 | 2.059282 | 9.81E-20 | 4.86E-18 |
| IGKV1OR2-108 | 3.00099 | 1.465368 | -1.03418 | 0.000618 | 0.001436 |
| PLEKHB1 | 1.097704 | 2.495291 | 1.184719 | 6.84E-09 | 4.13E-08 |
| PNMT | 3.727139 | 14.62199 | 1.971999 | 6.48E-12 | 6.83E-11 |
| IGKV6-21 | 6.439866 | 1.713319 | -1.91024 | 0.025937 | 0.040513 |
| SERPINB13 | 10.73754 | 2.538371 | -2.08069 | 1.96E-16 | 4.99E-15 |
| TRDC | 2.178035 | 0.533552 | -2.02933 | 7.00E-17 | 1.98E-15 |
| MT-TT | 3.309215 | 7.500017 | 1.180405 | 1.64E-06 | 6.51E-06 |
| CLDN1 | 118.6484 | 53.2089 | -1.15695 | 7.02E-09 | 4.24E-08 |
| SOST | 5.728438 | 0.126756 | -5.49802 | 1.27E-07 | 6.20E-07 |
| PTPRC | 5.628145 | 1.605747 | -1.80941 | 1.18E-18 | 4.84E-17 |
| UGT1A1 | 0.425309 | 1.033365 | 1.280768 | 0.000128 | 0.000348 |
| IGHV4-59 | 40.4648 | 15.1615 | -1.41625 | 7.22E-05 | 0.000206 |
| COLEC11 | 0.557775 | 1.17702 | 1.077385 | 1.03E-13 | 1.51E-12 |
| FOLR2 | 13.99194 | 6.410112 | -1.12618 | 1.32E-05 | 4.36E-05 |
| OSR1 | 3.070613 | 6.511621 | 1.08449 | 8.95E-06 | 3.06E-05 |
| GPR68 | 7.911849 | 2.501372 | -1.6613 | 1.86E-13 | 2.61E-12 |
| NLRC3 | 0.943137 | 0.371406 | -1.34447 | 1.30E-16 | 3.45E-15 |
| SYTL5 | 1.310946 | 3.691575 | 1.493628 | 1.08E-12 | 1.30E-11 |
| IGLV7-43 | 29.74319 | 4.931432 | -2.59248 | 0.002682 | 0.005377 |
| SIRPG | 2.040452 | 0.774627 | -1.39732 | 1.12E-17 | 3.78E-16 |
| TRIM22 | 15.08262 | 4.579577 | -1.7196 | 2.09E-31 | 9.69E-29 |
| BIRC3 | 8.979582 | 2.863753 | -1.64874 | 5.90E-13 | 7.46E-12 |
| C1QC | 154.8427 | 44.46707 | -1.79999 | 2.09E-19 | 9.80E-18 |
| ARMCX4 | 0.556793 | 1.151438 | 1.048224 | 1.27E-13 | 1.85E-12 |
| MYBPC1 | 1.582306 | 4.020111 | 1.345207 | 7.72E-09 | 4.62E-08 |
| FREM2 | 0.187304 | 1.59867 | 3.093416 | 5.29E-28 | 1.32E-25 |
| SNORD123 | 2.408086 | 5.854001 | 1.281536 | 1.01E-12 | 1.23E-11 |
| IGHV3-49 | 23.93211 | 9.570924 | -1.32222 | 0.000399 | 0.000975 |
| GDF15 | 56.1536 | 137.1009 | 1.287787 | 1.75E-11 | 1.70E-10 |
| MYOSLID | 2.509743 | 0.485587 | -2.36974 | 1.01E-24 | 1.35E-22 |
| ACER2 | 8.554518 | 21.58786 | 1.335462 | 1.05E-11 | 1.06E-10 |
| TUBB6 | 34.72301 | 15.93875 | -1.12335 | 3.80E-10 | 2.91E-09 |
| CHST8 | 0.491547 | 1.159883 | 1.23858 | 2.93E-08 | 1.60E-07 |
| IRX3 | 11.74432 | 4.637334 | -1.3406 | 4.36E-11 | 3.91E-10 |
| CYP2J2 | 7.161848 | 15.59623 | 1.122794 | 6.31E-11 | 5.54E-10 |
| IFIT3 | 36.20976 | 10.79205 | -1.74641 | 2.67E-26 | 4.76E-24 |
| BCL2L15 | 0.371326 | 1.00089 | 1.430525 | 0.009617 | 0.01678 |
| PTPRR | 1.979895 | 5.139792 | 1.376286 | 1.10E-05 | 3.70E-05 |
| MROH3P | 0.440752 | 0.953343 | 1.113029 | 0.000361 | 0.000892 |
| S100B | 4.359739 | 1.526502 | -1.51401 | 5.88E-17 | 1.70E-15 |
| KLHL6 | 0.901179 | 0.438583 | -1.03896 | 1.35E-05 | 4.45E-05 |
| KLK2 | 0.098806 | 0.985811 | 3.318639 | 0.000572 | 0.001341 |
| IGHV1-69 | 14.52343 | 4.50387 | -1.68915 | 0.004054 | 0.007771 |
| VSIG2 | 53.78558 | 114.1468 | 1.085599 | 8.73E-16 | 1.92E-14 |
| CD300C | 1.525433 | 0.544874 | -1.48522 | 3.40E-16 | 8.22E-15 |
| KLK13 | 2.550906 | 0.741343 | -1.7828 | 0.005931 | 0.010923 |
| SNAI2 | 28.45243 | 11.05721 | -1.36356 | 5.31E-19 | 2.29E-17 |
| PRKCB | 1.280392 | 0.570635 | -1.16595 | 7.62E-10 | 5.57E-09 |
| SGSM1 | 0.203067 | 0.795906 | 1.970645 | 2.04E-19 | 9.61E-18 |
| ZNF681 | 0.91831 | 1.847205 | 1.00829 | 8.03E-16 | 1.79E-14 |
| HLA-DPA1 | 54.91029 | 18.8135 | -1.54531 | 4.68E-19 | 2.04E-17 |
| HLA-DRA | 682.1218 | 224.1223 | -1.60574 | 1.75E-21 | 1.24E-19 |
| IFITM1 | 169.2719 | 54.68942 | -1.63001 | 5.58E-24 | 6.21E-22 |
| GSTM2 | 4.143728 | 9.711994 | 1.228838 | 2.08E-08 | 1.16E-07 |
| AHNAK2 | 6.740942 | 2.313315 | -1.54299 | 6.38E-12 | 6.74E-11 |
| GJB2 | 181.4044 | 63.78741 | -1.50787 | 1.88E-13 | 2.64E-12 |
| FAM83A | 34.98698 | 10.51648 | -1.73417 | 9.31E-22 | 7.16E-20 |
| LINC01224 | 0.515631 | 1.332364 | 1.369577 | 3.75E-08 | 2.01E-07 |
| LINC01615 | 3.571635 | 1.119995 | -1.67309 | 8.60E-12 | 8.84E-11 |
| MS4A6A | 7.517386 | 2.802345 | -1.4236 | 1.41E-14 | 2.41E-13 |
| DEGS2 | 3.154439 | 6.97325 | 1.144448 | 4.01E-13 | 5.20E-12 |
| HEPHL1 | 2.547171 | 0.535454 | -2.25006 | 1.16E-11 | 1.17E-10 |
| CCL4 | 7.244489 | 1.547479 | -2.22696 | 1.23E-27 | 2.81E-25 |
| IHH | 0.308154 | 3.2319 | 3.390661 | 4.27E-14 | 6.67E-13 |
| MYO1G | 2.034349 | 0.652208 | -1.64116 | 1.64E-20 | 9.29E-19 |
| CST5 | 0.17201 | 1.296833 | 2.914428 | 0.01758 | 0.028715 |
| LINC02593 | 0.348219 | 1.026887 | 1.560213 | 8.61E-16 | 1.90E-14 |
| GPC2 | 0.81108 | 2.672848 | 1.720462 | 4.24E-10 | 3.21E-09 |
| DLX4 | 0.400342 | 1.062384 | 1.408002 | 0.000654 | 0.00151 |
| IL2RG | 18.82383 | 6.078446 | -1.63079 | 1.21E-20 | 7.05E-19 |
| LINC01612 | 1.064872 | 3.387069 | 1.669357 | 6.89E-12 | 7.22E-11 |
| MFSD6L | 0.67463 | 1.61329 | 1.257837 | 7.19E-11 | 6.25E-10 |
| HLA-DQB1-AS1 | 2.702424 | 0.827212 | -1.70792 | 1.17E-19 | 5.74E-18 |
| SP6 | 7.138488 | 15.53059 | 1.121423 | 1.57E-08 | 8.96E-08 |
| ACSM6 | 0.338398 | 1.948459 | 2.52554 | 3.38E-19 | 1.52E-17 |
| CXCL6 | 6.042299 | 2.577903 | -1.2289 | 1.91E-06 | 7.48E-06 |
| UPK1A | 49.13337 | 225.3889 | 2.197641 | 6.38E-23 | 5.69E-21 |
| HCG4 | 0.921634 | 0.397699 | -1.21252 | 1.37E-07 | 6.61E-07 |
| TRBV20-1 | 2.045908 | 0.701426 | -1.54438 | 1.02E-13 | 1.50E-12 |
| SULT1E1 | 1.701828 | 8.704512 | 2.354678 | 3.65E-08 | 1.96E-07 |
| CTSV | 6.879569 | 2.987862 | -1.2032 | 0.004241 | 0.008074 |
| XCL2 | 1.186613 | 0.483204 | -1.29614 | 1.47E-11 | 1.45E-10 |
| SLCO1B3 | 1.133176 | 0.126791 | -3.15985 | 9.31E-15 | 1.64E-13 |
| ARNTL2 | 10.47823 | 4.582923 | -1.19305 | 3.13E-18 | 1.17E-16 |
| LAT2 | 3.387061 | 1.151221 | -1.55687 | 3.49E-18 | 1.27E-16 |
| B3GAT1 | 0.335189 | 0.726161 | 1.115315 | 0.010769 | 0.018536 |
| CMPK2 | 6.519167 | 3.228852 | -1.01367 | 1.11E-14 | 1.92E-13 |
| SAMD13 | 0.700322 | 1.525556 | 1.123246 | 1.92E-09 | 1.30E-08 |
| LEAP2 | 0.754697 | 3.640609 | 2.27021 | 1.50E-15 | 3.12E-14 |
| ZDHHC8P1 | 0.259111 | 0.751176 | 1.535583 | 4.12E-10 | 3.13E-09 |
| IGHV3-74 | 29.27126 | 8.977882 | -1.70504 | 0.007413 | 0.013293 |
| IGHJ3P | 3.657161 | 1.282412 | -1.51186 | 0.003861 | 0.007452 |
| PALM3 | 5.273572 | 17.56851 | 1.736139 | 3.43E-19 | 1.53E-17 |
| CRH | 4.674856 | 58.01057 | 3.633322 | 5.55E-17 | 1.64E-15 |
| CRABP1 | 0.150298 | 1.877951 | 3.643263 | 3.27E-09 | 2.11E-08 |
| LINC01943 | 0.809626 | 0.378596 | -1.09659 | 6.12E-12 | 6.48E-11 |
| CHAD | 0.472991 | 1.175221 | 1.313046 | 1.31E-12 | 1.56E-11 |
| ADGRE1 | 1.184316 | 0.348113 | -1.76643 | 3.76E-17 | 1.15E-15 |
| TPBGL | 0.336413 | 0.971589 | 1.530111 | 0.01767 | 0.028836 |
| DTX1 | 1.524378 | 0.762016 | -1.00033 | 3.18E-05 | 9.72E-05 |
| RUNX3 | 4.556916 | 1.391783 | -1.71112 | 8.80E-26 | 1.35E-23 |
| UGT2A3 | 0.011175 | 2.981551 | 8.059601 | 6.08E-12 | 6.45E-11 |
| RBBP8NL | 3.393141 | 8.680535 | 1.355163 | 1.51E-23 | 1.57E-21 |
| ICAM3 | 0.752915 | 0.306119 | -1.29839 | 7.51E-12 | 7.80E-11 |
| SLC39A5 | 0.081258 | 0.920699 | 3.50214 | 1.75E-07 | 8.28E-07 |
| TMEM35A | 0.564185 | 1.446395 | 1.358221 | 1.70E-05 | 5.49E-05 |
| TENM2 | 2.999426 | 0.614326 | -2.28761 | 2.12E-07 | 9.85E-07 |
| SLC46A2 | 0.301286 | 0.661732 | 1.135112 | 0.000188 | 0.000492 |
| SH2D5 | 0.980028 | 0.199874 | -2.29373 | 4.55E-10 | 3.41E-09 |
| HS3ST6 | 1.875308 | 6.558934 | 1.806334 | 2.72E-16 | 6.73E-15 |
| TYMP | 89.62474 | 23.65207 | -1.92193 | 2.95E-31 | 1.28E-28 |
| ARID3A | 2.309271 | 5.5526 | 1.265726 | 0.000102 | 0.000284 |
| LCP2 | 3.847135 | 1.350664 | -1.51012 | 2.34E-21 | 1.60E-19 |
| IGHV3-33 | 35.33497 | 8.851447 | -1.99711 | 3.64E-06 | 1.36E-05 |
| RNU5A-1 | 0.220712 | 3.965992 | 4.167442 | 0.000727 | 0.001664 |
| GDF7 | 0.458735 | 1.247507 | 1.443314 | 5.47E-19 | 2.35E-17 |
| MCF2L-AS1 | 2.046481 | 4.534613 | 1.147834 | 1.56E-17 | 5.14E-16 |
| MCTP1 | 1.155058 | 0.543294 | -1.08816 | 1.82E-15 | 3.71E-14 |
| KCNH6 | 0.01041 | 1.819814 | 7.449617 | 0.000269 | 0.000684 |
| B3GALT5 | 0.519631 | 1.238201 | 1.252685 | 1.04E-11 | 1.06E-10 |
| HLA-A | 851.0483 | 308.1695 | -1.46552 | 2.17E-32 | 1.06E-29 |
| SLA | 2.963734 | 1.137889 | -1.38106 | 5.17E-18 | 1.84E-16 |
| KRT33B | 0.338337 | 1.294554 | 1.935921 | 1.54E-10 | 1.26E-09 |
| CD274 | 5.200366 | 1.435219 | -1.85734 | 1.50E-23 | 1.57E-21 |
| IGLV1-51 | 81.80472 | 31.8016 | -1.36308 | 0.000358 | 0.000884 |
| ZNF69 | 1.631669 | 3.447861 | 1.079353 | 1.29E-21 | 9.59E-20 |
| ABCA12 | 1.58105 | 0.768624 | -1.04053 | 1.18E-05 | 3.94E-05 |
| BOK-AS1 | 0.307796 | 0.819638 | 1.413014 | 0.030805 | 0.047289 |
| SLC28A2 | 0.038084 | 1.158427 | 4.926828 | 0.000156 | 0.000417 |
| LIN7A | 0.642833 | 1.631015 | 1.343255 | 2.56E-13 | 3.47E-12 |
| DDX58 | 11.10606 | 4.245633 | -1.3873 | 9.42E-23 | 8.30E-21 |
| SLC29A4 | 0.860308 | 1.990674 | 1.210333 | 2.68E-13 | 3.61E-12 |
| KRT5 | 1062.288 | 166.6141 | -2.67259 | 1.57E-22 | 1.36E-20 |
| TUBA3E | 0.306302 | 0.897606 | 1.551126 | 0.015443 | 0.025617 |
| APOA2 | 0.693928 | 36.603 | 5.721033 | 0.002606 | 0.005241 |
| CD84 | 1.297457 | 0.487534 | -1.41211 | 8.23E-14 | 1.23E-12 |
| HK3 | 3.138969 | 1.097885 | -1.51556 | 1.95E-16 | 4.97E-15 |
| ITGB2-AS1 | 1.14852 | 0.412779 | -1.47633 | 7.20E-12 | 7.52E-11 |
| FCGR2A | 9.811257 | 3.606189 | -1.44396 | 5.14E-19 | 2.23E-17 |
| DIRAS2 | 0.3007 | 0.749703 | 1.317995 | 1.34E-15 | 2.83E-14 |
| CD300E | 1.386367 | 0.531877 | -1.38214 | 8.14E-10 | 5.91E-09 |
| GUCA2A | 0.643005 | 13.883 | 4.432345 | 5.37E-21 | 3.39E-19 |
| HAVCR2 | 5.042653 | 1.703675 | -1.56553 | 2.21E-20 | 1.22E-18 |
| CCL3L1 | 1.583101 | 0.658148 | -1.26627 | 1.21E-10 | 1.01E-09 |
| IL1R2 | 3.174084 | 1.218456 | -1.38129 | 1.50E-11 | 1.48E-10 |
| HIC2 | 1.726724 | 4.801524 | 1.475455 | 1.81E-11 | 1.75E-10 |
| TRBV9 | 0.953074 | 0.225895 | -2.07694 | 1.39E-15 | 2.93E-14 |
| TRHDE | 0.312518 | 0.672274 | 1.105111 | 1.15E-09 | 8.07E-09 |
| UPK2 | 95.63644 | 527.1541 | 2.462592 | 6.51E-24 | 7.18E-22 |
| AIRE | 0.025267 | 0.870946 | 5.107263 | 0.002029 | 0.004193 |
| SLC19A2 | 5.596268 | 11.90136 | 1.08859 | 6.99E-21 | 4.25E-19 |
| EPGN | 2.033071 | 0.811993 | -1.32412 | 4.47E-09 | 2.80E-08 |
| SNORA12 | 0.296226 | 2.209331 | 2.898837 | 0.00462 | 0.008725 |
| MALL | 3.529588 | 1.433842 | -1.29961 | 5.94E-13 | 7.50E-12 |
| GRHL3 | 14.89789 | 40.18951 | 1.431711 | 3.11E-18 | 1.16E-16 |
| ZNF726 | 0.657126 | 1.422303 | 1.113986 | 5.63E-17 | 1.65E-15 |
| SPRR2E | 35.63416 | 6.197428 | -2.52352 | 0.000234 | 0.000602 |
| GZMA | 26.2796 | 2.533078 | -3.37498 | 4.50E-31 | 1.84E-28 |
| TEKT5 | 0.409353 | 1.51842 | 1.891153 | 1.81E-18 | 7.21E-17 |
| NUDT19-DT | 5.220874 | 11.32024 | 1.116541 | 1.13E-11 | 1.14E-10 |
| TBC1D10C | 2.742019 | 0.840444 | -1.70601 | 3.69E-15 | 7.02E-14 |
| LRRC4B | 0.325387 | 1.044918 | 1.68316 | 1.97E-08 | 1.10E-07 |
| GGT6 | 14.26503 | 30.18743 | 1.081465 | 3.68E-14 | 5.81E-13 |
| PTGDR2 | 0.156328 | 0.789791 | 2.336895 | 2.59E-08 | 1.42E-07 |
| PLEK | 8.384268 | 2.80582 | -1.57926 | 5.56E-16 | 1.28E-14 |
| LST1 | 5.132176 | 2.178638 | -1.23614 | 5.11E-17 | 1.51E-15 |
| LINC02446 | 2.835658 | 0.277964 | -3.35072 | 1.54E-21 | 1.13E-19 |
| MIR31HG | 3.115986 | 1.492936 | -1.06154 | 1.64E-06 | 6.51E-06 |
| APOD | 12.09432 | 36.64873 | 1.599434 | 6.20E-06 | 2.20E-05 |
| TMEM45A | 19.70951 | 6.194291 | -1.66988 | 5.74E-21 | 3.60E-19 |
| CD8A | 5.690338 | 1.125087 | -2.33848 | 1.75E-21 | 1.24E-19 |
| SIGLEC10 | 3.760529 | 0.856307 | -2.13474 | 2.51E-18 | 9.63E-17 |
| DOCK3 | 0.65675 | 1.699366 | 1.37158 | 4.15E-16 | 9.80E-15 |
| IGKV4-1 | 175.6225 | 59.41716 | -1.56353 | 9.55E-05 | 0.000266 |
| KRT81 | 46.91359 | 3.626237 | -3.69346 | 1.57E-11 | 1.54E-10 |
| FMO5 | 0.589451 | 1.451551 | 1.300152 | 8.20E-16 | 1.82E-14 |
| IGFL1P1 | 2.492747 | 5.226965 | 1.068237 | 0.005227 | 0.009768 |
| ZNF365 | 1.009284 | 0.46482 | -1.11859 | 1.79E-07 | 8.44E-07 |
| EVI2A | 3.608896 | 1.526033 | -1.24177 | 1.16E-12 | 1.40E-11 |
| FMO9P | 2.719259 | 17.99513 | 2.726321 | 1.34E-22 | 1.17E-20 |
| TMC8 | 4.172999 | 2.071644 | -1.01031 | 2.21E-11 | 2.08E-10 |
| CIITA | 3.067495 | 1.09931 | -1.48046 | 6.13E-21 | 3.78E-19 |
| ABHD11-AS1 | 3.729569 | 8.121673 | 1.122768 | 0.000433 | 0.001049 |
| IKZF1 | 1.507883 | 0.616845 | -1.28954 | 2.42E-14 | 3.93E-13 |
| IFIT1 | 11.15788 | 5.352932 | -1.05966 | 3.52E-12 | 3.87E-11 |
| SERPINB3 | 53.62422 | 10.18226 | -2.39683 | 1.80E-21 | 1.27E-19 |
| KLHDC7B | 70.1087 | 18.24283 | -1.94226 | 4.71E-20 | 2.46E-18 |
| CCDC190 | 1.512758 | 0.521209 | -1.53725 | 5.61E-07 | 2.42E-06 |
| TMPRSS11D | 2.847228 | 0.329244 | -3.11233 | 9.81E-10 | 7.01E-09 |
| FMNL1-DT | 1.146471 | 0.555312 | -1.04583 | 4.01E-06 | 1.48E-05 |
| FADS6 | 0.336787 | 1.501212 | 2.156219 | 2.22E-13 | 3.07E-12 |
| F5 | 1.425492 | 3.664102 | 1.362 | 0.003973 | 0.007638 |
| ADCY7 | 1.511477 | 0.630224 | -1.26202 | 9.73E-20 | 4.83E-18 |
| IL2RA | 3.428263 | 0.957149 | -1.84066 | 6.03E-21 | 3.73E-19 |
| ATP1A4 | 0.906422 | 2.449306 | 1.434119 | 7.63E-13 | 9.47E-12 |
| SOWAHA | 0.47605 | 1.163729 | 1.289569 | 3.07E-12 | 3.42E-11 |
| RUBCNL | 0.740592 | 0.356565 | -1.05451 | 3.00E-08 | 1.63E-07 |
| LINC01607 | 0.24326 | 0.775884 | 1.673343 | 1.47E-14 | 2.50E-13 |
| CACNG4 | 2.667486 | 5.715857 | 1.099489 | 8.71E-07 | 3.63E-06 |
| MAST1 | 0.321134 | 0.713371 | 1.151477 | 6.11E-09 | 3.73E-08 |
| IGKV1-5 | 119.9157 | 58.94918 | -1.02448 | 1.60E-05 | 5.20E-05 |
| ALOX5AP | 11.75758 | 4.417843 | -1.41218 | 5.05E-22 | 4.02E-20 |
| AADACP1 | 1.111771 | 0.323909 | -1.7792 | 1.93E-08 | 1.08E-07 |
| PIK3CD | 4.318031 | 1.774572 | -1.2829 | 2.85E-23 | 2.79E-21 |
| HLA-J | 4.596303 | 1.509964 | -1.60596 | 4.10E-16 | 9.71E-15 |
| ERAP2 | 11.1282 | 4.510528 | -1.30285 | 1.32E-12 | 1.57E-11 |
| LILRB4 | 3.858247 | 1.110398 | -1.79687 | 4.15E-16 | 9.80E-15 |
| CMKLR1 | 3.084645 | 1.075012 | -1.52075 | 1.19E-12 | 1.42E-11 |
| PDX1 | 0.291641 | 0.686562 | 1.235197 | 0.001005 | 0.002228 |
| TMEM171 | 2.294434 | 0.867324 | -1.4035 | 1.15E-15 | 2.46E-14 |
| GPR34 | 2.430079 | 1.059198 | -1.19803 | 2.02E-10 | 1.62E-09 |
| HLA-B | 1206.744 | 388.2252 | -1.63615 | 1.00E-32 | 5.15E-30 |
| TRBJ2-7 | 2.475918 | 1.007046 | -1.29783 | 6.14E-09 | 3.74E-08 |
| LYPD5 | 3.137907 | 1.4814 | -1.08284 | 2.16E-11 | 2.04E-10 |
| GATM | 3.04659 | 8.420042 | 1.466632 | 1.11E-09 | 7.83E-09 |
| FCGR3A | 30.59161 | 10.29931 | -1.57059 | 1.83E-17 | 5.99E-16 |
| ADRB1 | 0.621415 | 1.725595 | 1.473465 | 9.17E-07 | 3.80E-06 |
| ERVE-1 | 1.023522 | 3.248044 | 1.666029 | 1.25E-18 | 5.10E-17 |
| DIO3OS | 0.308792 | 0.675701 | 1.129748 | 0.003668 | 0.007112 |
| GZMB | 15.06387 | 6.387247 | -1.23783 | 1.08E-27 | 2.54E-25 |
| SNORA20 | 0.167645 | 0.921159 | 2.458045 | 0.004031 | 0.007735 |
| KCNH2 | 0.494063 | 1.928135 | 1.964437 | 0.000173 | 0.000457 |
| ITGB7 | 1.111855 | 0.54997 | -1.01554 | 1.52E-15 | 3.16E-14 |
| IFI16 | 47.47516 | 14.44074 | -1.71703 | 4.18E-41 | 9.70E-38 |
| GSDME | 3.288227 | 1.469594 | -1.16189 | 2.22E-13 | 3.07E-12 |
| MTND2P28 | 149.1681 | 332.5298 | 1.156545 | 2.13E-10 | 1.70E-09 |
| HIF3A | 0.315727 | 0.658168 | 1.059779 | 8.71E-07 | 3.63E-06 |
| TF | 0.251523 | 3.548253 | 3.818349 | 0.018967 | 0.030734 |
| VSIG4 | 13.94406 | 4.672866 | -1.57727 | 2.76E-15 | 5.36E-14 |
| MIA | 1.281788 | 0.292682 | -2.13075 | 0.005748 | 0.010627 |
| S1PR4 | 2.713317 | 1.22053 | -1.15255 | 1.66E-11 | 1.63E-10 |
| EMX2OS | 0.352907 | 1.214042 | 1.78246 | 7.30E-15 | 1.31E-13 |
| G0S2 | 42.56263 | 11.85988 | -1.8435 | 5.68E-13 | 7.21E-12 |
| IGKV2OR22-4 | 1.329273 | 0.326162 | -2.02698 | 0.032472 | 0.049487 |
| PTPN22 | 1.230795 | 0.418095 | -1.55769 | 5.01E-22 | 4.00E-20 |
| ZAP70 | 1.989935 | 0.773629 | -1.36301 | 1.71E-14 | 2.87E-13 |
| DPYSL5 | 0.112996 | 0.801952 | 2.827246 | 0.001695 | 0.003564 |
| SLC9A4 | 0.431597 | 3.575832 | 3.050521 | 3.68E-27 | 7.42E-25 |
| LILRB2 | 3.076865 | 0.91337 | -1.75219 | 2.19E-18 | 8.58E-17 |
| STAP1 | 1.236061 | 0.251792 | -2.29544 | 4.60E-06 | 1.68E-05 |
| SLC2A12 | 0.913148 | 0.436188 | -1.0659 | 8.08E-07 | 3.39E-06 |
| MTND6P4 | 0.565949 | 1.145122 | 1.016758 | 1.30E-06 | 5.24E-06 |
| PLS1 | 4.33354 | 8.985791 | 1.052099 | 4.35E-13 | 5.62E-12 |
| WNT6 | 1.12426 | 2.604963 | 1.212287 | 0.009402 | 0.016441 |
| BTK | 1.759311 | 0.639578 | -1.45982 | 7.65E-12 | 7.93E-11 |
| SNX31 | 9.949984 | 35.6011 | 1.839156 | 1.74E-18 | 6.94E-17 |
| LINC00930 | 1.570804 | 5.358834 | 1.770416 | 7.65E-13 | 9.48E-12 |
| ITGA2 | 15.16772 | 6.445444 | -1.23465 | 1.49E-17 | 4.95E-16 |
| HLA-DOB | 3.085157 | 1.24438 | -1.30992 | 3.37E-12 | 3.73E-11 |
| VTCN1 | 5.356985 | 11.24255 | 1.069476 | 3.20E-06 | 1.21E-05 |
| HUNK | 0.315349 | 0.920295 | 1.545146 | 5.09E-06 | 1.84E-05 |
| SAMSN1 | 3.836242 | 1.344091 | -1.51306 | 1.08E-19 | 5.32E-18 |
| SNHG9 | 6.925715 | 14.25807 | 1.041744 | 1.89E-17 | 6.13E-16 |
| CTXN1 | 5.55474 | 13.70576 | 1.302991 | 2.25E-13 | 3.10E-12 |
| LTK | 0.3318 | 0.907259 | 1.451202 | 0.000254 | 0.000647 |
| LAMC2 | 91.66333 | 21.53837 | -2.08944 | 4.57E-15 | 8.50E-14 |
| EPHA7 | 0.371699 | 0.849245 | 1.192046 | 2.90E-07 | 1.31E-06 |
| ITGAX | 3.472835 | 1.515249 | -1.19656 | 4.08E-13 | 5.29E-12 |
| LGALS7B | 19.0133 | 9.482085 | -1.00373 | 0.002864 | 0.005702 |
| GCNT2 | 1.294387 | 0.588383 | -1.13744 | 7.69E-16 | 1.72E-14 |
| TMEM150B | 1.396355 | 0.509974 | -1.45317 | 1.08E-15 | 2.33E-14 |
| OR7E39P | 0.317399 | 0.70407 | 1.14942 | 1.06E-06 | 4.36E-06 |
| KRT75 | 3.21527 | 1.096366 | -1.55221 | 1.79E-10 | 1.44E-09 |
| TRAV30 | 0.559654 | 3.018682 | 2.431311 | 0.000107 | 0.000294 |
| CCR7 | 4.117507 | 16.42257 | 1.995836 | 2.19E-05 | 6.93E-05 |
| RPL29P19 | 5.825708 | 2.71172 | -1.10322 | 9.60E-07 | 3.97E-06 |
| KRT86 | 6.510649 | 2.075299 | -1.64948 | 1.43E-06 | 5.75E-06 |
| LINC01213 | 1.409609 | 3.893829 | 1.465895 | 1.59E-20 | 9.01E-19 |
| ZNF439 | 1.459302 | 2.929092 | 1.005175 | 1.95E-15 | 3.94E-14 |
| KIF1A | 0.179498 | 1.171341 | 2.706122 | 1.16E-07 | 5.66E-07 |
| CLEC5A | 1.160093 | 0.49318 | -1.23405 | 1.76E-15 | 3.61E-14 |
| FAM20A | 2.9065 | 1.261464 | -1.20418 | 2.13E-11 | 2.02E-10 |
| KHDRBS3 | 0.508072 | 1.272636 | 1.324714 | 3.54E-06 | 1.32E-05 |
| PRSS1 | 3.379475 | 9.306935 | 1.461507 | 0.007124 | 0.012855 |
| STAB1 | 9.480829 | 4.674931 | -1.02007 | 1.91E-09 | 1.29E-08 |
| RGS11 | 0.296425 | 0.948987 | 1.67872 | 4.36E-10 | 3.29E-09 |
| KLHDC7A | 3.744552 | 9.699396 | 1.373102 | 3.14E-17 | 9.86E-16 |
| SRXN1 | 1.373757 | 0.672792 | -1.02989 | 5.16E-07 | 2.24E-06 |
| GSTA2 | 0.37541 | 1.963403 | 2.386818 | 1.44E-05 | 4.71E-05 |
| FDCSP | 124.8835 | 4.533048 | -4.78396 | 0.016116 | 0.026601 |
| LRFN2 | 0.201269 | 0.735103 | 1.868819 | 3.60E-19 | 1.60E-17 |
| HTR7 | 2.087508 | 0.835812 | -1.32053 | 4.58E-14 | 7.11E-13 |
| CCL15 | 0.540645 | 2.874986 | 2.410802 | 2.05E-16 | 5.19E-15 |
| LGALS4 | 3.751977 | 14.56894 | 1.957173 | 3.53E-08 | 1.90E-07 |
| TMPRSS2 | 7.173187 | 24.56194 | 1.775739 | 2.80E-23 | 2.76E-21 |
| POU2F1-DT | 0.529705 | 1.068027 | 1.011688 | 1.36E-11 | 1.35E-10 |
| LINC00942 | 3.156509 | 0.709631 | -2.15319 | 6.23E-05 | 0.00018 |
| KLK11 | 3.886592 | 1.099148 | -1.82212 | 3.13E-05 | 9.57E-05 |
| TLR6 | 0.859645 | 0.277779 | -1.6298 | 2.19E-22 | 1.84E-20 |
| KYNU | 3.385678 | 1.341254 | -1.33586 | 5.76E-16 | 1.32E-14 |
| LTB | 10.1636 | 4.675061 | -1.12035 | 7.09E-09 | 4.27E-08 |
| IDO1 | 32.50594 | 1.786611 | -4.18541 | 1.64E-35 | 1.14E-32 |
| MYEF2 | 0.286049 | 0.676567 | 1.241969 | 1.77E-06 | 6.97E-06 |
| MELTF | 7.758389 | 2.088994 | -1.89295 | 6.42E-12 | 6.78E-11 |
| CCL5 | 69.26309 | 32.99737 | -1.06974 | 3.36E-18 | 1.24E-16 |
| MS4A8 | 0.146515 | 2.600453 | 4.149644 | 5.93E-07 | 2.54E-06 |
| GASK1A | 0.56557 | 1.356811 | 1.262443 | 2.56E-12 | 2.88E-11 |
| SAMD9L | 8.05409 | 1.989172 | -2.01755 | 4.04E-37 | 3.51E-34 |
| YBX2 | 0.320472 | 0.677238 | 1.079463 | 0.000383 | 0.000941 |
| SMTNL2 | 0.470495 | 1.396925 | 1.570004 | 5.95E-15 | 1.08E-13 |
| SULT1A2 | 0.759563 | 1.9508 | 1.360824 | 1.61E-08 | 9.17E-08 |
| DIPK1B | 1.021248 | 2.561394 | 1.326597 | 5.57E-13 | 7.08E-12 |
| SPN | 1.435917 | 0.473641 | -1.60011 | 6.03E-21 | 3.73E-19 |
| LINC00165 | 1.048076 | 0.279008 | -1.90937 | 3.27E-07 | 1.47E-06 |
| TFPI2 | 17.81637 | 5.804664 | -1.61792 | 9.52E-10 | 6.83E-09 |
| HLA-C | 774.8685 | 371.0522 | -1.06233 | 8.93E-21 | 5.36E-19 |
| CFB | 4.357668 | 1.808095 | -1.26909 | 1.95E-18 | 7.72E-17 |
| CD68 | 0.972329 | 0.453996 | -1.09876 | 1.53E-19 | 7.33E-18 |
| TGFBR3 | 3.104983 | 6.389844 | 1.041195 | 3.95E-16 | 9.42E-15 |
| TIGIT | 1.663352 | 0.494478 | -1.75011 | 1.95E-20 | 1.09E-18 |
| SLC10A6 | 1.446298 | 0.518833 | -1.47902 | 0.00013 | 0.000351 |
| CLEC10A | 2.109328 | 0.956183 | -1.14142 | 2.36E-08 | 1.30E-07 |
| IFITM3P2 | 2.570431 | 1.070575 | -1.26362 | 0.000305 | 0.000765 |
| CLEC2B | 15.61925 | 4.927601 | -1.66437 | 3.82E-23 | 3.59E-21 |
| IGKV1-9 | 64.40385 | 15.98866 | -2.0101 | 0.000952 | 0.002121 |
| ARC | 0.305829 | 0.874659 | 1.515996 | 0.024805 | 0.039029 |
| SCGB2A1 | 0.134416 | 1.260135 | 3.228803 | 0.005047 | 0.009463 |
| IGKC | 800.3681 | 321.4562 | -1.31604 | 0.000119 | 0.000326 |
| WARS1 | 97.46183 | 15.8937 | -2.61638 | 2.74E-35 | 1.82E-32 |
| PIK3R5 | 1.143369 | 0.517196 | -1.14451 | 1.61E-13 | 2.30E-12 |
| POU2AF1 | 2.840656 | 0.836489 | -1.76381 | 0.000147 | 0.000394 |
| KLHDC7B-DT | 7.344377 | 1.387917 | -2.40372 | 1.70E-27 | 3.75E-25 |
| SLC6A14 | 3.664724 | 1.119623 | -1.71069 | 7.23E-05 | 0.000206 |
| MYO1B | 14.00002 | 6.209205 | -1.17295 | 1.12E-16 | 3.03E-15 |
| MT-ND4L | 1595.295 | 3344.471 | 1.067955 | 3.37E-12 | 3.73E-11 |
| MANCR | 1.247044 | 0.308231 | -2.01643 | 3.57E-17 | 1.11E-15 |
| GNGT2 | 1.226295 | 0.476475 | -1.36384 | 5.13E-18 | 1.83E-16 |
| CPNE8-AS1 | 0.981826 | 0.46045 | -1.09242 | 8.40E-11 | 7.24E-10 |
| PI4KAP2 | 0.826578 | 2.096084 | 1.342474 | 1.93E-13 | 2.71E-12 |
| CHN2 | 0.55522 | 1.191801 | 1.102011 | 5.06E-15 | 9.30E-14 |
| TMEM64 | 4.93735 | 10.31948 | 1.063562 | 5.35E-06 | 1.92E-05 |
| IGLV4-69 | 37.29735 | 11.39461 | -1.71072 | 0.002187 | 0.00448 |
| LINC00519 | 2.00533 | 0.989391 | -1.01923 | 5.92E-10 | 4.37E-09 |
| ADCY6-DT | 0.345489 | 0.751071 | 1.120312 | 4.63E-17 | 1.39E-15 |
| IGKV1-27 | 18.76048 | 8.646943 | -1.11743 | 0.00012 | 0.000327 |
| TEPP | 0.526385 | 2.322984 | 2.141788 | 1.42E-08 | 8.16E-08 |
| SERPINB2 | 11.70799 | 5.133794 | -1.1894 | 3.51E-09 | 2.25E-08 |
| IGF2BP2 | 10.19405 | 3.578256 | -1.5104 | 9.24E-17 | 2.57E-15 |
| SLC38A5 | 11.99049 | 4.873047 | -1.299 | 2.50E-13 | 3.39E-12 |
| IGLV2-11 | 43.49039 | 18.98859 | -1.19556 | 0.000121 | 0.000331 |
| STK17A | 11.58579 | 5.091802 | -1.18611 | 7.60E-26 | 1.19E-23 |
| PDE9A | 2.274262 | 4.8572 | 1.094727 | 8.34E-15 | 1.48E-13 |
| IGLV9-49 | 7.446955 | 2.288116 | -1.70249 | 3.45E-05 | 0.000105 |
| PACSIN1 | 0.485728 | 1.259544 | 1.374682 | 1.16E-15 | 2.49E-14 |
| TEX45 | 0.325921 | 1.455668 | 2.159085 | 7.67E-26 | 1.19E-23 |
| CYTH4 | 3.407077 | 1.210853 | -1.49251 | 7.76E-19 | 3.28E-17 |
| MYO1F | 3.03492 | 1.285918 | -1.23886 | 4.73E-15 | 8.74E-14 |
| FERMT3 | 12.21321 | 4.717328 | -1.3724 | 9.75E-15 | 1.70E-13 |
| SIGLEC9 | 1.206022 | 0.551423 | -1.12902 | 2.19E-14 | 3.59E-13 |
| OR13A1 | 0.530137 | 1.066517 | 1.00847 | 3.01E-10 | 2.33E-09 |
| CSF1 | 13.01144 | 4.985353 | -1.38401 | 1.26E-24 | 1.65E-22 |
| FUT6 | 1.034318 | 2.210661 | 1.095797 | 6.94E-06 | 2.44E-05 |
| RPL37P1 | 0.818601 | 1.685742 | 1.042152 | 2.65E-10 | 2.07E-09 |
| PKP1 | 54.16753 | 18.45686 | -1.55327 | 2.63E-10 | 2.06E-09 |
| ERICH5 | 2.281675 | 6.739477 | 1.562543 | 3.33E-22 | 2.72E-20 |
| IGHV4-61 | 4.528269 | 1.633268 | -1.4712 | 0.000461 | 0.001108 |
| KALRN | 0.371129 | 0.898005 | 1.274801 | 1.64E-05 | 5.31E-05 |
| RNU4-78P | 0.48091 | 1.067433 | 1.150305 | 3.19E-10 | 2.47E-09 |
| WFDC12 | 1.269267 | 0.4109 | -1.62714 | 3.66E-05 | 0.00011 |
| SLC7A7 | 3.698785 | 1.847018 | -1.00185 | 3.23E-12 | 3.58E-11 |
| PTPN7 | 2.689338 | 0.815682 | -1.72117 | 2.03E-22 | 1.71E-20 |
| CD163 | 12.75201 | 4.42341 | -1.52749 | 3.83E-13 | 5.01E-12 |
| FAM189A1 | 0.311722 | 1.287489 | 2.046229 | 1.02E-15 | 2.23E-14 |
| TRHDE-AS1 | 0.585483 | 1.980759 | 1.758354 | 6.79E-13 | 8.50E-12 |
| CASP1 | 12.89484 | 3.450478 | -1.90193 | 1.25E-40 | 2.48E-37 |
| SCAT1 | 1.197283 | 0.324987 | -1.88131 | 1.49E-18 | 6.02E-17 |
| CLCA2 | 53.19622 | 14.55233 | -1.87007 | 1.13E-16 | 3.06E-15 |
| COL17A1 | 44.92423 | 10.47855 | -2.10005 | 9.29E-13 | 1.14E-11 |
| ALDH3A1 | 45.49036 | 10.25444 | -2.14931 | 0.00423 | 0.008055 |
| NKAIN1 | 0.303528 | 0.851966 | 1.488968 | 0.002394 | 0.00485 |
| TMEM163 | 1.259623 | 2.62731 | 1.060594 | 8.70E-11 | 7.46E-10 |
| COL26A1 | 0.245483 | 0.910483 | 1.891008 | 0.000553 | 0.001302 |
| SMAD9 | 0.628508 | 1.661747 | 1.402696 | 6.13E-10 | 4.52E-09 |
| CCL18 | 30.37516 | 10.63066 | -1.51466 | 3.98E-10 | 3.04E-09 |
| DSCR8 | 0.872527 | 2.689951 | 1.624308 | 0.000173 | 0.000457 |
| CCL21 | 20.92045 | 9.667664 | -1.11368 | 0.000114 | 0.000314 |
| IGKV3D-20 | 12.94235 | 3.748918 | -1.78755 | 0.019022 | 0.030816 |
| TLE2 | 7.902756 | 20.21974 | 1.355337 | 5.17E-23 | 4.70E-21 |
| UGT2B15 | 0.457159 | 4.584836 | 3.326103 | 9.29E-11 | 7.91E-10 |
| CXCL11 | 29.14775 | 2.086842 | -3.80399 | 5.32E-26 | 8.92E-24 |
| CACNA1D | 0.354561 | 1.346936 | 1.925577 | 1.37E-20 | 7.93E-19 |
| SPI1 | 15.59952 | 5.60755 | -1.47606 | 2.82E-18 | 1.07E-16 |
| S100A5 | 1.450352 | 3.484198 | 1.264423 | 8.64E-16 | 1.91E-14 |
| JAML | 1.634982 | 0.712858 | -1.19759 | 2.71E-14 | 4.37E-13 |
| HLA-DRB1 | 484.6612 | 158.1435 | -1.61574 | 2.31E-20 | 1.27E-18 |
| KLK6 | 25.38566 | 5.424368 | -2.22649 | 3.19E-08 | 1.72E-07 |
| LAG3 | 6.146128 | 1.04982 | -2.54954 | 9.12E-24 | 9.91E-22 |
| ITGA3 | 77.57546 | 32.90998 | -1.23708 | 6.54E-19 | 2.79E-17 |
| FAM222A | 0.739747 | 1.559855 | 1.076309 | 3.61E-06 | 1.35E-05 |
| DLX5 | 2.731454 | 6.819924 | 1.320087 | 8.91E-05 | 0.00025 |
| MIR2117HG | 0.727822 | 0.363411 | -1.00198 | 0.000348 | 0.000863 |
| CTXND1 | 0.153906 | 1.164589 | 2.919703 | 8.15E-12 | 8.41E-11 |
| B4GALNT4 | 3.099104 | 7.028307 | 1.181326 | 3.44E-06 | 1.29E-05 |
| NAPSB | 5.642667 | 2.050049 | -1.46072 | 3.61E-06 | 1.35E-05 |
| PCP2 | 1.136829 | 2.655256 | 1.223836 | 4.04E-12 | 4.37E-11 |
| SCUBE3 | 0.517429 | 1.083871 | 1.06676 | 6.43E-06 | 2.27E-05 |
| HCAR1 | 2.7208 | 5.44581 | 1.001116 | 4.53E-10 | 3.41E-09 |
| SERPINB4 | 26.99331 | 3.414679 | -2.98278 | 1.83E-23 | 1.90E-21 |
| SIRPA | 12.8754 | 5.557509 | -1.21211 | 6.78E-16 | 1.53E-14 |
| LIPH | 5.470782 | 11.73385 | 1.100857 | 4.36E-06 | 1.59E-05 |
| TAGAP | 1.967209 | 0.645474 | -1.60772 | 6.23E-20 | 3.20E-18 |
| TNFRSF18 | 8.643253 | 2.93912 | -1.55619 | 3.06E-20 | 1.65E-18 |
| TLR1 | 1.297782 | 0.537572 | -1.27152 | 3.15E-21 | 2.12E-19 |
| LINC01541 | 0.187113 | 1.055276 | 2.495635 | 2.29E-13 | 3.14E-12 |
| WAS | 7.345077 | 2.609808 | -1.49283 | 2.14E-15 | 4.27E-14 |
| KRT20 | 35.49677 | 150.8042 | 2.086917 | 9.61E-20 | 4.79E-18 |
| TUBA4A | 31.10469 | 15.3585 | -1.0181 | 1.79E-19 | 8.52E-18 |
| SYNGR1 | 2.568563 | 5.928161 | 1.206623 | 2.40E-18 | 9.25E-17 |
| CASQ1 | 1.89346 | 6.742946 | 1.832354 | 1.82E-14 | 3.05E-13 |
| CLCF1 | 9.174205 | 4.379523 | -1.06681 | 2.32E-15 | 4.59E-14 |
| IL17D | 0.37216 | 0.820027 | 1.139749 | 1.15E-15 | 2.46E-14 |
| B4GALNT1 | 1.140241 | 0.556788 | -1.03414 | 4.12E-07 | 1.81E-06 |
| PMEL | 0.810557 | 1.928765 | 1.250691 | 3.59E-19 | 1.60E-17 |
| SPRR2A | 60.21361 | 12.49879 | -2.2683 | 3.28E-06 | 1.23E-05 |
| CA2 | 25.03821 | 8.151202 | -1.61905 | 0.00062 | 0.001439 |
| ID4 | 5.871258 | 18.80741 | 1.67956 | 1.41E-11 | 1.39E-10 |
| CCL22 | 4.565905 | 2.080184 | -1.13419 | 2.40E-08 | 1.33E-07 |
| TAP2 | 15.88767 | 5.258808 | -1.5951 | 4.26E-42 | 1.19E-38 |
| CPNE8 | 2.843501 | 1.233016 | -1.20548 | 3.26E-11 | 2.99E-10 |
| PPBP | 2.626988 | 0.460275 | -2.51284 | 0.014619 | 0.024392 |
| TLR2 | 6.967993 | 2.396179 | -1.54001 | 1.81E-30 | 6.46E-28 |
| APOBEC3A | 5.278129 | 1.470424 | -1.84379 | 1.99E-08 | 1.11E-07 |
| MPEG1 | 7.048899 | 2.618624 | -1.42859 | 5.90E-13 | 7.46E-12 |
| SH3RF2 | 2.439084 | 1.095582 | -1.15464 | 7.86E-16 | 1.75E-14 |
| CYP2C18 | 0.51005 | 1.636382 | 1.681798 | 0.026667 | 0.041499 |
| PARM1 | 4.696553 | 10.61633 | 1.176611 | 3.39E-07 | 1.52E-06 |
| ACY3 | 1.667888 | 0.443765 | -1.91016 | 2.74E-25 | 3.85E-23 |
| RHOU | 8.524399 | 26.92234 | 1.659134 | 5.92E-28 | 1.45E-25 |
| REEP6 | 11.36763 | 31.53767 | 1.472144 | 1.26E-21 | 9.45E-20 |
| HLA-DRB6 | 32.43653 | 11.17373 | -1.53751 | 8.22E-17 | 2.30E-15 |
| CEACAM21 | 1.097677 | 0.297776 | -1.88216 | 5.29E-16 | 1.23E-14 |
| SAA2-SAA4 | 3.37085 | 0.309485 | -3.44517 | 4.11E-16 | 9.73E-15 |
| EVA1A | 3.244136 | 1.260621 | -1.3637 | 7.30E-10 | 5.34E-09 |
| SCIMP | 1.07682 | 0.308783 | -1.80211 | 1.35E-12 | 1.60E-11 |
| VIPR1 | 2.007796 | 4.896908 | 1.286259 | 2.05E-16 | 5.19E-15 |
| HID1 | 8.014768 | 20.98709 | 1.38877 | 7.46E-26 | 1.18E-23 |
| CP | 12.23274 | 3.87118 | -1.6599 | 7.43E-09 | 4.46E-08 |
| CCL24 | 2.502325 | 0.685667 | -1.86769 | 9.98E-10 | 7.12E-09 |
| GCNT4 | 1.577842 | 4.647554 | 1.558519 | 1.60E-16 | 4.15E-15 |
| ITLN2 | 0.415793 | 1.018827 | 1.292974 | 2.20E-10 | 1.75E-09 |
| AQP7 | 0.457665 | 1.082023 | 1.241368 | 7.17E-15 | 1.29E-13 |
| F3 | 49.7004 | 12.12687 | -2.03505 | 2.08E-16 | 5.24E-15 |
| MRC1 | 5.698178 | 2.334954 | -1.28711 | 1.68E-11 | 1.64E-10 |
| IGHV4-4 | 5.382118 | 1.373749 | -1.97006 | 0.005968 | 0.010979 |
| PPP1R1B | 3.293823 | 8.754612 | 1.41028 | 5.92E-06 | 2.11E-05 |
| PICSAR | 7.699542 | 1.610364 | -2.25739 | 2.95E-09 | 1.93E-08 |
| PGC | 0.168557 | 1.043585 | 2.630244 | 0.014117 | 0.023628 |
| NOD2 | 1.592947 | 0.646617 | -1.30072 | 6.96E-19 | 2.96E-17 |
| UBE2L6 | 85.87566 | 30.51712 | -1.49263 | 9.33E-40 | 1.62E-36 |
| BMX | 0.519024 | 1.134294 | 1.127922 | 0.014751 | 0.024593 |
| PPM1H | 2.116042 | 4.958396 | 1.228505 | 5.70E-18 | 2.01E-16 |
| KRTAP3-1 | 0.029347 | 4.083417 | 7.12044 | 1.28E-09 | 8.89E-09 |
| SCN5A | 0.273963 | 0.861369 | 1.652652 | 3.91E-14 | 6.13E-13 |
| HAS2 | 6.005663 | 2.396598 | -1.32534 | 9.40E-13 | 1.15E-11 |
| CD7 | 6.932571 | 1.702042 | -2.02612 | 1.76E-25 | 2.57E-23 |
| MIR3189 | 4.345992 | 12.44263 | 1.517534 | 3.49E-12 | 3.85E-11 |
| MMP1 | 98.0216 | 40.17805 | -1.28669 | 2.14E-14 | 3.52E-13 |
| ERBB2 | 36.47443 | 86.20367 | 1.240864 | 5.73E-15 | 1.05E-13 |
| MILR1 | 3.071862 | 1.320848 | -1.21765 | 7.43E-16 | 1.67E-14 |
| AIM2 | 24.54546 | 1.751936 | -3.80843 | 5.44E-61 | 7.57E-57 |
| SPAG17 | 0.503399 | 1.076542 | 1.096629 | 0.00021 | 0.000545 |
| GYG2P1 | 1.046291 | 2.292247 | 1.131479 | 0.001255 | 0.002723 |
| DLX3 | 1.276637 | 3.916054 | 1.617053 | 3.54E-05 | 0.000107 |
| RCOR2 | 1.925321 | 4.734808 | 1.298207 | 5.77E-07 | 2.48E-06 |
| TNFSF13B | 4.005673 | 1.110594 | -1.85071 | 1.69E-24 | 2.12E-22 |
| MSN | 71.83444 | 29.12445 | -1.30244 | 1.38E-22 | 1.20E-20 |
| PSMB9 | 44.98399 | 8.881704 | -2.3405 | 3.61E-42 | 1.19E-38 |
| CD2AP-DT | 3.63851 | 7.524467 | 1.048242 | 4.52E-16 | 1.06E-14 |
| NIPAL1 | 3.437703 | 7.666776 | 1.157175 | 1.01E-13 | 1.50E-12 |
| TNF | 1.769274 | 0.784321 | -1.17364 | 3.22E-07 | 1.45E-06 |
| CA4 | 0.489626 | 2.783719 | 2.507262 | 9.58E-18 | 3.25E-16 |
| HLA-E | 372.8995 | 184.1076 | -1.01824 | 5.20E-34 | 3.29E-31 |
| PPP1R16B | 1.598319 | 0.717708 | -1.15509 | 6.53E-07 | 2.78E-06 |
| TRAV13-1 | 0.827059 | 0.249658 | -1.72804 | 1.64E-14 | 2.77E-13 |
| CD70 | 2.171177 | 0.970634 | -1.16148 | 3.36E-14 | 5.35E-13 |
| LINC01767 | 0.303891 | 1.284913 | 2.080042 | 4.33E-16 | 1.02E-14 |
| BLACAT1 | 1.056121 | 0.474076 | -1.15559 | 7.87E-13 | 9.74E-12 |
| TNNC1 | 3.472153 | 13.41908 | 1.950383 | 3.82E-23 | 3.59E-21 |
| C3orf70 | 0.670838 | 1.399161 | 1.060525 | 2.57E-09 | 1.70E-08 |
| RDM1 | 0.913996 | 1.890294 | 1.048351 | 1.48E-12 | 1.75E-11 |
| INHA | 0.57056 | 1.400481 | 1.295471 | 0.001225 | 0.002665 |
| GZMH | 7.636494 | 1.219156 | -2.64703 | 7.05E-21 | 4.26E-19 |
| LASTR | 7.008524 | 2.553894 | -1.45641 | 3.77E-14 | 5.93E-13 |
| CADM1 | 1.519044 | 3.108554 | 1.03308 | 0.012236 | 0.020755 |
| IGHV3-53 | 6.38052 | 2.83976 | -1.1679 | 0.000615 | 0.00143 |
| CAV2 | 15.0367 | 7.515571 | -1.00053 | 9.58E-13 | 1.17E-11 |
| FAM78B | 0.60436 | 2.616834 | 2.114343 | 0.000295 | 0.000743 |
| NCKAP1L | 3.19641 | 1.196542 | -1.41758 | 1.29E-14 | 2.21E-13 |
| FOLR1 | 0.979918 | 5.720756 | 2.545473 | 0.003009 | 0.005966 |
| BTN3A3 | 6.773203 | 2.829101 | -1.25949 | 4.64E-31 | 1.84E-28 |
| TBX2-AS1 | 2.866548 | 14.37329 | 2.326004 | 4.22E-25 | 5.76E-23 |
| DOCK2 | 1.738242 | 0.584269 | -1.57292 | 7.86E-16 | 1.75E-14 |
| IFNWP19 | 3.148807 | 1.416905 | -1.15206 | 2.44E-07 | 1.12E-06 |
| SCARNA13 | 1.371075 | 5.08233 | 1.890182 | 1.19E-06 | 4.82E-06 |
| ACP5 | 37.15474 | 14.13922 | -1.39384 | 1.89E-09 | 1.28E-08 |
| SLC16A1 | 16.8808 | 8.084252 | -1.0622 | 1.15E-13 | 1.68E-12 |
| LCN12 | 0.228227 | 0.715347 | 1.648176 | 2.93E-11 | 2.71E-10 |
| MIOS-DT | 0.78436 | 1.855568 | 1.242272 | 2.59E-22 | 2.15E-20 |
| HLA-DMA | 42.90543 | 20.59196 | -1.05908 | 3.02E-15 | 5.81E-14 |
| CCR1 | 4.683216 | 2.196569 | -1.09225 | 4.14E-09 | 2.61E-08 |
| TMEM52 | 0.922196 | 2.076437 | 1.170966 | 4.74E-10 | 3.54E-09 |
| MNDA | 4.970958 | 1.405725 | -1.82221 | 2.00E-19 | 9.42E-18 |
| LINC02615 | 0.569945 | 1.183155 | 1.053744 | 3.61E-14 | 5.70E-13 |
| GPC3 | 3.281794 | 18.62481 | 2.504669 | 2.67E-12 | 3.01E-11 |
| IGLV6-57 | 37.38724 | 15.49574 | -1.27067 | 0.000399 | 0.000975 |
| MT2A | 326.2064 | 91.41809 | -1.83523 | 7.36E-18 | 2.56E-16 |
| TBX2 | 9.283358 | 31.34293 | 1.755422 | 2.90E-22 | 2.38E-20 |
| IL1RAP | 3.694168 | 1.252816 | -1.56008 | 3.20E-24 | 3.84E-22 |
| FAM83A-AS1 | 4.759636 | 1.366152 | -1.80073 | 1.36E-20 | 7.88E-19 |
| FKBP5 | 7.464185 | 3.707824 | -1.00941 | 3.24E-15 | 6.17E-14 |
| LILRA5 | 2.542844 | 0.841949 | -1.59464 | 1.31E-14 | 2.25E-13 |
| PPP1R14C | 11.16025 | 4.448424 | -1.327 | 4.39E-09 | 2.75E-08 |
| ICAM1 | 25.21709 | 12.03289 | -1.06742 | 1.27E-16 | 3.39E-15 |
| CLDN11 | 0.848484 | 1.999154 | 1.23643 | 1.25E-09 | 8.70E-09 |
| CXCL8 | 33.40696 | 15.1981 | -1.13626 | 2.08E-13 | 2.91E-12 |
| CD3E | 8.49887 | 2.26172 | -1.90985 | 4.27E-22 | 3.45E-20 |
| KLK5 | 26.19063 | 5.367713 | -2.28667 | 2.04E-06 | 7.94E-06 |
| HLA-DRB5 | 147.0718 | 52.5773 | -1.48401 | 3.62E-18 | 1.32E-16 |
| NPIPB15 | 1.799324 | 4.476049 | 1.314771 | 4.02E-05 | 0.00012 |
| AXIN2 | 1.060957 | 3.480808 | 1.714056 | 3.87E-05 | 0.000116 |
| YPEL1 | 0.373113 | 1.021317 | 1.452747 | 1.25E-16 | 3.35E-15 |
| MMRN2 | 8.748656 | 19.42674 | 1.15091 | 6.34E-11 | 5.57E-10 |
| SRCIN1 | 1.234796 | 2.508762 | 1.022703 | 2.98E-15 | 5.74E-14 |
| DOCK10 | 1.244453 | 0.49522 | -1.32937 | 4.86E-16 | 1.13E-14 |
| CCDC183 | 0.320322 | 0.975811 | 1.607078 | 1.14E-13 | 1.67E-12 |
| TYROBP | 65.09787 | 25.64071 | -1.34417 | 1.92E-16 | 4.92E-15 |
| DUSP7 | 18.98085 | 7.257793 | -1.38694 | 2.34E-27 | 4.93E-25 |
| PLEKHG4B | 0.546966 | 1.254167 | 1.197207 | 0.012118 | 0.020577 |
| IL4I1 | 6.670763 | 2.732073 | -1.28786 | 1.45E-13 | 2.08E-12 |
| ZNF486 | 6.001951 | 14.0487 | 1.226933 | 2.74E-09 | 1.80E-08 |
| SLAMF6 | 2.105144 | 0.576823 | -1.86772 | 8.39E-15 | 1.49E-13 |
| IGHV3-43 | 9.00039 | 3.292551 | -1.45078 | 0.003031 | 0.006001 |
| GSDMC | 7.397431 | 1.681553 | -2.13723 | 3.16E-23 | 3.03E-21 |
| RSAD2 | 8.873488 | 3.889227 | -1.19002 | 4.80E-14 | 7.42E-13 |
| PTHLH | 22.06691 | 5.634627 | -1.96949 | 3.13E-15 | 5.99E-14 |
| CNIH3-AS2 | 0.525467 | 1.305336 | 1.31275 | 7.94E-05 | 0.000225 |
| CCNYL2 | 0.372902 | 0.801458 | 1.103833 | 0.014791 | 0.024641 |
| VGF | 0.518127 | 2.005035 | 1.95225 | 6.75E-06 | 2.38E-05 |
| MZB1 | 10.85243 | 4.292579 | -1.3381 | 7.20E-06 | 2.52E-05 |
| CXCL5 | 9.18635 | 1.554569 | -2.56298 | 1.18E-09 | 8.29E-09 |
| MYH7B | 0.518408 | 2.003836 | 1.950605 | 2.31E-06 | 8.93E-06 |
| ANKRD19P | 1.519005 | 3.297458 | 1.118228 | 2.27E-09 | 1.51E-08 |
| KRT6A | 802.2026 | 124.3289 | -2.68981 | 4.58E-24 | 5.16E-22 |
| PPP1R14D | 2.67095 | 7.276148 | 1.445822 | 2.18E-13 | 3.01E-12 |
| GSTM5 | 0.413613 | 1.278623 | 1.628236 | 6.24E-07 | 2.66E-06 |
| NMRAL2P | 3.897376 | 0.467553 | -3.0593 | 3.48E-13 | 4.58E-12 |
| UPK3B | 47.35493 | 102.7793 | 1.117963 | 1.74E-09 | 1.19E-08 |
| KCNG1 | 4.775578 | 12.15259 | 1.347517 | 2.39E-15 | 4.69E-14 |
| CPAMD8 | 0.867593 | 2.185235 | 1.332697 | 1.19E-11 | 1.20E-10 |
| CLEC4E | 1.184657 | 0.431189 | -1.45808 | 6.25E-13 | 7.85E-12 |
| CRACD | 0.429696 | 0.973419 | 1.179744 | 0.000175 | 0.000461 |
| GNLY | 8.908477 | 1.103665 | -3.01288 | 2.89E-31 | 1.28E-28 |
| ALOX15 | 0.837643 | 2.043317 | 1.286507 | 7.89E-11 | 6.83E-10 |
| FAHD2P1 | 0.863056 | 2.489664 | 1.528426 | 3.50E-07 | 1.57E-06 |
| GATA3-AS1 | 2.962049 | 8.606152 | 1.538773 | 1.92E-16 | 4.93E-15 |
| VNN1 | 1.616613 | 0.454238 | -1.83145 | 1.38E-07 | 6.64E-07 |
| PLAUR | 17.95559 | 8.715521 | -1.04277 | 8.43E-18 | 2.89E-16 |
| SIGLEC7 | 0.873323 | 0.352813 | -1.30761 | 1.91E-14 | 3.17E-13 |
| MIR1915HG | 1.697223 | 3.654068 | 1.106328 | 1.55E-07 | 7.38E-07 |
| ZNF683 | 2.564488 | 0.328122 | -2.96637 | 9.61E-24 | 1.04E-21 |
| HES6 | 2.810522 | 12.39021 | 2.140291 | 7.41E-11 | 6.43E-10 |
| CYP4F23P | 3.374513 | 8.344076 | 1.306073 | 1.37E-14 | 2.34E-13 |
| CD2 | 11.15315 | 2.851621 | -1.9676 | 3.54E-23 | 3.37E-21 |
| ACSBG1 | 0.327828 | 0.661808 | 1.013475 | 2.59E-10 | 2.03E-09 |
| SCARNA21 | 2.055829 | 4.462051 | 1.117987 | 3.03E-08 | 1.64E-07 |
| DNASE1L2 | 0.487541 | 1.001087 | 1.037973 | 6.17E-13 | 7.77E-12 |
| CLEC4A | 2.976883 | 1.018945 | -1.54673 | 1.23E-17 | 4.11E-16 |
| CNIH3 | 0.574184 | 1.206189 | 1.07087 | 3.61E-06 | 1.35E-05 |
| CD300A | 4.604009 | 1.800492 | -1.3545 | 3.77E-19 | 1.66E-17 |
| P2RY13 | 1.231832 | 0.370386 | -1.73371 | 1.56E-14 | 2.64E-13 |
| CD247 | 2.230701 | 0.927832 | -1.26556 | 1.40E-16 | 3.66E-15 |
| IGHV1OR15-2 | 1.393248 | 0.672635 | -1.05056 | 0.001252 | 0.002716 |
| GFRA3 | 0.411101 | 1.616618 | 1.975414 | 2.78E-09 | 1.83E-08 |
| ARHGAP9 | 3.34499 | 0.98896 | -1.75802 | 1.17E-21 | 8.82E-20 |
| ZNF321P | 1.582189 | 3.213916 | 1.02241 | 2.06E-15 | 4.14E-14 |
| FXYD4 | 5.112609 | 31.73698 | 2.634033 | 3.01E-23 | 2.91E-21 |
| L1CAM | 5.183093 | 1.350899 | -1.93989 | 1.17E-06 | 4.77E-06 |
| KRT1 | 58.80707 | 8.926849 | -2.71977 | 0.001818 | 0.003799 |
| MS4A15 | 0.173118 | 2.432529 | 3.812633 | 1.22E-05 | 4.06E-05 |
| OR7E91P | 2.454512 | 5.537082 | 1.173689 | 7.41E-15 | 1.33E-13 |
| GLB1L2 | 1.391615 | 2.802699 | 1.010056 | 2.42E-15 | 4.75E-14 |
| CCL7 | 1.437892 | 0.238228 | -2.59354 | 1.33E-09 | 9.24E-09 |
| CLDN9 | 1.58701 | 3.486702 | 1.135552 | 3.64E-06 | 1.36E-05 |
| IGKV1D-8 | 3.0336 | 1.113162 | -1.44637 | 0.002124 | 0.004365 |
| MOB3B | 3.432128 | 1.486152 | -1.20752 | 8.62E-15 | 1.53E-13 |
| CYTIP | 3.966322 | 1.471666 | -1.43035 | 2.35E-16 | 5.87E-15 |
| SEPTIN1 | 3.319502 | 1.614698 | -1.0397 | 3.82E-06 | 1.42E-05 |
| OAS2 | 28.27339 | 11.76124 | -1.2654 | 2.15E-25 | 3.08E-23 |
| IGHV1-45 | 2.486847 | 0.63196 | -1.97641 | 0.021465 | 0.034259 |
| GPR160 | 4.712168 | 10.14755 | 1.106669 | 3.83E-14 | 6.02E-13 |
| MAP4K1 | 3.368338 | 1.229492 | -1.45398 | 3.47E-09 | 2.23E-08 |
| GBP1P1 | 4.598971 | 1.474334 | -1.64125 | 3.99E-22 | 3.25E-20 |
| AKR1B10 | 88.09743 | 24.31926 | -1.857 | 0.000131 | 0.000353 |
| LINC01139 | 1.043002 | 0.356283 | -1.54965 | 6.09E-07 | 2.60E-06 |
| P2RX5 | 1.144642 | 0.50021 | -1.19429 | 0.000142 | 0.000381 |
| MT1E | 44.16398 | 19.5494 | -1.17575 | 1.29E-07 | 6.28E-07 |
| MAGEB2 | 2.977399 | 0.506582 | -2.55519 | 0.009709 | 0.016927 |
| GPR84 | 1.512749 | 0.453827 | -1.73696 | 3.35E-19 | 1.51E-17 |
| CENPV | 4.467068 | 9.013997 | 1.012839 | 1.10E-08 | 6.44E-08 |
| TRBV6-5 | 0.952833 | 0.279849 | -1.76757 | 1.11E-11 | 1.12E-10 |
| ARHGAP40 | 3.263711 | 7.730429 | 1.244035 | 1.24E-08 | 7.22E-08 |
| AOAH | 2.885967 | 0.827853 | -1.80161 | 5.72E-16 | 1.32E-14 |
| PIP5K1B | 0.348022 | 0.806712 | 1.212874 | 0.000934 | 0.002086 |
| H4C3 | 0.26648 | 1.729131 | 2.697949 | 0.024572 | 0.038716 |
| NFAM1 | 1.901791 | 0.726369 | -1.38858 | 8.39E-15 | 1.49E-13 |
| EPSTI1 | 11.77314 | 5.047759 | -1.22178 | 8.89E-18 | 3.03E-16 |
| FYB1 | 6.675158 | 2.118302 | -1.65589 | 1.40E-25 | 2.10E-23 |
| GZMM | 2.442668 | 1.077487 | -1.18079 | 1.92E-09 | 1.30E-08 |
| CERKL | 1.614097 | 0.785975 | -1.03817 | 5.37E-13 | 6.84E-12 |
| SPRR1B | 190.7466 | 71.91184 | -1.40736 | 8.56E-05 | 0.000241 |
| WDFY4 | 0.86176 | 0.299593 | -1.52428 | 2.21E-09 | 1.47E-08 |
| LINC02154 | 5.084868 | 1.452326 | -1.80785 | 4.64E-08 | 2.44E-07 |
| SP110 | 1.796642 | 0.854777 | -1.07168 | 1.67E-29 | 5.18E-27 |
| UBD | 18.22204 | 3.020969 | -2.5926 | 8.21E-22 | 6.42E-20 |
| TCAP | 0.576646 | 2.746262 | 2.251712 | 4.46E-07 | 1.96E-06 |
| LINC02575 | 0.666685 | 3.183734 | 2.255643 | 1.27E-10 | 1.06E-09 |
| SNCG | 184.9036 | 391.8812 | 1.083643 | 1.10E-12 | 1.33E-11 |
| CD5 | 2.239273 | 1.039715 | -1.10684 | 2.12E-11 | 2.01E-10 |
| SULT2A1 | 0.862743 | 7.70196 | 3.158223 | 9.58E-19 | 3.98E-17 |
| MAN1C1 | 3.212879 | 11.09796 | 1.788356 | 5.06E-14 | 7.78E-13 |
| CYP4F12 | 4.617012 | 9.362778 | 1.019977 | 1.34E-08 | 7.76E-08 |
| PLAC8 | 3.839584 | 1.909103 | -1.00806 | 3.34E-09 | 2.15E-08 |
| LAP3 | 40.7859 | 17.15018 | -1.24985 | 4.35E-26 | 7.56E-24 |
| BATF2 | 8.356402 | 1.674006 | -2.31958 | 5.29E-29 | 1.53E-26 |
| TRBV19 | 0.926092 | 0.302889 | -1.61237 | 2.13E-11 | 2.02E-10 |
| CAV1 | 64.47289 | 26.32864 | -1.29206 | 5.93E-10 | 4.38E-09 |
| IFITM3 | 473.087 | 201.0442 | -1.23459 | 9.50E-20 | 4.75E-18 |
| CXCR3 | 2.342898 | 0.613011 | -1.93431 | 1.54E-16 | 4.03E-15 |
| IGKV1-8 | 5.82507 | 1.698439 | -1.77807 | 0.000149 | 0.0004 |
| CDK6 | 4.317346 | 1.869458 | -1.20752 | 8.56E-22 | 6.62E-20 |
| IGHV4-39 | 68.91672 | 32.20272 | -1.09767 | 9.84E-05 | 0.000274 |
| SCGB1B2P | 0.932031 | 2.163982 | 1.215239 | 2.86E-05 | 8.84E-05 |
| IFI27L2 | 22.22335 | 11.08034 | -1.00407 | 1.63E-21 | 1.18E-19 |
| KLK7 | 6.017014 | 2.063234 | -1.54414 | 3.26E-06 | 1.23E-05 |
| PVALB | 1.701267 | 20.79723 | 3.61171 | 1.83E-12 | 2.11E-11 |
| TRBV5-1 | 0.956553 | 0.284347 | -1.75019 | 3.37E-13 | 4.45E-12 |
| SIT1 | 3.359176 | 0.875479 | -1.93996 | 2.87E-15 | 5.54E-14 |
| PEG10 | 4.720741 | 10.06897 | 1.092831 | 0.025 | 0.039274 |
| DIO3 | 0.495167 | 2.010739 | 2.021737 | 0.018656 | 0.030276 |
| SLAMF8 | 7.903465 | 2.450672 | -1.68931 | 1.19E-18 | 4.87E-17 |
| FBP1 | 36.22315 | 76.62446 | 1.080893 | 7.96E-14 | 1.19E-12 |
| CGN | 9.005009 | 19.41078 | 1.108058 | 3.36E-18 | 1.24E-16 |
| FAT2 | 9.195646 | 2.908298 | -1.66078 | 1.85E-16 | 4.76E-15 |
| RGS20 | 1.867249 | 0.574715 | -1.69999 | 1.08E-16 | 2.96E-15 |
| SLC15A3 | 8.480665 | 3.412022 | -1.31355 | 6.23E-17 | 1.79E-15 |
| BCAT2 | 18.36242 | 40.41271 | 1.138053 | 1.22E-23 | 1.30E-21 |
| HEY1 | 2.922185 | 6.238633 | 1.094182 | 1.15E-09 | 8.08E-09 |
