# Supplementary material for: Identification of a Pyroptosis-Related Gene Signature for Prediction of Overall Survival in Lung Adenocarcinoma
Source: J Oncol. 2021 Sep 30;2021:6365459. doi: 10.1155/2021/6365459 (PMC8497135; doi:10.1155/2021/6365459)
Supplement: Supplementary Materials — Supplementary File Table S1: 52 genes associated with pyroptosis from prior reviews. Supplementary File Table S2: 1458 DEGs associated with cluster 1 and cluster 2. Supplementary File Table S3: 13 genes identified by univariate regression. Supplementary File Table S4: 317 DEGs between low- and high-risk groups in TCGA cohort. Supplementary File Figure S1: An overview of the differential gene expression between the two pyroptosis-related clusters in TCGA cohort. [file 6365459.f1.zip › 6365459.f1/TableS3.docx]

Table S3. 13 genes identified by univariate regression.

| id | HR | HR.95L | HR.95H | pvalue |
| --- | --- | --- | --- | --- |
| GOLT1A | 0.819413 | 0.731678 | 0.917669 | 0.000567 |
| PAQR8 | 0.821183 | 0.716199 | 0.941555 | 0.00476 |
| ACOXL | 0.787379 | 0.665908 | 0.931007 | 0.005171 |
| DSG3 | 1.101959 | 1.028731 | 1.180399 | 0.005651 |
| SPRR2A | 1.09723 | 1.027598 | 1.17158 | 0.005541 |
| KRT1 | 1.128411 | 1.047241 | 1.215871 | 0.001514 |
| SERPINB2 | 1.175236 | 1.081728 | 1.276827 | 0.000135 |
| TNC | 1.142274 | 1.048649 | 1.244258 | 0.002299 |
| DSC2 | 1.163648 | 1.052169 | 1.286937 | 0.003181 |
| SERPINB7 | 1.218669 | 1.071752 | 1.385725 | 0.002551 |
| CD109 | 1.334476 | 1.161567 | 1.533125 | 4.60E-05 |
| ADCY7 | 1.496668 | 1.170227 | 1.914172 | 0.001317 |
| CPT1C | 1.25768 | 1.071209 | 1.476611 | 0.005109 |
