# Supplementary material for: Identification of a Pyroptosis-Related Gene Signature for Prediction of Overall Survival in Lung Adenocarcinoma
Source: J Oncol. 2021 Sep 30;2021:6365459. doi: 10.1155/2021/6365459 (PMC8497135; doi:10.1155/2021/6365459)
Supplement: Supplementary Materials — Supplementary File Table S1: 52 genes associated with pyroptosis from prior reviews. Supplementary File Table S2: 1458 DEGs associated with cluster 1 and cluster 2. Supplementary File Table S3: 13 genes identified by univariate regression. Supplementary File Table S4: 317 DEGs between low- and high-risk groups in TCGA cohort. Supplementary File Figure S1: An overview of the differential gene expression between the two pyroptosis-related clusters in TCGA cohort. [file 6365459.f1.zip › 6365459.f1/TableS4.docx]

Table S4. 317 DEGs between low- and high-risk groups in TCGA cohort.

| gene | lowMean | highMean | logFC | pValue | fdr |
| --- | --- | --- | --- | --- | --- |
| COL1A2 | 7.712505 | 9.067781 | 1.355277 | 2.10E-13 | 2.16E-12 |
| TGFBI | 6.289747 | 7.969076 | 1.679329 | 1.96E-23 | 1.45E-21 |
| HOXB8 | 5.467061 | 4.406301 | -1.06076 | 7.25E-11 | 4.75E-10 |
| CAPN5 | 7.160269 | 5.60256 | -1.55771 | 1.49E-29 | 5.72E-27 |
| COL3A1 | 8.1137 | 9.465066 | 1.351366 | 7.02E-13 | 6.52E-12 |
| TGM1 | 3.152582 | 4.285182 | 1.1326 | 3.39E-16 | 6.07E-15 |
| PLA2G2F | 6.170126 | 4.229995 | -1.94013 | 4.12E-25 | 4.61E-23 |
| GATA2 | 6.202624 | 5.019582 | -1.18304 | 4.61E-20 | 1.69E-18 |
| RHBG | 4.674713 | 3.289497 | -1.38522 | 1.26E-33 | 3.09E-30 |
| TNFAIP2 | 9.202691 | 7.870468 | -1.33222 | 1.53E-22 | 9.50E-21 |
| HMGCS2 | 8.936774 | 5.6294 | -3.30737 | 3.22E-33 | 5.65E-30 |
| CD14 | 5.620488 | 6.850934 | 1.230446 | 4.29E-17 | 9.00E-16 |
| ITGA5 | 5.69847 | 6.928036 | 1.229566 | 1.55E-19 | 5.13E-18 |
| CRTAC1 | 5.915487 | 4.22856 | -1.68693 | 4.04E-17 | 8.58E-16 |
| CYB5A | 7.602495 | 6.36715 | -1.23535 | 4.52E-30 | 2.42E-27 |
| SCUBE2 | 6.235959 | 4.569265 | -1.66669 | 1.27E-23 | 1.01E-21 |
| TMEM97 | 8.876211 | 7.315634 | -1.56058 | 3.76E-26 | 5.36E-24 |
| LAMA3 | 3.986471 | 5.314171 | 1.327701 | 2.26E-18 | 6.14E-17 |
| PPP1R3C | 6.745686 | 5.193838 | -1.55185 | 7.26E-23 | 4.75E-21 |
| SLC14A1 | 6.98153 | 5.042278 | -1.93925 | 7.90E-25 | 7.90E-23 |
| PI3 | 4.081937 | 6.849149 | 2.767212 | 4.61E-18 | 1.17E-16 |
| PPFIBP2 | 7.402628 | 5.836484 | -1.56614 | 7.80E-38 | 3.20E-34 |
| THBS1 | 6.19962 | 7.22176 | 1.02214 | 5.82E-13 | 5.50E-12 |
| GPX2 | 9.973431 | 7.872531 | -2.1009 | 3.47E-16 | 6.18E-15 |
| SPRR2D | 3.045609 | 4.383053 | 1.337444 | 2.30E-13 | 2.35E-12 |
| PPARG | 7.926586 | 6.062189 | -1.8644 | 3.11E-32 | 3.47E-29 |
| TNC | 4.255935 | 6.076504 | 1.82057 | 1.03E-26 | 1.74E-24 |
| BARX2 | 3.159819 | 4.164402 | 1.004583 | 1.62E-14 | 2.05E-13 |
| RBP1 | 4.570619 | 5.585771 | 1.015153 | 2.78E-13 | 2.78E-12 |
| SGK2 | 4.22932 | 3.220491 | -1.00883 | 4.88E-25 | 5.32E-23 |
| CLDN23 | 6.497051 | 5.462246 | -1.0348 | 2.85E-19 | 9.12E-18 |
| HES2 | 3.517188 | 4.607009 | 1.089821 | 3.05E-15 | 4.42E-14 |
| IFI27 | 6.524577 | 7.632658 | 1.108081 | 1.91E-08 | 8.18E-08 |
| KRT17 | 9.856125 | 10.85959 | 1.003466 | 3.74E-07 | 1.28E-06 |
| CD44 | 6.726213 | 7.962744 | 1.236531 | 6.24E-21 | 2.73E-19 |
| TM4SF1 | 6.813928 | 7.953945 | 1.140016 | 5.53E-13 | 5.26E-12 |
| FOXA1 | 7.852676 | 6.233495 | -1.61918 | 6.67E-23 | 4.41E-21 |
| S100A7 | 3.797434 | 6.022904 | 2.22547 | 5.59E-12 | 4.40E-11 |
| CXCL1 | 4.937895 | 6.172373 | 1.234479 | 9.39E-12 | 7.13E-11 |
| PNCK | 6.030913 | 4.866542 | -1.16437 | 2.89E-11 | 2.02E-10 |
| KRT14 | 4.226788 | 6.848214 | 2.621426 | 3.82E-15 | 5.45E-14 |
| SFRP2 | 4.576399 | 6.332569 | 1.75617 | 1.05E-12 | 9.46E-12 |
| FCER1G | 5.408335 | 6.449586 | 1.041251 | 1.45E-13 | 1.52E-12 |
| SCCPDH | 8.157251 | 7.035753 | -1.1215 | 3.29E-22 | 1.85E-20 |
| GFPT2 | 3.462452 | 4.573551 | 1.111099 | 1.08E-20 | 4.47E-19 |
| BHMT | 6.074736 | 4.210745 | -1.86399 | 6.39E-24 | 5.35E-22 |
| CYP4B1 | 7.903166 | 5.539166 | -2.364 | 3.80E-26 | 5.36E-24 |
| FGFBP1 | 3.612312 | 5.687165 | 2.074853 | 4.33E-19 | 1.34E-17 |
| PDPN | 5.035791 | 6.127344 | 1.091552 | 6.09E-16 | 1.02E-14 |
| FOXQ1 | 8.59901 | 7.099899 | -1.49911 | 7.66E-17 | 1.54E-15 |
| CAB39L | 5.914248 | 4.710587 | -1.20366 | 5.24E-29 | 1.65E-26 |
| SLC9A2 | 5.053731 | 4.036416 | -1.01731 | 6.91E-22 | 3.66E-20 |
| GSTM1 | 5.562916 | 4.528351 | -1.03456 | 7.45E-05 | 0.000172 |
| CDH3 | 5.605257 | 6.766799 | 1.161542 | 2.78E-13 | 2.78E-12 |
| BNC1 | 2.657112 | 3.833244 | 1.176132 | 5.02E-25 | 5.41E-23 |
| FER1L4 | 6.402088 | 4.206458 | -2.19563 | 2.75E-38 | 1.69E-34 |
| CYP4F8 | 4.995439 | 3.441002 | -1.55444 | 1.71E-24 | 1.63E-22 |
| IGF2 | 7.597572 | 6.366044 | -1.23153 | 0.000317 | 0.000662 |
| DSC2 | 3.953838 | 5.31874 | 1.364902 | 1.40E-23 | 1.10E-21 |
| SPINK1 | 10.44567 | 6.62744 | -3.81823 | 1.42E-29 | 5.63E-27 |
| AKR1C3 | 8.038039 | 6.911562 | -1.12648 | 2.58E-11 | 1.82E-10 |
| IDH1 | 7.931793 | 6.713898 | -1.21789 | 1.66E-22 | 1.02E-20 |
| GATA3 | 8.579856 | 6.623555 | -1.9563 | 9.99E-30 | 4.39E-27 |
| UPK3A | 7.336688 | 4.657292 | -2.6794 | 4.40E-27 | 8.45E-25 |
| KRT8 | 10.51705 | 9.396928 | -1.12012 | 7.68E-16 | 1.26E-14 |
| EHD2 | 5.922982 | 6.96798 | 1.044999 | 7.76E-22 | 4.04E-20 |
| RAB15 | 7.340857 | 5.969858 | -1.371 | 8.48E-29 | 2.61E-26 |
| S100A8 | 6.235096 | 8.68245 | 2.447354 | 1.91E-15 | 2.89E-14 |
| SRPX | 4.300845 | 5.51991 | 1.219064 | 1.83E-14 | 2.29E-13 |
| POF1B | 5.642288 | 4.429237 | -1.21305 | 1.53E-20 | 6.15E-19 |
| ERBB3 | 7.285899 | 6.259609 | -1.02629 | 5.57E-20 | 2.02E-18 |
| MMP9 | 4.451451 | 5.785266 | 1.333815 | 2.35E-15 | 3.51E-14 |
| FAM3D | 5.745902 | 4.27118 | -1.47472 | 4.50E-20 | 1.65E-18 |
| ACOXL | 4.478602 | 3.462184 | -1.01642 | 1.14E-28 | 3.27E-26 |
| IL20RB | 3.80693 | 5.046476 | 1.239546 | 5.61E-14 | 6.40E-13 |
| TNFRSF21 | 9.840584 | 8.799224 | -1.04136 | 2.34E-20 | 9.02E-19 |
| CDA | 3.743393 | 4.872591 | 1.129198 | 3.86E-16 | 6.77E-15 |
| TESC | 6.839482 | 5.048158 | -1.79132 | 2.14E-22 | 1.27E-20 |
| SLC7A5 | 6.678703 | 7.691193 | 1.01249 | 4.92E-14 | 5.70E-13 |
| LUM | 7.04075 | 8.112878 | 1.072129 | 4.58E-08 | 1.84E-07 |
| ICA1 | 7.164858 | 6.152972 | -1.01189 | 3.31E-26 | 4.85E-24 |
| KLK10 | 3.123863 | 4.364575 | 1.240712 | 2.86E-21 | 1.33E-19 |
| RHPN1 | 5.788093 | 4.758238 | -1.02985 | 1.36E-19 | 4.59E-18 |
| BTG2 | 8.596379 | 7.303382 | -1.293 | 5.67E-24 | 4.78E-22 |
| HSD17B2 | 6.203722 | 4.849588 | -1.35413 | 3.18E-17 | 6.89E-16 |
| SEMA5A | 5.849221 | 4.642083 | -1.20714 | 5.59E-19 | 1.69E-17 |
| TRAK1 | 7.425689 | 6.215201 | -1.21049 | 4.25E-33 | 6.54E-30 |
| S100A2 | 7.218891 | 8.247515 | 1.028624 | 9.27E-06 | 2.51E-05 |
| ATF7IP2 | 5.804781 | 4.772139 | -1.03264 | 1.10E-27 | 2.56E-25 |
| DSC3 | 3.877809 | 5.536488 | 1.658679 | 1.53E-15 | 2.36E-14 |
| MMP12 | 4.280743 | 5.396105 | 1.115362 | 1.42E-10 | 8.83E-10 |
| CYP3A5 | 5.983449 | 4.638764 | -1.34468 | 6.85E-20 | 2.45E-18 |
| GBP6 | 3.294507 | 4.300658 | 1.006151 | 1.16E-13 | 1.25E-12 |
| MMP11 | 4.774946 | 6.015008 | 1.240062 | 4.61E-13 | 4.43E-12 |
| GDPD3 | 6.240983 | 5.145097 | -1.09589 | 3.42E-13 | 3.39E-12 |
| FABP6 | 5.993289 | 4.706943 | -1.28635 | 1.32E-14 | 1.71E-13 |
| FN1 | 5.377502 | 6.945728 | 1.568227 | 2.24E-18 | 6.10E-17 |
| UCHL1 | 3.969175 | 5.227131 | 1.257956 | 3.73E-14 | 4.43E-13 |
| ALOX5 | 7.528703 | 6.250159 | -1.27854 | 1.86E-23 | 1.39E-21 |
| NXN | 5.192587 | 6.232942 | 1.040354 | 8.14E-20 | 2.88E-18 |
| SHROOM1 | 6.091069 | 5.011728 | -1.07934 | 6.05E-25 | 6.41E-23 |
| OXCT1 | 4.184386 | 5.211161 | 1.026775 | 2.29E-21 | 1.09E-19 |
| VGLL1 | 7.330628 | 5.668676 | -1.66195 | 9.94E-17 | 1.95E-15 |
| ISLR | 4.716271 | 5.776707 | 1.060436 | 9.30E-10 | 4.99E-09 |
| PTGS1 | 4.235446 | 5.349648 | 1.114202 | 3.38E-14 | 4.03E-13 |
| LGALS1 | 8.919642 | 10.02428 | 1.104637 | 2.55E-15 | 3.77E-14 |
| TM7SF2 | 6.895758 | 5.827548 | -1.06821 | 6.03E-19 | 1.81E-17 |
| C1S | 5.781259 | 7.039236 | 1.257977 | 1.12E-14 | 1.47E-13 |
| SLC30A2 | 5.298938 | 3.798048 | -1.50089 | 2.45E-22 | 1.44E-20 |
| CALML5 | 3.787886 | 5.048778 | 1.260892 | 2.79E-08 | 1.16E-07 |
| CXCL10 | 5.088755 | 6.463667 | 1.374912 | 5.67E-11 | 3.78E-10 |
| AEBP1 | 6.23635 | 7.545062 | 1.308711 | 7.57E-12 | 5.85E-11 |
| PDZK1IP1 | 4.232893 | 5.292076 | 1.059182 | 1.43E-07 | 5.23E-07 |
| VIM | 7.618103 | 8.6289 | 1.010797 | 1.51E-11 | 1.11E-10 |
| DHRS2 | 9.620967 | 6.45484 | -3.16613 | 4.49E-29 | 1.45E-26 |
| C10orf99 | 7.763716 | 5.922882 | -1.84083 | 2.43E-13 | 2.47E-12 |
| S100A9 | 8.137488 | 9.6974 | 1.559912 | 2.72E-09 | 1.33E-08 |
| INA | 6.053313 | 4.654379 | -1.39893 | 7.53E-15 | 1.02E-13 |
| ANXA10 | 5.799716 | 4.667058 | -1.13266 | 2.78E-06 | 8.22E-06 |
| SULF2 | 4.51921 | 5.872904 | 1.353694 | 6.50E-21 | 2.83E-19 |
| COL6A3 | 6.257677 | 7.375401 | 1.117724 | 2.19E-12 | 1.87E-11 |
| NT5E | 4.123016 | 5.155073 | 1.032057 | 5.53E-14 | 6.34E-13 |
| CD109 | 3.241605 | 4.683988 | 1.442383 | 3.61E-41 | 4.44E-37 |
| BAMBI | 8.108203 | 6.417517 | -1.69069 | 2.35E-23 | 1.71E-21 |
| MT1X | 5.536889 | 7.164987 | 1.628097 | 1.25E-26 | 2.03E-24 |
| EEF1A2 | 6.716733 | 5.668685 | -1.04805 | 7.32E-07 | 2.39E-06 |
| MSX2 | 6.33546 | 5.063879 | -1.27158 | 1.28E-21 | 6.40E-20 |
| TBX1 | 5.239843 | 4.1568 | -1.08304 | 9.14E-14 | 1.00E-12 |
| OSMR | 3.903597 | 5.110818 | 1.207222 | 2.26E-26 | 3.47E-24 |
| BCAS1 | 6.54101 | 4.823846 | -1.71716 | 4.39E-25 | 4.82E-23 |
| KCNN4 | 7.378552 | 6.341959 | -1.03659 | 3.67E-13 | 3.61E-12 |
| SAA1 | 3.828867 | 5.141984 | 1.313116 | 2.29E-09 | 1.14E-08 |
| KRT16 | 4.913444 | 6.908904 | 1.99546 | 1.50E-14 | 1.93E-13 |
| VSNL1 | 3.036139 | 4.158675 | 1.122536 | 2.16E-25 | 2.48E-23 |
| C1QB | 6.412352 | 7.682462 | 1.27011 | 2.07E-12 | 1.77E-11 |
| PRNP | 7.58692 | 8.74206 | 1.15514 | 3.45E-21 | 1.59E-19 |
| RHCG | 3.597565 | 4.903429 | 1.305863 | 5.28E-11 | 3.53E-10 |
| COL5A2 | 5.898368 | 7.143288 | 1.24492 | 2.44E-17 | 5.41E-16 |
| METTL7A | 7.667295 | 6.177695 | -1.4896 | 7.20E-23 | 4.73E-21 |
| SLPI | 6.802204 | 8.337244 | 1.53504 | 4.09E-11 | 2.78E-10 |
| CNGA1 | 5.186669 | 3.965425 | -1.22124 | 1.65E-25 | 1.93E-23 |
| GPRC5C | 5.785951 | 4.608968 | -1.17698 | 2.91E-23 | 2.07E-21 |
| ST3GAL4 | 7.39036 | 6.349334 | -1.04103 | 3.45E-18 | 9.01E-17 |
| ELF3 | 8.919914 | 7.466628 | -1.45329 | 2.08E-20 | 8.05E-19 |
| EFEMP1 | 3.924681 | 5.381401 | 1.45672 | 1.98E-20 | 7.72E-19 |
| SERPINB5 | 5.982598 | 7.129765 | 1.147167 | 7.99E-12 | 6.14E-11 |
| SPHK1 | 3.735707 | 5.070229 | 1.334522 | 7.75E-30 | 3.66E-27 |
| CHI3L1 | 3.987492 | 4.997953 | 1.01046 | 3.64E-11 | 2.50E-10 |
| FGFR3 | 8.296117 | 7.100132 | -1.19599 | 1.79E-09 | 9.12E-09 |
| KRT6B | 2.872858 | 5.339246 | 2.466388 | 1.19E-23 | 9.51E-22 |
| PSCA | 10.49451 | 7.2516 | -3.24291 | 4.11E-24 | 3.69E-22 |
| SCNN1G | 6.250526 | 4.447313 | -1.80321 | 4.99E-26 | 6.75E-24 |
| CXCL9 | 4.263034 | 5.335921 | 1.072887 | 1.15E-09 | 6.04E-09 |
| EMP3 | 5.290519 | 6.568815 | 1.278297 | 1.81E-20 | 7.16E-19 |
| C1QA | 6.850253 | 8.090951 | 1.240698 | 5.07E-12 | 4.03E-11 |
| TAC3 | 4.458592 | 3.294805 | -1.16379 | 5.69E-15 | 7.84E-14 |
| KRT6C | 2.748156 | 4.237765 | 1.48961 | 2.84E-21 | 1.32E-19 |
| S100A10 | 8.690401 | 9.939801 | 1.249399 | 7.41E-20 | 2.63E-18 |
| TSPAN6 | 7.692862 | 6.652258 | -1.0406 | 6.79E-25 | 7.14E-23 |
| CXADR | 7.692312 | 6.684153 | -1.00816 | 5.37E-17 | 1.11E-15 |
| NDRG2 | 6.681757 | 5.676425 | -1.00533 | 3.53E-18 | 9.18E-17 |
| NMU | 3.881538 | 4.945299 | 1.063761 | 1.51E-14 | 1.94E-13 |
| DSG3 | 2.782655 | 5.015195 | 2.23254 | 5.77E-28 | 1.39E-25 |
| FAM3B | 5.769341 | 3.884359 | -1.88498 | 7.16E-30 | 3.52E-27 |
| SRGN | 6.489347 | 7.535684 | 1.046336 | 1.53E-11 | 1.13E-10 |
| EMP1 | 5.800563 | 7.081788 | 1.281225 | 2.60E-23 | 1.87E-21 |
| SPP1 | 5.786587 | 7.184977 | 1.39839 | 4.82E-14 | 5.61E-13 |
| SCNN1B | 6.894419 | 5.209909 | -1.68451 | 1.26E-19 | 4.28E-18 |
| GOLT1A | 5.538478 | 4.025331 | -1.51315 | 2.34E-30 | 1.37E-27 |
| SYT8 | 7.307133 | 6.115112 | -1.19202 | 5.77E-08 | 2.27E-07 |
| FBLN1 | 8.872735 | 7.778563 | -1.09417 | 3.12E-12 | 2.58E-11 |
| CPA4 | 2.858107 | 4.020305 | 1.162198 | 2.39E-24 | 2.23E-22 |
| CTSH | 8.505999 | 7.25989 | -1.24611 | 6.99E-24 | 5.73E-22 |
| AGR2 | 7.529907 | 6.172826 | -1.35708 | 1.31E-10 | 8.20E-10 |
| COL1A1 | 8.256956 | 9.634468 | 1.377513 | 1.10E-13 | 1.19E-12 |
| UPK1B | 8.283828 | 6.635732 | -1.6481 | 3.30E-09 | 1.59E-08 |
| ANXA1 | 7.26524 | 8.765606 | 1.500367 | 3.58E-22 | 1.98E-20 |
| TMEM45B | 6.765636 | 5.373078 | -1.39256 | 6.20E-18 | 1.53E-16 |
| CASP14 | 3.646501 | 4.669812 | 1.023311 | 1.99E-05 | 5.09E-05 |
| SERPINB13 | 3.059083 | 4.485318 | 1.426235 | 1.05E-17 | 2.46E-16 |
| LOX | 3.532658 | 4.539831 | 1.007173 | 2.09E-24 | 1.96E-22 |
| ACSL5 | 7.710804 | 6.32612 | -1.38468 | 1.08E-25 | 1.33E-23 |
| GPR68 | 3.678187 | 4.714141 | 1.035954 | 1.07E-21 | 5.45E-20 |
| C1QC | 6.289004 | 7.539611 | 1.250608 | 2.76E-13 | 2.77E-12 |
| GDF15 | 8.353289 | 6.698057 | -1.65523 | 2.54E-16 | 4.68E-15 |
| TUBB6 | 4.945755 | 6.58945 | 1.643695 | 2.83E-29 | 9.39E-27 |
| CYP2J2 | 6.898482 | 5.574891 | -1.32359 | 3.68E-21 | 1.69E-19 |
| ECM1 | 4.426392 | 5.45714 | 1.030749 | 1.44E-23 | 1.11E-21 |
| VSIG2 | 9.380571 | 6.633945 | -2.74663 | 1.30E-32 | 1.60E-29 |
| TMC4 | 7.408162 | 6.1974 | -1.21076 | 3.29E-22 | 1.85E-20 |
| SNAI2 | 4.803118 | 5.965189 | 1.162071 | 1.83E-20 | 7.20E-19 |
| S100P | 11.34458 | 9.389085 | -1.95549 | 2.97E-20 | 1.12E-18 |
| CTSE | 5.59063 | 4.03181 | -1.55882 | 2.97E-18 | 7.83E-17 |
| SLC44A3 | 6.929704 | 5.738791 | -1.19091 | 2.84E-26 | 4.20E-24 |
| IFITM1 | 7.158515 | 8.355836 | 1.197321 | 2.35E-15 | 3.50E-14 |
| GJB2 | 6.413379 | 7.752921 | 1.339542 | 2.84E-10 | 1.67E-09 |
| FAM83A | 4.751223 | 5.796487 | 1.045265 | 1.94E-08 | 8.29E-08 |
| DEGS2 | 4.993083 | 3.851265 | -1.14182 | 6.85E-22 | 3.65E-20 |
| BMP3 | 4.915146 | 3.754091 | -1.16105 | 9.16E-19 | 2.63E-17 |
| INHBA | 3.587086 | 4.621515 | 1.034429 | 2.74E-21 | 1.29E-19 |
| UPK1A | 9.393278 | 6.319593 | -3.07369 | 1.15E-24 | 1.11E-22 |
| DENND2D | 7.014898 | 5.984875 | -1.03002 | 7.13E-27 | 1.25E-24 |
| CRH | 4.902783 | 3.529618 | -1.37317 | 2.57E-15 | 3.79E-14 |
| KRT4 | 3.73998 | 4.845988 | 1.106008 | 8.30E-07 | 2.68E-06 |
| UCP2 | 8.051505 | 6.819384 | -1.23212 | 1.28E-23 | 1.01E-21 |
| FCRLB | 5.650258 | 4.444846 | -1.20541 | 1.74E-19 | 5.70E-18 |
| KRT5 | 5.861816 | 8.766187 | 2.90437 | 4.86E-19 | 1.49E-17 |
| RAB31 | 6.117354 | 7.21427 | 1.096916 | 1.74E-23 | 1.31E-21 |
| UPK2 | 9.441958 | 6.14026 | -3.3017 | 2.87E-27 | 5.78E-25 |
| GRHL3 | 6.652047 | 5.202065 | -1.44998 | 4.19E-17 | 8.83E-16 |
| SPRR2E | 2.779314 | 3.974674 | 1.19536 | 2.66E-15 | 3.91E-14 |
| GGT6 | 7.103345 | 5.386834 | -1.71651 | 5.06E-26 | 6.77E-24 |
| COL6A2 | 6.576284 | 7.914925 | 1.338641 | 1.14E-15 | 1.82E-14 |
| TMEM45A | 3.975412 | 5.438329 | 1.462917 | 9.18E-26 | 1.15E-23 |
| BTBD16 | 7.094424 | 5.061933 | -2.03249 | 7.31E-21 | 3.13E-19 |
| SORL1 | 6.076445 | 5.004518 | -1.07193 | 1.37E-19 | 4.61E-18 |
| SERPINB3 | 3.649122 | 5.361097 | 1.711975 | 2.32E-14 | 2.84E-13 |
| CLIC4 | 5.259374 | 6.369396 | 1.110022 | 2.71E-26 | 4.06E-24 |
| SSH3 | 8.224194 | 7.103255 | -1.12094 | 4.23E-25 | 4.69E-23 |
| FCGR3A | 4.282412 | 5.385328 | 1.102916 | 6.71E-15 | 9.13E-14 |
| IFI16 | 6.005506 | 7.106053 | 1.100547 | 8.30E-17 | 1.65E-15 |
| COL6A1 | 7.021424 | 8.424126 | 1.402702 | 2.59E-17 | 5.70E-16 |
| LAMC2 | 5.259143 | 6.576739 | 1.317596 | 3.34E-14 | 3.99E-13 |
| CCR7 | 5.354398 | 4.163416 | -1.19098 | 6.41E-10 | 3.53E-09 |
| TIMP2 | 6.106676 | 7.244841 | 1.138165 | 2.32E-14 | 2.84E-13 |
| B3GNT3 | 6.696213 | 5.646007 | -1.05021 | 5.32E-14 | 6.11E-13 |
| KLHDC7A | 4.744478 | 3.266085 | -1.47839 | 5.22E-30 | 2.68E-27 |
| TMPRSS2 | 7.127803 | 5.123876 | -2.00393 | 5.34E-28 | 1.31E-25 |
| PLAU | 6.843474 | 7.988394 | 1.14492 | 2.13E-15 | 3.21E-14 |
| NNMT | 5.189914 | 6.469541 | 1.279627 | 1.15E-12 | 1.03E-11 |
| MAOA | 8.198672 | 6.887336 | -1.31134 | 3.41E-19 | 1.07E-17 |
| SERPINB2 | 2.896962 | 4.56347 | 1.666509 | 1.37E-29 | 5.61E-27 |
| IGF2BP2 | 4.539481 | 6.073197 | 1.533717 | 2.77E-29 | 9.39E-27 |
| SLC38A5 | 4.065706 | 5.116427 | 1.050721 | 3.86E-14 | 4.56E-13 |
| CEBPA | 7.282908 | 6.142309 | -1.1406 | 1.32E-18 | 3.72E-17 |
| HTRA3 | 4.301 | 5.524984 | 1.223984 | 5.18E-17 | 1.07E-15 |
| SULF1 | 4.265711 | 5.308661 | 1.04295 | 2.94E-12 | 2.44E-11 |
| COL17A1 | 5.143501 | 6.395303 | 1.251801 | 1.81E-08 | 7.79E-08 |
| TLE2 | 6.014814 | 4.746196 | -1.26862 | 6.02E-23 | 4.02E-21 |
| CXCL11 | 3.746618 | 4.846001 | 1.099383 | 3.15E-11 | 2.19E-10 |
| FLNC | 4.492665 | 5.537113 | 1.044448 | 7.62E-12 | 5.88E-11 |
| KLK6 | 2.988499 | 4.131406 | 1.142907 | 5.85E-14 | 6.65E-13 |
| POSTN | 4.745621 | 6.188017 | 1.442396 | 5.95E-14 | 6.75E-13 |
| TJP3 | 6.123979 | 5.044808 | -1.07917 | 2.01E-16 | 3.77E-15 |
| SERPINB4 | 3.286784 | 4.651005 | 1.364221 | 8.39E-16 | 1.37E-14 |
| SIRPA | 5.214554 | 6.281344 | 1.066791 | 1.84E-20 | 7.23E-19 |
| KRT20 | 7.226099 | 5.004427 | -2.22167 | 5.23E-15 | 7.25E-14 |
| CASQ1 | 5.074311 | 3.760439 | -1.31387 | 1.03E-24 | 1.00E-22 |
| ENTPD3 | 5.825432 | 4.752912 | -1.07252 | 1.10E-14 | 1.44E-13 |
| CROT | 7.300366 | 6.222765 | -1.0776 | 9.10E-25 | 9.03E-23 |
| ID1 | 9.723873 | 8.55959 | -1.16428 | 7.41E-16 | 1.23E-14 |
| SPRR2A | 2.944678 | 4.56701 | 1.622332 | 1.72E-14 | 2.17E-13 |
| FLNA | 6.90384 | 8.194438 | 1.290598 | 1.30E-21 | 6.48E-20 |
| ANXA5 | 7.305344 | 8.407879 | 1.102535 | 6.38E-27 | 1.17E-24 |
| RHOU | 7.4987 | 6.101775 | -1.39692 | 9.81E-24 | 7.94E-22 |
| REEP6 | 7.016597 | 5.416833 | -1.59976 | 4.65E-27 | 8.81E-25 |
| VIPR1 | 5.743799 | 4.44994 | -1.29386 | 1.27E-29 | 5.40E-27 |
| SBSN | 3.262516 | 4.67213 | 1.409614 | 2.06E-09 | 1.03E-08 |
| F3 | 4.578103 | 6.220688 | 1.642584 | 1.16E-23 | 9.30E-22 |
| IGFBP6 | 5.439645 | 6.625295 | 1.18565 | 5.19E-13 | 4.97E-12 |
| MYC | 6.092549 | 7.251241 | 1.158691 | 4.36E-19 | 1.34E-17 |
| MMP1 | 5.22678 | 6.477714 | 1.250933 | 8.58E-09 | 3.86E-08 |
| MSN | 6.674729 | 7.958316 | 1.283587 | 7.83E-23 | 5.04E-21 |
| TNNC1 | 5.348489 | 4.183845 | -1.16464 | 5.67E-18 | 1.42E-16 |
| DEGS1 | 5.884008 | 6.995237 | 1.111229 | 3.33E-27 | 6.50E-25 |
| PALLD | 5.718212 | 6.912211 | 1.194 | 5.37E-19 | 1.63E-17 |
| CAV2 | 4.760869 | 5.761949 | 1.00108 | 1.89E-19 | 6.12E-18 |
| SLC16A1 | 3.688471 | 5.21308 | 1.524609 | 2.16E-31 | 1.77E-28 |
| IGFBP3 | 9.955789 | 8.905778 | -1.05001 | 7.06E-10 | 3.87E-09 |
| GAS1 | 3.579846 | 4.674336 | 1.094489 | 2.12E-17 | 4.74E-16 |
| MT2A | 7.142534 | 9.134595 | 1.992061 | 6.10E-26 | 7.90E-24 |
| TBX2 | 7.807751 | 5.986493 | -1.82126 | 4.76E-28 | 1.25E-25 |
| TNNI2 | 6.567112 | 5.408401 | -1.15871 | 1.38E-07 | 5.06E-07 |
| PPP1R14C | 3.585664 | 4.852276 | 1.266611 | 5.52E-16 | 9.39E-15 |
| ICAM1 | 4.644008 | 5.705246 | 1.061238 | 1.69E-17 | 3.84E-16 |
| KLK5 | 2.887413 | 4.088142 | 1.200729 | 8.79E-19 | 2.54E-17 |
| SPAG4 | 5.893974 | 4.830153 | -1.06382 | 4.64E-24 | 4.08E-22 |
| TNNT1 | 3.725747 | 4.896583 | 1.170837 | 4.86E-14 | 5.64E-13 |
| ZNF486 | 7.961075 | 6.869875 | -1.0912 | 2.40E-15 | 3.55E-14 |
| PTHLH | 3.377518 | 4.878367 | 1.500849 | 3.26E-22 | 1.85E-20 |
| FSCN1 | 7.954421 | 9.116441 | 1.16202 | 9.38E-20 | 3.29E-18 |
| SLC44A4 | 6.625014 | 5.166342 | -1.45867 | 7.84E-15 | 1.06E-13 |
| ST3GAL5 | 6.752154 | 5.412143 | -1.34001 | 1.46E-23 | 1.12E-21 |
| KRT7 | 10.38956 | 8.454519 | -1.93504 | 5.88E-18 | 1.47E-16 |
| PLEKHA6 | 6.194452 | 5.172926 | -1.02153 | 3.37E-23 | 2.37E-21 |
| PADI3 | 7.690602 | 6.258571 | -1.43203 | 3.50E-09 | 1.68E-08 |
| UPK3B | 7.12911 | 5.487056 | -1.64205 | 9.02E-16 | 1.46E-14 |
| PLAUR | 4.6366 | 5.777728 | 1.141127 | 7.47E-27 | 1.29E-24 |
| TEAD4 | 4.325 | 5.43873 | 1.11373 | 8.46E-30 | 3.85E-27 |
| FXYD4 | 5.134664 | 4.000171 | -1.13449 | 1.12E-17 | 2.63E-16 |
| COL16A1 | 4.897121 | 6.093585 | 1.196464 | 2.63E-16 | 4.83E-15 |
| KRT1 | 2.96033 | 4.1125 | 1.15217 | 2.36E-13 | 2.41E-12 |
| OR7E91P | 5.377104 | 4.238809 | -1.13829 | 1.41E-20 | 5.70E-19 |
| GPR160 | 5.571949 | 4.279745 | -1.2922 | 3.08E-28 | 8.42E-26 |
| RNF128 | 6.990031 | 5.229691 | -1.76034 | 1.16E-27 | 2.63E-25 |
| DSP | 5.71551 | 6.898564 | 1.183054 | 4.13E-18 | 1.06E-16 |
| MT1E | 4.549416 | 5.757311 | 1.207895 | 5.53E-14 | 6.34E-13 |
| CLDN4 | 7.830511 | 6.784471 | -1.04604 | 2.55E-12 | 2.14E-11 |
| CAPS | 7.547769 | 6.027041 | -1.52073 | 1.03E-22 | 6.55E-21 |
| SPRR1B | 4.091008 | 6.174449 | 2.083441 | 1.01E-12 | 9.18E-12 |
| TBX3 | 7.685658 | 5.80502 | -1.88064 | 4.17E-32 | 4.28E-29 |
| SNCG | 9.452773 | 7.345748 | -2.10703 | 5.62E-26 | 7.35E-24 |
| SULT2A1 | 4.372092 | 3.357571 | -1.01452 | 1.05E-20 | 4.33E-19 |
| MAN1C1 | 5.179739 | 4.051203 | -1.12854 | 1.36E-13 | 1.44E-12 |
| CYP4F12 | 6.46815 | 4.913966 | -1.55418 | 1.64E-26 | 2.58E-24 |
| COL5A1 | 5.969751 | 7.20565 | 1.235899 | 1.14E-14 | 1.50E-13 |
| CAV1 | 5.407346 | 6.891964 | 1.484618 | 2.95E-22 | 1.70E-20 |
| IFITM3 | 8.886932 | 10.06482 | 1.177892 | 8.67E-18 | 2.08E-16 |
| CALD1 | 5.930934 | 7.147173 | 1.216239 | 5.45E-16 | 9.29E-15 |
| PVALB | 4.716499 | 3.549686 | -1.16681 | 7.74E-11 | 5.04E-10 |
| EVPL | 7.956671 | 6.924985 | -1.03169 | 7.52E-16 | 1.24E-14 |
| FBP1 | 8.723138 | 6.730941 | -1.9922 | 2.08E-31 | 1.77E-28 |
| CGN | 7.30633 | 6.011949 | -1.29438 | 7.12E-20 | 2.53E-18 |
| BCAT2 | 7.78344 | 6.616627 | -1.16681 | 4.90E-26 | 6.70E-24 |
| PCP4L1 | 5.105554 | 3.933509 | -1.17204 | 2.59E-12 | 2.17E-11 |
| SLITRK6 | 6.266769 | 4.989826 | -1.27694 | 8.62E-13 | 7.88E-12 |
| CTHRC1 | 5.169856 | 6.786484 | 1.616627 | 2.02E-21 | 9.76E-20 |
