# Supplementary figures and images for: Identification of a Pyroptosis-Related Gene Signature for Prediction of Overall Survival in Lung Adenocarcinoma
Source: J Oncol. 2021 Sep 30;2021:6365459. doi: 10.1155/2021/6365459 (PMC8497135; doi:10.1155/2021/6365459)

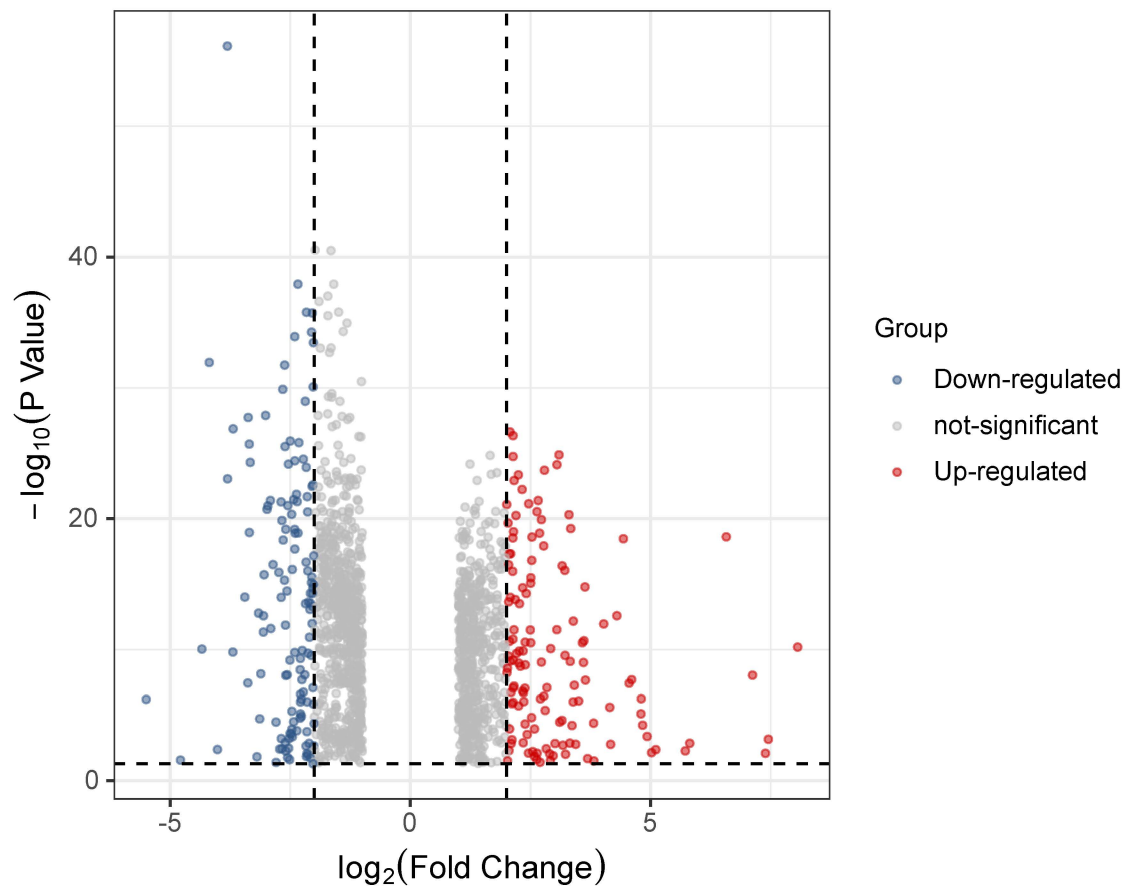

Supplement: Supplementary Materials — Supplementary File Table S1: 52 genes associated with pyroptosis from prior reviews. Supplementary File Table S2: 1458 DEGs associated with cluster 1 and cluster 2. Supplementary File Table S3: 13 genes identified by univariate regression. Supplementary File Table S4: 317 DEGs between low- and high-risk groups in TCGA cohort. Supplementary File Figure S1: An overview of the differential gene expression between the two pyroptosis-related clusters in TCGA cohort. [file 6365459.f1.zip › 6365459.f1/FigureS1.pdf]
